# Coupling of 5S RNP rotation with maturation of functional centers during large ribosomal subunit assembly

**DOI:** 10.1038/s41467-020-17534-5

**Published:** 2020-07-27

**Authors:** Jelena Micic, Yu Li, Shan Wu, Daniel Wilson, Beril Tutuncuoglu, Ning Gao, John L. Woolford

**Affiliations:** 1 Department of Biological Sciences, Carnegie Mellon University, Pittsburgh, PA USA; 2 State Key Laboratory of Membrane Biology, School of Life Science, Tsinghua University, Beijing, China; 3 Peking University-Tsinghua University-National Institute of Biological Sciences Joint Graduate Program, Beijing, China; 4 State Key Laboratory of Biocatalysis and Enzyme Engineering, Hubei Collaborative Innovation Center for Green Transformation of Bio-Resources, School of Life Sciences, Hubei University, Wuhan, China; 5 Department of Cellular and Molecular Pharmacology, University of California, San Francisco, CA USA; 6 State Key Laboratory of Membrane Biology, Peking-Tsinghua Center for Life Sciences, School of Life Sciences, Peking University, Beijing, China

**Keywords:** Protein folding, Ribosome, Cryoelectron microscopy

## Abstract

The protein composition and structure of assembling 60S ribosomal subunits undergo numerous changes as pre-ribosomes transition from the nucleolus to the nucleoplasm. This includes stable anchoring of the Rpf2 subcomplex containing 5S rRNA, rpL5, rpL11, Rpf2 and Rrs1, which initially docks onto the flexible domain V of rRNA at earlier stages of assembly. In this work, we tested the function of the C-terminal domain (CTD) of Rpf2 during these anchoring steps, by truncating this extension and assaying effects on middle stages of subunit maturation. The *rpf2Δ255-344* mutation affects proper folding of rRNA helices H68-70 during anchoring of the Rpf2 subcomplex. In addition, several assembly factors (AFs) are absent from pre-ribosomes or in altered conformations. Consequently, major remodeling events fail to occur: rotation of the 5S RNP, maturation of the peptidyl transferase center (PTC) and the nascent polypeptide exit tunnel (NPET), and export of assembling subunits to the cytoplasm.

## Abstract (web summary)

As assembling 60S subunits transit from the nucleolus to the nucleoplasm, they undergo significant changes in protein composition and structure. Here, the authors provide a structural view of interconnected events during the middle steps of assembly that include the maturation of the central protuberance, the peptidyltransferase center and the nascent polypeptide exit tunnel.

## Introduction

Ribosomes translate the genetic code in mRNA and catalyze the synthesis of proteins in all cells in nature. They are comprised of two subunits. In yeast, the large (60S) subunit includes 3 ribosomal RNAs (rRNAs) (25S, 5.8S, and 5S) and 46 ribosomal proteins (r-proteins), whereas the small (40S) subunit contains 1 rRNA (18S) and 33 r-proteins. Ribosomal subunit assembly involves transcription, modification, processing and folding of rRNA, and binding of r-proteins^1–6^. More than 200 assembly factors (AFs) facilitate accurate and efficient ribosome biogenesis, enabling proper growth and proliferation of cells^1,6^.

In 60S ribosomal subunits, 25S rRNA is divided into six phylogenetically conserved secondary structure domains. During assembly, folding and compaction of rRNA is coupled with stable incorporation of r-proteins, forming complex ribonucleoprotein (RNP) particles^7^. Assembly of 60S subunits is hierarchical; rRNA domains that form the convex solvent side of the subunit assemble first, followed by those that comprise the nascent polypeptide exit tunnel (NPET), and finally the central protuberance (CP) and the subunit interface containing the peptidyl transferase center (PTC)^8–11^.

Three out of four rRNAs (18S, 25S, and 5.8S) are transcribed by RNA polymerase I and processed from a single rRNA precursor (35S pre-rRNA), whereas 5S rRNA is transcribed separately by RNA polymerase III and is processed independently (Supplementary Fig. 1a)^12,13^. The 5S rRNA forms the 5S RNP complex with two r-proteins, rpL5 and rpL11, in the cytoplasm. This complex is imported to the nucleolus by the transport adaptor Syo1^14,15^ and binds to the portion of 25S rRNA domain V that becomes part of the CP in mature subunits^7^.

In metazoa, stable association of the 5S RNP with assembling 60S subunits is coordinated with cell growth and proliferation. When ribosomal assembly fails, the free 5S RNP binds to MDM2, an inhibitor of the tumor suppressor p53, and sequesters it in the nucleolus. This activates p53, cell cycle arrest, and eventually apoptosis. In addition, rpL11 inhibits other potential oncogenic properties of MDM2^16–18^.

The 5S RNP is a part of the Rpf2 subcomplex, together with a heterodimer of AFs Rpf2 and Rrs1^19–23^. Rpf2 and Rrs1 are necessary for initial docking of the 5S RNP with the assembling subunit, but the timing of their entry remains unclear^20^. Rpf2 and Rrs1 may assemble into the subunit before the 5S RNP or they may all associate at the same time^22,23^. All of them co-purify with early nucleolar assembly intermediates^20,24,25^. However, they cannot be visualized on particles by cryo-electron microscopy (cryo-EM) until the formation of Nog2 State 1/State F/Arx1 particles during late nucleolar stages (middle stages) of 60S subunit assembly (Supplementary Figs. 1b and 2a)^9,26,27^. This transition from a flexible/invisible state to a stable/visible state is designated as “anchoring”. Anchoring includes stable positioning of the Rpf2 subcomplex to the region that will become the CP and the specific rRNA rearrangements initiated by this positioning. Depletions of the Rpf2 subcomplex constituents do not affect biogenesis of 60S subunits until this anchoring occurs^20,28^. Thus, the Rpf2 subcomplex and rRNA domain V (helices H80, H82-88) with which it first associates are initially docked in a flexible state and do not influence assembly of nascent subunits until they become stably anchored within Nog2 particles.

Strikingly, when the Rpf2 subcomplex anchors onto assembling pre-60S subunits, the 5S RNP is in a ∼180°-rotated position compared with that in mature 60S subunits^26^. The adjacent rRNA helices (H80, H82-H88), as well as H38, H69, and H70, also display drastic conformational differences compared with mature subunits^26^. In order for 5S RNP rotation to occur, Rpf2 and Rrs1 must exit assembling subunits. Their exit appears to be initiated by binding of Sda1 in close proximity to the Rpf2-Rrs1 dimer and H68-H69. The exact mechanism of this process is still unclear. Sda1 then recruits the Rix1 subcomplex (Ipi1/Rix1/Ipi3) and the AAA+ ATPase Rea1. Their initial binding is thought to destabilize interactions between the 5S RNP, Rpf2-Rrs1, and Rsa4 to allow rotation of H38 and 5S RNP. These structural rearrangements may activate Rea1, which removes Rsa4 and itself using ATP hydrolysis. Finally, Nog2 must exit using its own GTPase activity to allow the nuclear export factor Nmd3 to replace it, thus enabling export competency (Supplementary Fig. 1b)^14,19,26,27,29–32^.

Importantly, during middle stages of 60S subunit assembly, other significant structural rearrangements also occur. They include the following: construction of future functional centers (PTC and NPET), removal of the rRNA spacer ITS2, and anchoring of rRNA domains IV and V to the main body of the subunit. Whether and how these significant rearrangements are coordinated and interconnected is unclear.

Intriguingly, the partially disordered C-terminal domain (CTD) of Rpf2 is positioned between the GTPase domain of Nog2 and helices H69-H71^27^. Here, we have truncated this extension to investigate its function in middle stages of assembly, in particular its potential involvement in stable anchoring of the Rpf2 subcomplex to the nascent pre-60S particle. Our results indicate that this long extension of Rpf2 does not appear to be necessary for docking of the Rpf2 subcomplex to rRNA domain V during the early stages of assembly, although a small effect cannot be ruled out. Instead, it is necessary, directly or through Nog2, for proper folding of H68-70 during anchoring. As a result, improper conformations of these helices affect binding of AFs necessary for rotation of the 5S RNP, maturation of the NPET, and export of nascent 60S subunits to the cytoplasm. H68-H70 are also involved in accurate formation of the PTC during subunit biogenesis, are part of inter-subunit bridges with the 40S ribosomal subunit, and are crucial for efficient protein synthesis^33–36^. In summary, conformational immaturity of these rRNA helices in the *rpf2Δ255-344* mutant strain blocks 60S ribosomal subunit assembly during late nucleolar steps.

## Results

### The CTD of Rpf2 is involved in 60S subunit assembly

To understand the role of the Rpf2 subcomplex during middle stages of subunit assembly, we made specific mutations in the AF Rpf2. We took advantage of a high-resolution cryo-EM structure of the Nog2 particle containing the 5S RNP, Rpf2, and Rrs1 (Supplementary Fig. 2a)^27^. Rpf2 consists of a globular domain that interacts extensively with Rrs1^21–23^ and an extended CTD (amino acids 255–344) (Supplementary Fig. 2b–d). Although amino acids 255–300 in the CTD of Rpf2 are highly conserved, amino acids 301–344 are not (Supplementary Fig. 3). The latter are predicted to be disordered and are not visible by cryo-EM in Nog2 particles, presumably due to their flexibility. The presence of an epitope tag at the C terminus of Rpf2 (*RPF2-TAP*) affects cell growth and production of 60S ribosomal subunits (Supplementary Fig. 4a), suggesting the importance of the CTD of Rpf2 in maturation of pre-60S ribosomes. The CTD of Rpf2 is positioned between the GTPase domain of Nog2 and rRNA helices H68-H71; thus, it may contribute to maturation of these helices (Supplementary Fig. 2b)^27^. Therefore, we deleted the entire CTD (amino acids 255–344) or only the disordered portion of the CTD (amino acids 310–344). We also created two clusters of mutations in conserved sequences of the CTD, which would potentially disrupt interactions between Rpf2 and Nog2, and Rpf2 and H68-71. The *rpf2-1* allele (cluster 1) contains two substitution mutations (I267A and M268A), whereas the *rpf2-2* allele (cluster 2) contains six mutations (I275A, M277A, G278A, Q280A, L282A, and L285A) (Supplementary Fig. 2c).

To assay the effects of these mutations, we expressed mutant proteins from plasmids in a strain conditional for expression of endogenous Rpf2 protein (*GAL-RPF2*).

**Fig. 1:**
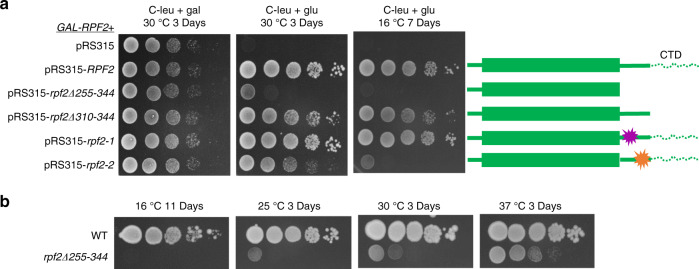
The CTD of Rpf2 is essential for cell growth at 16 °C. **a** Left: growth of yeast strains containing truncated or mutated Rpf2 protein on solid medium. Serial dilutions (1 : 10 to 1 : 10,000) were spotted onto selective solid medium containing either galactose or glucose and incubated at 30 °C and 16 °C. Right: schematic representation of truncations and mutations introduced into the CTD of Rpf2. Amino acids 301–344 are represented by dashed lines. **b** Growth of yeast strain containing the genomic allele of *rpf2Δ255-344* and its wild-type control. Serial dilutions (1 : 10 to 1 : 10,000) were spotted onto solid medium and incubated at 16, 25, 30, and 37 °C. Source data are provided as a Source Data file.

We assayed growth of the mutant strains on galactose- (where both wild-type *RPF2* and mutant *rpf2* are expressed) or glucose-containing solid media (where only mutant *rpf2* is expressed) at different temperatures. Removal of the entire CTD (*rpf2Δ255-344*) had a strong growth defect at all temperatures and was lethal at 16 °C. Truncation of the disordered portion of the CTD (*rpf2Δ310-344*) had a very slight growth defect at 16 °C. Thus, the conserved portion of the CTD appears to play a more important role in 60S subunit assembly than the disordered domain. The *rpf2-1* mutation did not affect growth. The *rpf2-2* mutant had a slight growth defect at 30 °C but growth was affected strongly at 16 °C, similar to the *rpf2Δ255-344* mutant (Fig. 1a).

Based on these growth defects, we focused on the *rpf2Δ255-344* mutant. To determine whether the truncated Rpf2 protein produced by this mutant was stable, we tagged both Rpf2 and rpf2Δ255-344 proteins with double Myc epitopes at their N termini. We tested the expression and stability of these proteins, as well as two other protein constituents of the Rpf2 subcomplex (rpL5 and rpL11), by western blotting of whole-cell lysates (Supplementary Fig. 4b). All assayed proteins were stably expressed.

To test the ability of the mutant Myc-rpf2Δ255-344 protein to enter pre-ribosomal subunits, we performed sucrose gradient fractionation. As expected, the levels of free 60S subunits were significantly lower relative to 40S subunits in the *rpf2Δ255-344* strain, compared with the wild-type strain, indicating a defect in 60S subunit assembly. In addition, polysomes (translating ribosomes) were decreased in the mutant strain (Supplementary Fig. 4c). Importantly, levels of the truncated rpf2 protein sedimenting with pre-60S particles compared with levels at the top of the gradient (free proteins and small complexes) were comparable to the wild-type protein (Supplementary Fig. 4d). Together, these results indicate that the truncated Rpf2 is stable and can enter pre-ribosomal particles at wild-type levels.

As the *rpf2Δ255-344* mutation is conditionally lethal (cold sensitive), we created a strain in which the genomic wild-type *RPF2* allele was replaced with the *rpf2Δ255-344* mutant allele. Yeast containing this mutant allele in the genome grew slowly at all temperatures and were inviable at 16 °C (Fig. 1b). Subsequent experiments were performed using yeast containing the genomic *rpf2Δ255-344* mutant strain and yeast expressing the plasmid-borne mutant allele, with identical results.

To examine at what step of the 60S subunit assembly pathway the *rpf2Δ255-344* mutant is blocked, we assayed levels of pre-rRNAs by northern blotting and primer extension assays. The mutant strain accumulated 27SB and 7S pre-rRNAs, indicating a defect in late nucleolar/nucleoplasmic (middle) stages of 60S subunit assembly, during the lifetime of Nog2 particles (Supplementary Figs. 1a and 4e–g).

To confirm that 60S subunit assembly in the *rpf2Δ255-344* mutant is blocked prior to subunit export from the nucleus to the cytoplasm, we assayed the localization of nascent pre-60S ribosomes, using the rpL25-eGFP reporter. In wild-type *RPF2* cells, rpL25eGFP was most abundantly present in the cytoplasm as part of translating ribosomes (Supplementary Fig. 5a, top panels). In the *rpf2Δ255-344* mutant, rpL25eGFP accumulated in the nucleus, with some nucleolar, but mostly nucleoplasmic localization (Supplementary Fig. 5a, bottom panels). This result indicates that middle stages of assembly are blocked, resulting in failure to export pre-60S particles to the cytoplasm.

### Truncation of the CTD of Rpf2 affects levels of specific AFs

**Fig. 2:**
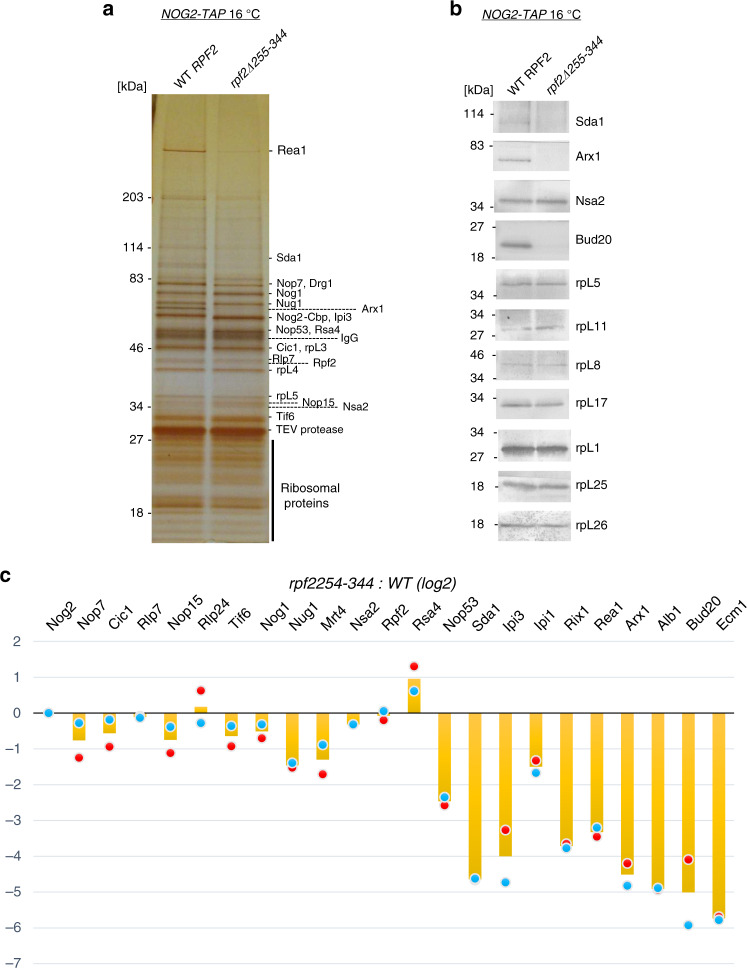
AFs necessary for 5S rotation and export are affected in the *rpf2Δ255-344* mutant. **a** SDS-PAGE of proteins in assembling 60S subunits from wild-type cells or from the *rpf2Δ255-344* mutant shifted to 16 °C for 5 h. Pre-ribosomal particles were purified using AF Nog2 as a bait and protein constituents were separated by electrophoresis and stained with silver. Molecular weight standards, the Nog2 bait protein Nog2-Cbp (calmodulin-binding peptide left behind after TEV cleavage), and relevant AFs are labeled. **b** Samples from **a** were subjected to western blotting using antibodies against specific proteins. All samples were derived from the same experiment and western blottings were processed in parallel. **c** Samples prepared as described in **a** were used for iTRAQ (semi-quantitative mass spectrometry) to quantify relative changes in levels of AFs in the genomic *rpf2Δ255-344* mutant compared with the wild-type strain. The ratios were normalized to levels of Nog2 (bait). The fold change is shown using bar graphs (orange) in log2 scale as an average of two biologically independent samples (*n* = 2) for the genomic *rpf2Δ255-344* mutant compared with the wild-type grown at 16 °C. Dot blots represent values for each biological replicate (red and blue). Source data are provided as a Source Data file.

To uncover changes in pre-ribosomal protein composition in the *rpf2Δ255-344* mutant, we affinity-purified pre-ribosomal particles from the middle stages of 60S subunit maturation and examined their protein constituents by SDS-polyacrylamide gel electrophoresis (PAGE), western blotting, and semi-quantitative mass spectrometry (iTRAQ) (Fig. 2 and Supplementary Fig. 5c). As Nog2 is present in pre-ribosomes during the middle stages of 60S subunit assembly (Supplementary Fig. 1b) and it accumulates in Nop7 particles purified from the *rpf2Δ255-344* mutant strain (indicating a block in assembly during the lifetime of Nog2 in pre-ribosomes) (Supplementary Fig. 5b), we used epitope-tagged Nog2 as a bait for these purifications.

Levels of AFs Sda1, the Rix1 subcomplex (Ipi1, Rix1/Ipi2, and Ipi3), and Rea1 decreased significantly in the Nog2 particles lacking the CTD of Rpf2 (Fig. 2). These AFs have been previously shown to be necessary for rotation of 5S RNP^19,37^. Rsa4, which requires Rea1 for its release from preribosomes, accumulated slightly. In addition, levels of nuclear export factors Arx1, Bud20, Ecm1, as well as the Arx1-associated protein Alb1, were also diminished, consistent with our observation that the mutant particles were not export competent (Fig. 2 and Supplementary Fig. 5a). Levels of Nop53 were also decreased in the *rpf2Δ255-344* mutant particles. As Nop53 recruits the exosome machinery to ITS2, processing of this spacer RNA may be affected in some assembling 60S particles lacking Nop53 (Fig. 2a, c)^29,38^.

### Cryo-EM of *rpf2Δ255-344* reveals a block in 5S RNP rotation

To assess changes in the structure of assembling large subunits, we performed cryo-EM analysis of pre-60S particles purified from the *rpf2Δ255-344* mutant strain. After several rounds of three-dimensional (3D) classification, we obtained three major structural classes (C1 to C3, 3.9–6.0 Å resolution) (Supplementary Fig. 6). Highly similar classes, C2 and C3, were combined for further refinement, providing an improved map (C4, 3.2 Å). Next, we built and refined an atomic model for the class C4 particles.

**Fig. 3:**
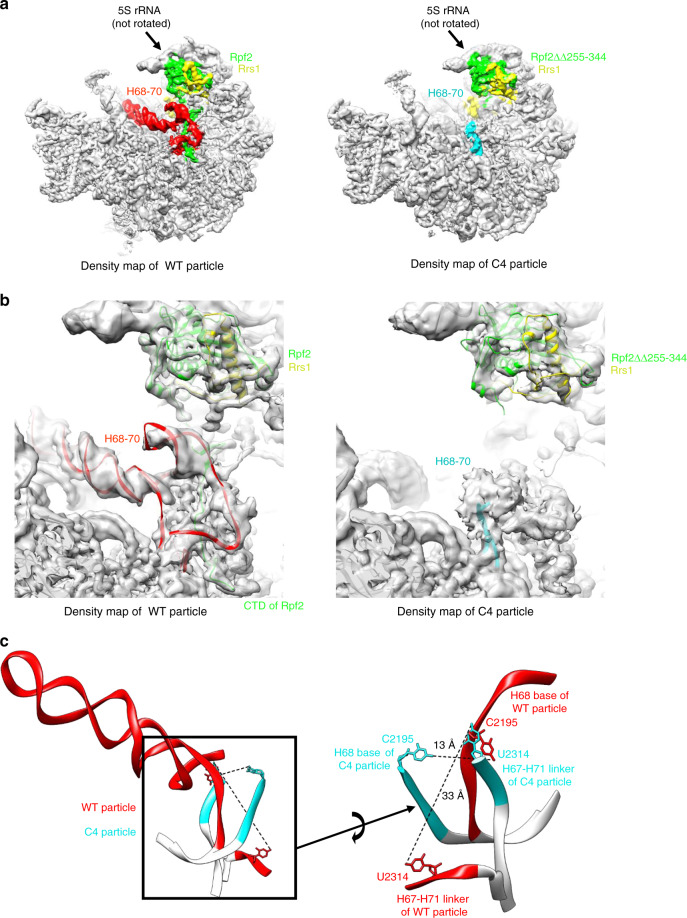
H68-70 are flexible and invisible in the absence of the CTD of Rpf2. **a** Subunit interface view of density maps from the wild-type particles and the C4 class of *rpf2Δ255-344* mutant particles. Densities for Rpf2, Rrs1, and H68-70 were colored using Color Zone in Chimera. **b** Atomic models for wild-type and C4 particles were fitted into density maps of wild-type and C4 particles, respectively. The C4 class of the *rpf2Δ255-344* particles is missing densities for the CTD of Rpf2 and for H68-H71, except for the base of H68 and the H67-H71 linker. **c** Structural comparison of atomic models of the wild-type particle and mutant C4 particle in the stem base region of H68. One strand of the base of H68 in the C4 mutant is deflected up to 13 Å, as indicated by the displacement of C2195, and the H67-H71 linker is deflected up to 33 Å.

Our cryo-EM results show that in all *rpf2Δ255-344* particles, densities for the truncated CTD of Rpf2 were missing, as expected (Fig. 3). Importantly, in contrast to the wild-type Nog2 sample, in which a small population of particles contains Sda1 (State 2)^27^, no Sda1-containing particles were observed in structural classification of the *rpf2Δ255-344* mutant pre-ribosomes. Consistent with the absence of Sda1, the 5S RNP was in its pre-rotated state in all classes of mutant particles (Fig. 3a and Supplementary Fig. 6b). Therefore, deletion of the CTD of Rpf2 blocks 5S RNP rotation.

### H68-H70 do not fold properly in the *rpf2Δ255-344* mutant

**Fig. 4:**
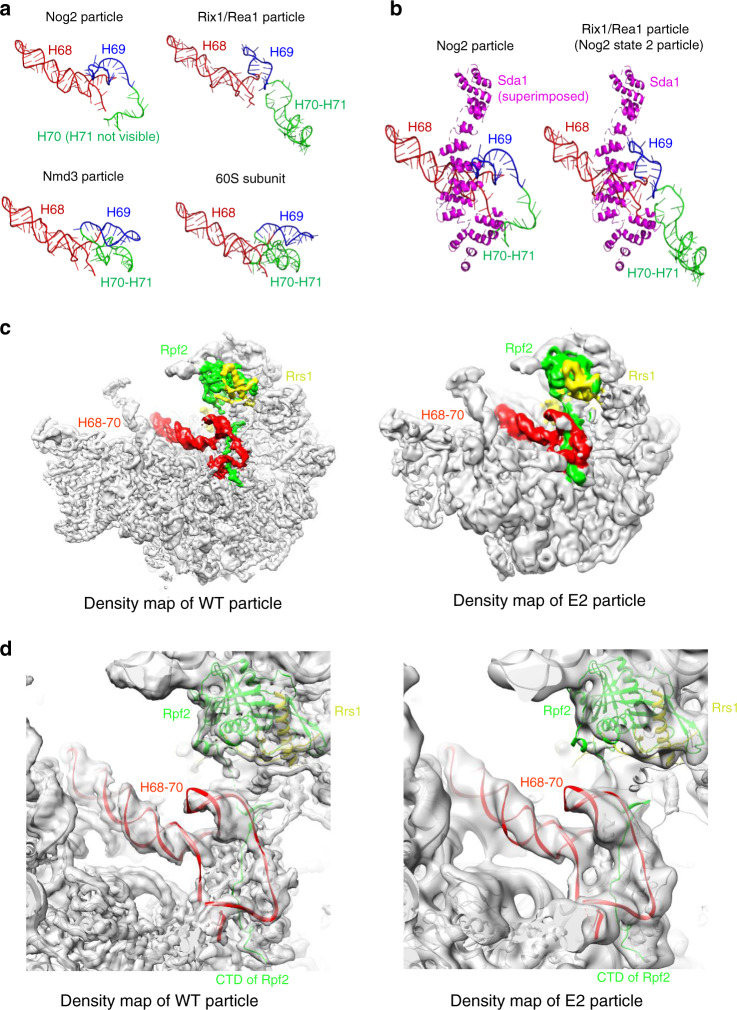
Changes in H68-70 are not caused by the absence of Sda1, which binds to H68-69. **a** Maturation of H68-H71 in wild-type cells during the transition from Nog2 particles to Rix1/Rea1 particles, then Nmd3 particles, and finally to mature 60S subunits (PDB IDs 3JCT [https://www.wwpdb.org/pdb?id=pdb_00003jct], 5FL8 [10.2210/pdb5FL8/pdb], 5APN [10.2210/pdb5APN/pdb], and 4V88 [10.2210/pdb4v88/pdb]). **b** Left: partial structure of Sda1 from the Rix1/Rea1 (Nog2 state 2) particle superimposed onto the structure of the Nog2 particle (PDB ID 3JCT [https://www.wwpdb.org/pdb?id=pdb_00003jct]). Sda1 is in close contact with H68 and H69 of the Nog2 particle. Right: Sda1 is in close contact with H68 and H69 in the Rix1/Rea1 particle (PBD ID 5FL8 [10.2210/pdb5FL8/pdb]), and the conformation of H69 is different from that in the Nog2 particle, presumably due to binding of Sda1. **c** Subunit interface view of density maps from the wild-type and E2 class of the Sda1-depleted particles. Densities for Rpf2, Rrs1 and H68-70 were colored using Color Zone in Chimera. No densities for H68-70 are missing in the E2 class compared with the wild-type Nog2 densities. **d** The atomic model for the wild-type particle was fitted into density maps of wild-type and E2 particles, respectively. Densities for Rpf2 and H68-70 are present in particles missing Sda1.

A second significant change in the *rpf2Δ255-344* mutant particles, compared with wild-type Nog2 particles, was the absence of densities for rRNA domain IV helices H68-H70 (Fig. 3). In wild-type Nog2 particles, the CTD of Rpf2 is in close contact with these helices (Supplementary Fig. 2d). Helices 68-69 and part of H70 can first be visualized by cryo-EM in Nog2 State 1 particles (H71 is not visible even in that state), but they are still in an immature conformation compared to that in mature 60 S subunits (Fig. 3a, b left and Fig. 4a). In particular, H69 needs to go through several consecutive steps of structural rearrangements during 60S subunit assembly, before it reaches its mature conformation (Fig. 4a and also see “Discussion”)^7,9,26,27,39^.

Densities for the entire H68-H69 and the visible portion of H70 were missing in the *rpf2Δ255-344* mutant (Fig. 3b). Some densities for the stem base of H68 were still visible (Fig. 3b), but significantly deflected compared to wild-type particles (Fig. 3c). These results indicate that H68-70 are not properly folded in mutant particles and explain their invisibility by cryo-EM.

### Folding of H68-H70 is not affected by the absence of Sda1

Importantly, one of the AFs that is significantly affected in the *rpf2Δ244-344* particles, Sda1, binds to rRNA helices H68-H69 in wild-type Nog2 state 2 particles (Fig. 4b)^27^. Mutation or depletion of Sda1 blocks 60S subunit assembly at the same step as truncation of the CTD of Rpf2^40^ (Supplementary Fig. 7a–d). As Sda1 does not assemble into pre-ribosomal subunits in the *rpf2Δ255-344* mutant, the flexible conformation of H68-H70 that we observed in this mutant may be the reason for the absence of Sda1, or else the consequence of its absence. To distinguish between these possibilities, we examined the structures of assembling pre-60S subunits upon directly depleting Sda1. We obtained two refined classes of particles, E1 and E2 (Supplementary Fig. 8). Even though these classes were in many general aspects similar to particles lacking the CTD of Rpf2 (compare Fig. 3a with Fig. 4c), the primary difference was that densities for H68-H70 were not missing when Sda1 was depleted and the conformations of H68, H69, and the visible part of H70 in the E2 class matched those in wild-type Nog2 particles (Fig. 4d). Thus, improper folding of H68-H70 is likely the reason for the absence of Sda1 in the *rpf2Δ255-344* mutant particles, not the consequence.

### The absence of the Rpf2 CTD affects H75 and AFs Nog2 and Rsa4

**Fig. 5:**
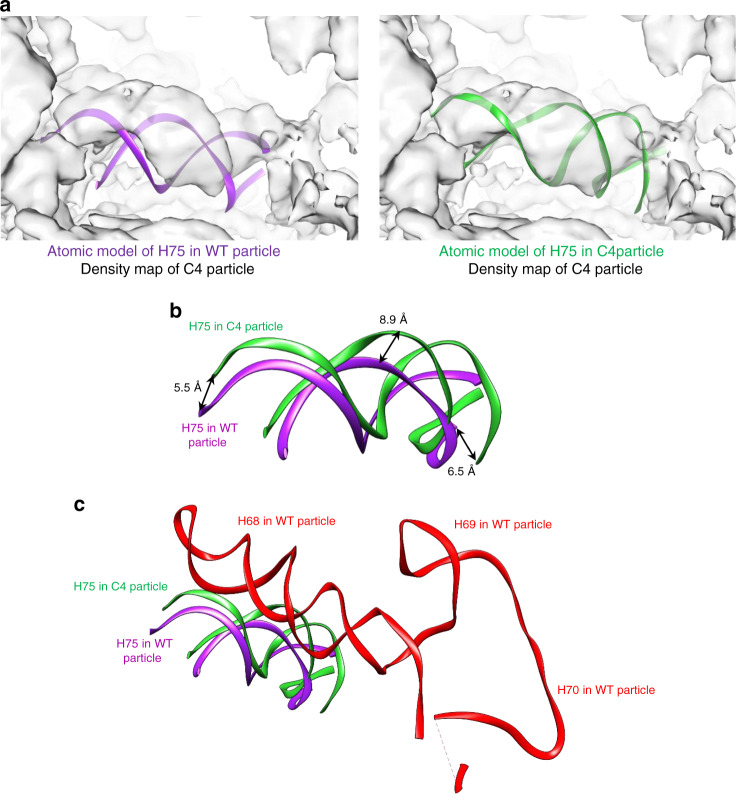
H75 is deflected in the C4 class of *rpf2Δ255-344* particles and clashes with H68. **a** The atomic model for H75 of wild-type particles (left) or the C4 class of *rpf2Δ255-344* mutant particles (right), was fitted into the density map of the C4 class of *rpf2Δ255-344* particles. **b** Atomic models for H75 in wild-type and C4 particles were aligned. H75 is deflected up to 8.9 Å in the C4 class compared with wild-type particles. **c** H75 in the C4 class of the *rpf2Δ255-344* particles clashes with the position of H68 in wild-type particles. H68-H70 are not visualized in the C4 class due to their flexibility.

To assess changes in conformations of pre-rRNAs and proteins, we aligned atomic models of wild-type Nog2 particles and the C4 class of the *rpf2Δ255-344* mutant Nog2 particles. The most significant change in the rRNA was the altered configuration of H75 in the mutant particles (Fig. 5a). This helix was deflected up to 9 Å in the mutant particles compared with its wild-type position (Fig. 5b). This displacement of H75 results in a steric clash with superimposed H68 (Fig. 5c). Considering that the rRNA helices underneath H75 remain in a similar conformation as in wild-type particles, it is likely that the conformational change in H75 in the C4 structure is a consequence, rather than a cause, of the failed docking of H68.

**Fig. 6:**
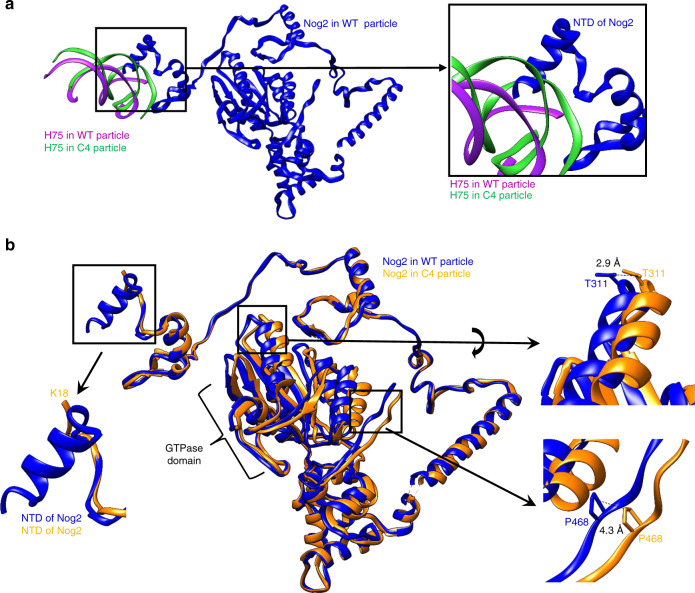
The conformation of Nog2 is altered in the C4 class of *rpf2Δ255-344* particles. **a** The NTD of Nog2 is docked in the groove of H75 near H68 in wild-type particles and may affect the conformations of these helices, or otherwise be affected by them. **b** Atomic models for Nog2 in wild-type and C4 particles were aligned. The NTD of Nog2 is flexible and invisible in the C4 class of the *rpf2Δ255-344* mutant particles. Conformational changes are visible in the GTPase domain of Nog2, where portions of the structure are deflected up to 4 Å.

Deflected H75 also clashes with the N-terminal extension of Nog2, with which it is in close contact (Fig. 6a). This might account for the observation that densities for the first 17 amino acids of Nog2 were missing in the C4 map (Fig. 6b). In addition, other portions of Nog2, the GTPase domain and the CTD, were in altered positions in the *rpf2Δ255-344* mutant (Fig. 6b). As the CTD of Rpf2 binds between Nog2 and H69-70, structural changes in the GTPase domain and CTD of Nog2 might be consequences of the absence of the C-terminal extension of Rpf2.

**Fig. 7:**
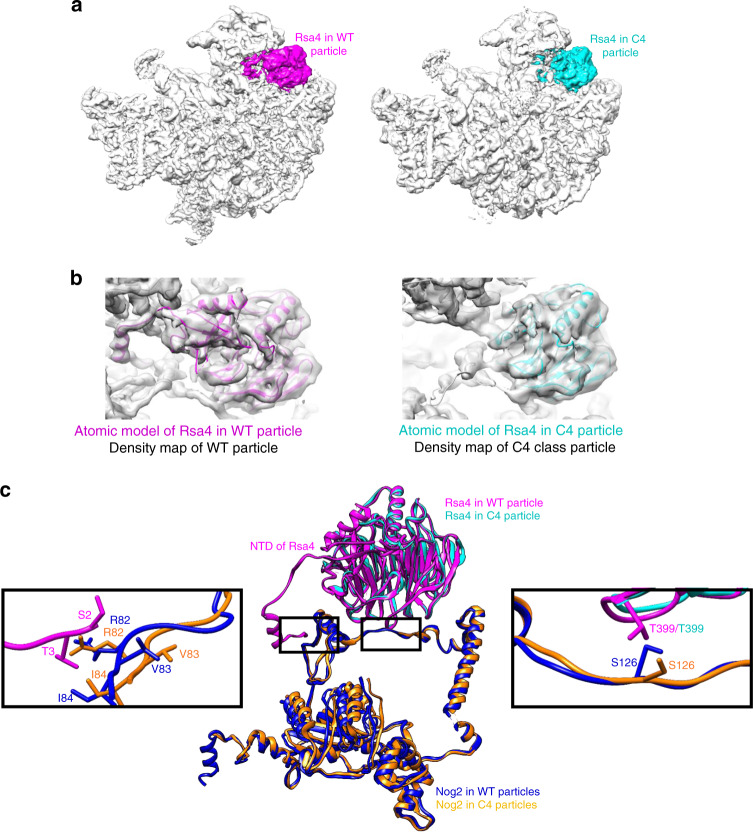
The NTD of Rsa4 is invisible, and likely flexible in the C4 class of *rpf2Δ255-344*. **a** The subunit interface view of density maps from the wild-type *RPF2* and the C4 class of *rpf2Δ255-344* mutant particles. Densities for Rsa4 were colored using Color Zone in Chimera. **b** Atomic models for Rsa4 from the wild-type and C4 class of the *rpf2Δ255-344* mutant were fitted into density maps of the wild-type and the C4 class mutant. The first 34 amino acids of the NTD of the Rsa4 are flexible and invisible in the absence of the CTD of Rpf2. **c** Atomic models for Rsa4 and Nog2 from the wild-type and the C4 class are colored and labeled. For clarity, only relevant side chains are shown. Conformational changes in Nog2 may affect Rsa4, because Nog2 and Rsa4 are in close contact at two points (rectangles): at the NTD and the globular domain of Rsa4. Enlarged rectangles show contact points between Rsa4 and Nog2.

All classes of particles obtained from the *rpf2Δ255-344* mutant were also lacking densities for the N-terminal domain (NTD) of Rsa4, indicating its flexibility (Fig. 7a, b). During early steps of 5S RNP rotation, Rsa4 must reposition itself; the signal for repositioning may be coming through interaction of Rsa4 with Nog2. The NTD of Rsa4 and its globular domain are in close contact with Nog2 (Fig. 7c)^27^. In the *rpf2Δ255-344* mutant, the conformation of Nog2 was slightly altered at both points of contact between Nog2 and Rsa4 (Fig. 7c), and might affect conformation of those regions of Rsa4.

### The absence of the CTD of Rpf2 may affect NPET maturation

**Fig. 8:**
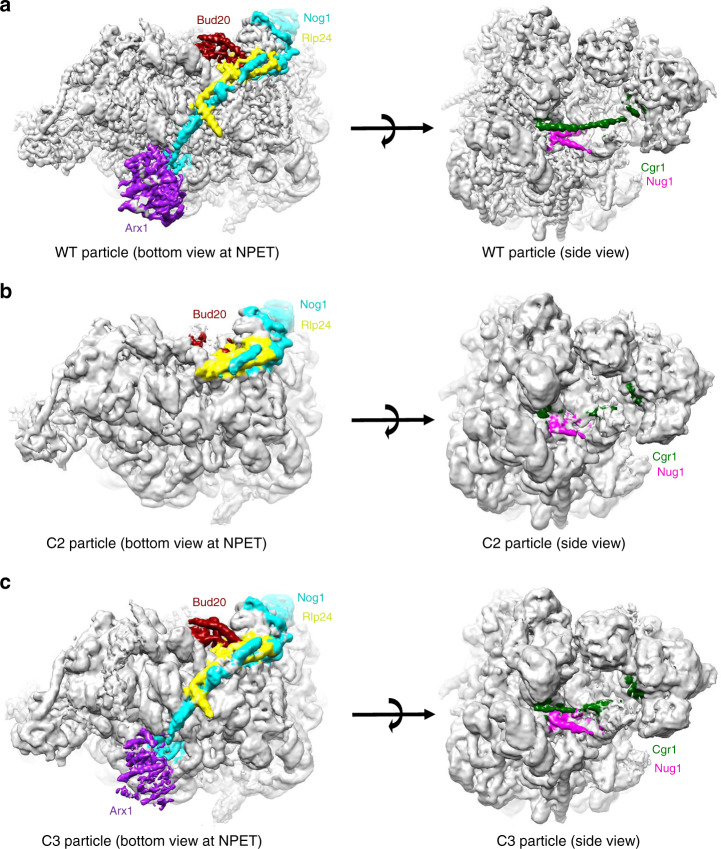
NPET maturation and nuclear export are affected in the *rpf2Δ255-344* mutant. **a** Subunit interface view (left) and side view (right) of the density map of wild-type particles. Densities for Nog1, Arx1, Bud20, Rlp24, Nug1, and Cgr1 were colored using Color Zone in Chimera. **b** Same as in **a** for the C2 class of *rpf2Δ255-344* mutant particles. Densities for the CTD of Nog1, all of Arx1, the CTD of Rlp24, and portions of Bud20, Nug1, and Cgr1 are absent in the C2 class. **c** Same as in **a** for the C3 class of *rpf2Δ255-344* mutant particles. Densities for Nog1, Arx1, Rlp24, Bud20, Nug1, and Cgr1 are present in the C3 class of *rpf2Δ255-344* mutant particles.

The CTD of the AF Nog1 (amino acids 595–647) occupies the NPET in wild-type Nog2 particles (Fig. 8a), but is absent from the NPET in the preceding (State E) wild-type particles, suggesting that this Nog1 extension is normally inserted into the NPET once Nog2 enters assembling 60S subunits^9,27^. The C2 and C3 classes of the *rpf2Δ255-344* mutant particles are highly similar, except for differences in densities for Nog1 and a few other AFs. Densities for the CTD of Nog1 were missing in the C2 class of *rpf2Δ255-344* mutant particles (representing 30% of particles), whereas the CTD of Nog1 was visible in the C3 class (representing 51% of particles) (Fig. 8). As densities for the CTD of Nog1 were also lacking in class C1 (19% of particles), these results show that in 49% of all particles, the CTD of Nog1 could not be stably inserted into the NPET and was in a flexible state. Thus, the *rpf2Δ255-344* mutant is likely to affect maturation of the NPET through the CTD of Nog1. In addition, densities for the CTD of Rlp24 were also very weak in the C2 class (Fig. 8b). Extensions of Nog1 and Rlp24 are in close proximity, and therefore flexibility of the CTD of Nog1 may affect the CTD of Rlp24.

### Nuclear export is blocked in the *rpf2Δ255-344* particles

Consistent with the lack of densities for the CTD of Nog1, export factor Arx1, which interacts with the CTD of Nog1 at the rim of the tunnel (amino acids 561–584 of Nog1), was missing from the C2 class of *rpf2Δ255-344* mutant particles. Another export factor, Bud20, also exhibited a sub-stoichiometric occupancy. This can partially account for the nuclear export block seen in the *rpf2Δ255-344* mutant strain (Fig. 8b and Supplementary Fig. 5a).

In addition, densities for Cgr1 and Nug1 were partially missing in the C2 class of *rpf2Δ254-344* particles (Fig. 8). Interestingly, densities for Cgr1 are not always visualized even in wild-type particles, suggesting that Cgr1 is loosely bound to the particles and that it may fall off during purification. Lack of the CTD of Rpf2 may further destabilize particles and weaken Cgr1 binding. Cgr1 is implicated in the stabilization of the rotated state of the 5S RNP^41^. Therefore, the absence of this protein would not add to defects in the *rpf2Δ254-344* mutant strain, as biogenesis in the absence of the CTD of Rpf2 is blocked at the step before 5S RNP rotation. Nug1 is a cation-dependent GTPase thought to be involved in formation of the PTC^42^; the reason for partial absence of its densities is not clear at this time.

In summary, all of these structural data demonstrating the presence or absence of AFs in pre-60S ribosomes are in an agreement with our SDS-PAGE, western blotting, and iTRAQ data.

### The C1 class may represent turnover intermediates

**Fig. 9:**
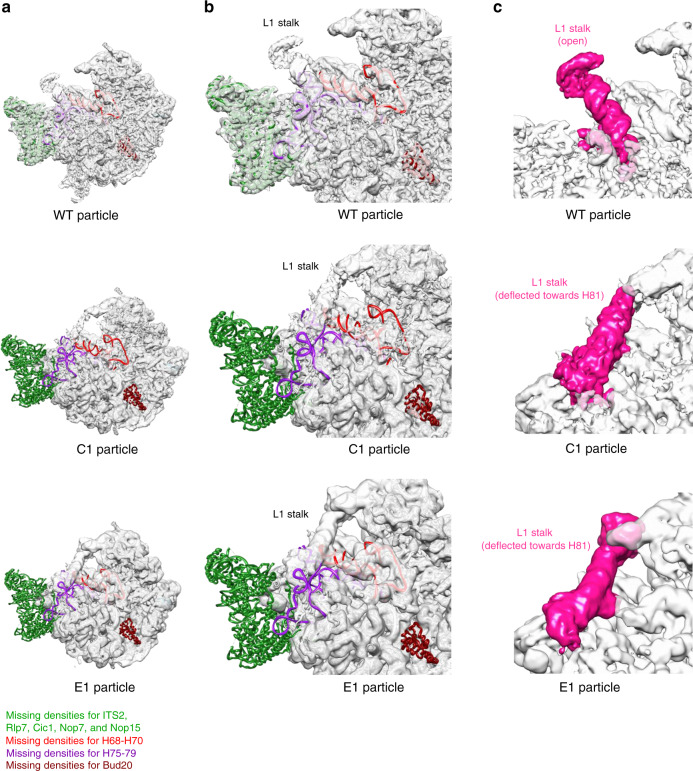
The C1 and E1 classes are unusual and presumably destined for turnover. **a** The subunit interface view of density maps from wild-type particles, the C1 class of *rpf2Δ255-344* mutant particles, and the E1 class of particles from Sda1-depleted yeast. The atomic model for wild-type particles was fitted into density maps of the wild-type and E1 class of mutant particles. The atomic model for the C4 class of mutant particles was fitted into the C1 class of mutant particles. **b** Enlarged view of the density maps from **a**. Densities for the ITS2 spacer rRNA and associated AFs Rlp7, Cic1, Nop7, Nop15, and Nop53 (all green), rRNA helices H75-79 (purple), and Bud20 (brown) are missing in the C1 and E1 classes. H68-70 (red) are missing in the C1 class, but are not significantly affected in the E1 class of mutant particles. (**c**) Enlarged portion of the density maps from (a). The L1 stalk (colored in pink) is in an unusual position in the C1 and E1 classes of particles, deflected toward H81.

The C1 class of particles had an unusual configuration, with highly flexible H68-70, largely displaced H75-79, and the absence of the ITS2 spacer RNA and associated AFs (Fig. 9a, b). In addition, the L1 stalk was in a closed position (Fig. 9b, c), sharply different from those observed in wild-type particles (refs. ^26,27,43,44^); it was deflected toward H81 instead of toward the 5S RNP. This unusual conformation of the L1 stalk resulted from the significant displacement of helices H75-79, which are located at the base of the L1 stalk (Fig. 9b). This C1 class may represent a turnover intermediate, and is not specific to the *rpf2Δ255-344* mutant strain. This class of particles was also observed upon depletion of Sda1 (with the exception of H69, which was not affected) (class E1, Fig. 9). In the *rpf2Δ255-344* mutant, the C1 class accounts for around 19% of all particles, whereas in the Sda1-depleted strain, 67% of the particles belong to this class.

## Discussion

Previously, we had shown that AF Rpf2 is necessary for initial docking of the 5S RNP into maturing 60S subunits^20^. Here we show that it is very unlikely that the CTD of Rpf2 is necessary for docking and early stages of subunit assembly. Instead, it appears that this extension is necessary during middle stages of assembly, more specifically for late stages of anchoring of the Rpf2 subcomplex to pre-60S subunits. We believe that the primary defect caused by the absence of the long C-terminal extension of Rpf2 is misfolding of rRNA helices H68-H70. This misfolding may be initiated by the loss of interactions between the CTD of Rpf2 and H68-70. Alternatively, it may be caused indirectly, through the adjacent GTPase Nog2, which may be affected by the truncation of the C-terminal extension of Rpf2, or by some other, still undiscovered path. Regardless, any perturbance in the neighborhood of helices H68-70 may prevent conformational maturation of these helices, and most likely cause a block in 60S subunit assembly.

**Fig. 10:**
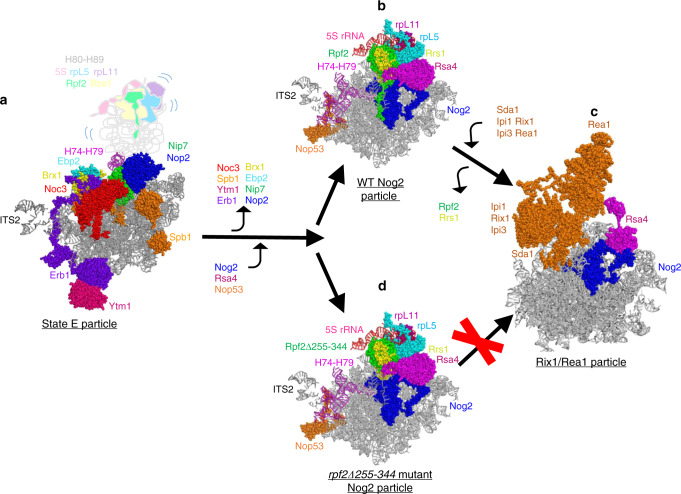
The CTD of Rpf2 is necessary as Nog2 particles transition to Rix1/Rea1 particles. **a** The 25S rRNA domain V and the Rpf2 subcomplex are in a flexible state and thus invisible (represented as the group in light colors) prior to rearrangements within the State E particle, including exit of labeled AFs from state E particles. **b** Stable anchoring of H74-H79, conformational changes in H68-70, as well as entry of Nog2, Rsa4, and Nop53, upon formation of Nog2 particles. **c** The Rix1/Rea1 particle is formed upon exit of the Rpf2-Rrs1 dimer, and entry of Sda1, the Rix1 subcomplex, and Rea1, as well as 5S RNP rotation. **d** In the absence of the CTD of Rpf2, the transition between the Nog2 and Rix1/Rea1 particles does not occur. For simplicity and clarity, only relevant AFs are shown and colored, and all r-proteins except rpL5 and rpL11 are omitted. Only partial densities for Sda1, Rea1, and the Rix1 subcomplex are available.

Sda1, necessary for 5S RNP rotation, binds to H68 and H69; therefore, the improper conformation of these helices is likely preventing binding of Sda1. The inability of Sda1 to bind affects association of the Rix1 subcomplex and Rea1, and thus rotation of the 5S RNP (Fig. 10). As a result, rotation of the 5S RNP cannot occur.

In wild-type cells, once the Rpf2 subcomplex is stably anchored (together with rRNA domain V) to the main body of assembling 60S subunits, H68-H70 can be visualized for the first time by cryo-EM in State 1 Nog2 particles, but still in an immature conformation compared to that in mature 60S subunits^7,9,26,27,39^. In particular, H69 needs to go through several structural rearrangements during 60 S subunit assembly before it reaches its mature position (Fig. 4a). Presumably, these stepwise conformational changes of H69 are mediated at least in part by sequential binding of proximal AFs, including Rpf2, Nog2, Sda1, Nmd3, and Lsg1, and by EngA during bacterial 50S subunit assembly^11,19,27,33,44,45^.

H69 is universally conserved. In bacteria, its deletion has a dominant lethal phenotype^35,36^. H69 is an integral part of the PTC. The closing loop of H69 contacts the mRNA decoding center of the small subunit, while its stem forms a portion of the P site in the large subunit. H69 is a critical part of the B2a inter-subunit bridge that communicates with h44 of the small ribosomal subunit^34,35^. In mature subunits, H69 is a potent regulator of translation as it alters small subunit rotation dynamics^36^. Considering the importance of H69, it is clear why mechanisms have evolved to keep this helix in an immature conformation until the 60S subunit is fully formed. Thus, the controlled maturation of H69 is essential for both proper formation and function of 60S subunits.

H71, similar to H69, goes through significant rearrangements during 60S subunit assembly (Fig. 4a)^19,27^. However, H71 is not yet visible in Nog2 particles, which prevents us from ascertaining whether this helix is also affected in the *rpf2Δ255-344* mutant strain.

H75 is significantly deflected from its native position in *rpf2Δ255-344* mutant particles (Fig. 5a, b). The flexibility of H68 is likely destabilizing H75 and allowing its deflection compared with wild-type particles (Fig. 5c). As the N-terminal extension of Nog2 is docked in the groove of H75, its flexibility may affect the conformation of H75, or vice versa (Fig. 6a).

Other conformational changes in Nog2 affect its GTPase and CTD domains. Points of contact among the CTD of Rpf2, H68-H70, and Nog2 are close to the Nog2 GTPase domain (Supplementary Fig. 1b, c). This may explain the phenotype of the *rpf2-2* mutant (Fig. 1a and Supplementary Fig. 7e–g), mutations in which target interactions of the Rpf2 extension with the Nog2 GTPase domain. These mutant amino acids can also affect interactions between the Rpf2 CTD and H68-70, since they are in close proximity to junctions of these helices. The altered configuration of Nog2 might affect the conformation of H68-70, or vice versa, the conformation of Nog2 may be affected by these RNA helices. The absence of any of these interactions may affect Nog2 in a way that prevents activation of its GTPase activity and exit from assembling 60S subunits.

In the absence of the CTD of Rpf2, the CTD of Nog1 does not enter the NPET in 49% of particles. It is currently unclear what the signal is for entry of this extension of Nog1 into the NPET. However, it has been proposed that the NPET and PTC may communicate with each other^46^. Thus, the incorrect conformation of H68-70 may be transmitted to the NPET (possibly through H75) and prevent further steps in NPET maturation (such as insertion of the Nog1 CTD) through steric changes in rRNA conformation. Alternatively, the block caused by the absence of the CTD of Rpf2 may block assembly upstream of entry of the Nog1 CTD into the NPET.

Processing of the ITS2 spacer RNA seems to proceed normally in at least some portion of these mutant particles, consistent with previous reports that 5S RNP rotation and ITS2 removal can occur independently from each other^41,47–49^. In the majority of particles, however, the block caused by truncation of the C-terminal extension of Rpf2 likely halts subunit assembly before the processing of ITS2 would normally occur. That is probably the reason why most particles still contain densities for ITS2 and its associated proteins.

Anchoring of 25S rRNA domain V and the Rpf2 subcomplex containing the 5S RNP can only occur after (1) Rea1 removes Ytm1 and Erb1, (2) Has1, Drs1, Nop2, Nip7, Ebp2, Brx1, Spb1, and Noc3 dissociate from assembling subunits, and (3) H74-79 anchor to the main body of the subunit (Fig. 10)^6,9^. We propose that the signal that stable anchoring of the Rpf2 subcomplex has occurred is transmitted through the CTD of Rpf2. The CTD of Rpf2 stabilizes H69 in a structurally defined intermediate conformation, and the subsequent binding of Sda1 rearranges H69 into its next conformation (Fig. 4a, b). Thus, the absence of the CTD of Rpf2 may prevent H69 from reaching its stable intermediate conformation, and may prevent H68 from reaching the near-mature conformation needed for Sda1 binding. Overexpression of Sda1 failed to suppress the slow growth phenotype of the *rpf2Δ255-344* mutant strain at 16 °C (Supplementary Fig. 7h), indicating that the block caused by the absence of Rpf2 occurs upstream of the entry of Sda1 into assembling 60S subunits.

The yields of pre-ribosomes purified from the *rpf2Δ255-344* mutant strain are slightly lower than those from the wild-type strain. This suggests that a small percentage of particles are turned over in the *rpf2Δ255-344* mutant strain before they could be detected by SDS-PAGE or visualized by cryo-EM. In addition, it is likely that the “bad particles” that we observed by cryo-EM in this mutant are destined for turnover. We speculate that the reason for this turnover is the unfolding of H68-70 in the absence of the CTD of Rpf2, and that this specific rRNA unfolding may trigger the turnover machinery. This explains why these bad particles are not as prevalent in the strain in which Sda1 is depleted, as H68-70 are not misfolded in that strain.

In summary, 5S RNP rotation appears to be coupled with maturation of functional centers during middle stages of large ribosomal subunit assembly. The nature of this coupling is not yet clear, but it is likely that H68-70 play a crucial role in it. When anchoring of the Rpf2 subcomplex occurs properly, a signal may propagate through the maturing subunit via the C-terminal extension of Rpf2 to Nog2 and H68-H70. Since conformational maturation of H68-70 is a prerequisite for entering the next major stages of subunit biogenesis, 5S RNP rotation and nuclear export, the failure of H68-H70 to fold properly triggers a series of downstream events that block progression of 60S subunit biogenesis, including NPET maturation and PTC assembly. Therefore, we speculate that conformational maturation of H68-70 plays a critical role in the middle steps of assembly and that proper folding of these helices is necessary not only for function of the mature 60S subunit, but is also vital during its biogenesis.

## Methods

### Construction of yeast strains and mutagenesis

Yeast strains and plasmids used in this study are listed in Supplementary Tables 1 and 2. A yeast strain conditional for expression of *RPF2* was constructed by replacing the endogenous promoter of *RPF2* with the galactose-inducible, glucose-repressible *GAL1* promoter including an in-frame insertion of the triple hemagglutinin (3HA) epitope. The template used for PCR was pFA6a-TRP1-pGAL1-3HA^50^. Sequences of oligonucleotides used for the PCR are available upon request. The correct size for the PCR product was confirmed by agarose gel electrophoresis. PCR products were purified and transformed into a wild-type strain, and the transformants were selected on C-trp + gal solid media. Replacement of the promoter was confirmed by colony PCR (sequences of oligonucleotides used are available upon request), and expression of the 3HA epitope in frame with the *RPF2* was confirmed by western blotting using anti-HA antisera (Thermo Fisher Scientific). The *GAL-SDA1* strain conditional for expression of Sda1 was a gift from Dr. Douglas R. Kellogg. Strains expressing TAP-tagged Nop7 or Nog2 were generated by replacing the stop codon of either *NOP7* or *NOG2* with the TAP-tag. The TAP-tag plasmid^51^ was used to amplify the TAP-tag and sequences immediately upstream or downstream of stop codons (sequences of oligonucleotides are available upon request). The correct sizes of the PCR products were confirmed by agarose gel electrophoresis, PCR products were purified and transformed into wild-type or *GAL-RPF2* strains. Transformants were selected on C-ura or C-ura+gal solid media. The presence of the C-terminal tag was confirmed by colony PCR (sequences of oligonucleotides used are available upon request), and the expression of the tagged protein by western blotting using anti-TAP antisera (Promega).

A strain expressing the Rrs1-3HA protein was generated by replacing the stop codon of *RRS1* by the 3HA sequence in frame. The plasmid pFA6a-3HA-HIS3MX6^50^ was used to amplify the 3HA tag and sequences immediately upstream or downstream of the stop codon of *RRS1* (sequences of oligonucleotides are available upon request). The correct size of the PCR product was confirmed by agarose gel electrophoresis, and the PCR products were purified and transformed into the *GAL-RRF2 NOP7-TAP* strain. Transformants were selected on C-his+gal solid media. The presence of the C-terminal tag was confirmed by colony PCR (sequences of oligonucleotides used are available upon request), and the expression of the tagged protein by western blotting using anti-HA antisera (Thermo Fisher Scientific).

A plasmid expressing Rpf2 was generated by cloning the open reading frame plus ~700 nucleotides upstream and 150 nucleotides downstream into the pRS315 plasmid at the multiple cloning site. Correct in-frame insertion was confirmed by sequencing (Genewiz).

Mutations and truncations in *RPF2* (*rpf2Δ255-344*, *rpf2Δ310-344*, *rpf2-1*, and *rpf2-2*) were introduced into the pRS315-*RPF2* plasmid, using the QuikChange II Site-directed mutagenesis kit (Agilent), as per the manufacturer’s protocol. Mutations were confirmed by sequencing (Genewiz). Sequences of oligonucleotides are available upon request.

Plasmids expressing MYC-tagged versions of *RPF2* and *rpf2Δ255-344* were generated by Epoch Life Science, Inc. Sequences for two Myc tags were inserted at the 5′*-*end of either gene in the pRS315 vector and correct in-frame insertion was confirmed by sequencing.

A diploid strain heterozygous for the *rpf2*Δ*255-344* mutant allele expressing a truncation of the CTD of Rpf2 was constructed by truncating one of the alleles of the *RPF2* by homologous recombination of the PCR product lacking codons for amino acids 255-344 and containing sequences immediately upstream and downstream from them. The plasmid used as a template was pFA6a-kanMX6^50^. Sequences of oligonucleotides are available upon request. The correct size of the PCR product was confirmed by agarose gel electrophoresis, and the PCR products were purified and transformed into the wild-type strain. Transformants were selected on YEPD + G418 solid media. Diploid candidates were sporulated at 30 °C and tetrads were dissected. Two out of four tetrads were very slow growing. The absence of the C-terminal extension of Rpf2 in slow growing tetrads was confirmed by colony PCR and sequencing of the PCR product that amplified the genomic region of the truncation. Sequences of oligonucleotides used are available upon request. As the absence of the Rpf2 CTD causes a growth defect even at 30 °C, we used the wild-type *RPF2* strain shifted to 16 °C as a control in our experiments.

### Growth of yeast strains

Yeast strains were grown at 30 °C in either YEPGal (2% galactose, 2% peptone, and 1% yeast extract) or YEPGlu (2% dextrose, 2% peptone, and 1% yeast extract). The conditional strains containing plasmids were grown in selective media (C-leu + gal) containing galactose and were shifted to selective media (C-leu + glu) containing glucose for 17 h. The genomic mutant *rpf2Δ255-344* strain and its corresponding wild-type strain were grown in YEPGlu media at 30 °C and shifted to 16 °C for 5 h.

### Sucrose gradient assays of ribosomes and polyribosomes

Cultures (100 ml) of *RPF2-TAP* and *RRS1-TAP* strains were grown in YEPGlu liquid media at 30 °C to an OD_600_ of 0.7. Cycloheximide (5 mg) was added to cultures 30 min before collecting cells. Extracts were made using lysis buffer (10 mM Tris-HCl pH 7.5, 0.1 M NaCl, 30 mM MgCl_2_, 50 μg/ml cycloheximide, 200 μg/ml heparin, and 0.2% diethyl pyrocarbonate). Cells were vortexed eight times for 30 s with glass beads (0.5 mm diameter, Biospec Products), kept on ice in between vortexing, and clarified by two consecutive centrifugations. Forty OD_254_ units of whole-cell extracts were layered on 7–47% (w/v) sucrose gradients, and pre-ribosomes, ribosomes, and polyribosomes were fractionated according to the manufacturer’s protocol (Teledyne ISCO). A Teledyne ISCO Foxy R1 density gradient fractionator was used to fractionate and analyze gradients with continuous monitoring at OD_254_.

For assaying the *rpf2Δ255-344* mutant strain and the corresponding wild-type control strain, cells were grown in YEPGal overnight and those starter cultures were used to inoculate 250 mL cultures in YEPGlu, to deplete endogenous Rpf2. Cells were prepared for sucrose gradient fractionation as described above.

### Assaying pre-rRNA processing

Northern hybridization and primer extension were used to assay steady-state levels of pre-rRNAs and rRNAs. Strains were grown in either galactose- or glucose-containing medium and 10–20 mL of cultures were collected and used for RNA extractions. Cells were resuspended in 300 µL of RNA buffer (500 mM NaCl, 200 mM Tris-HCl pH 7.5, 10 mM EDTA) and 300 µL of PCI (phenol : chloroform : iso amyl alcohol 50 : 49 : 1) was added to each sample. Cells were vortexed eight times for 30 s in the presence of glass beads (0.5 mm diameter, Biospec Products) and incubated on ice in between vortexing. Samples were centrifuged and the top layers were collected. RNA was precipitated with 100% ethanol overnight at −20 °C. Samples were washed with 70% ethanol and resuspended in water. A Nano Drop 2000C spectrophotometer (Thermo Scientific) was used to quantify amounts of total RNA extracted from cells and 5 μg of RNA were used per sample for each reaction.

For northern hybridization, samples were mixed with two volumes of sample buffer (8% formaldehyde, 1.3× MOPS buffer (1 mM sodium EDTA, 20 mM 3-(*N*-morpholino propane sulfonic acid pH 7.5, 8 mM sodium acetate)), 65% formamide, 0.02% xylene cyanol, and 0.1% bromophenol blue), and subjected to electrophoresis on a 1.2% agarose gel for 5.5 h at 150 V, with continuous recirculation of 1× MOPS buffer. RNA was transferred by capillary action from the gel to a Zeta-Probe blotting membrane (Biorad). Oligonucleotides were end-labeled using 32Pγ-ATP and polynucleotide kinase, and hybridized with the membrane at 47–48 °C overnight in hybridization buffer (1% bovine serine albumin, 0.5 mM Na_2_HPO_4_•7H_2_O/H_3_PO_4_ pH 7.2, 1 mM EDTA pH 8, and 5% SDS). Blots were washed three times in wash buffer (3 M NaCl, 300 mM Na citrate pH 7.0, and 0.2% SDS) at room temperature and were exposed to X-ray film for one to several days, depending on the abundance of the RNA species detected.

For northern hybridization of small molecular weight RNAs (7S, 5.8S, and 5S), RNA samples were mixed with an equal volume of sample buffer (0.1× TBE buffer, 10 M urea, 0.1% xylene cyanol, 0.1% bromophenol blue) and subjected to electrophoresis on a 5% acrylamide/7 M urea gel for 4 h at 120 mA. Following electrophoresis, gels were electroblotted to a Nytran N membrane (GE Healthcare Life Sciences) using a Trans-Blot Plus Cell (Biorad), hybridized with an end-labeled oligonucleotide, washed, and exposed to X-ray film, as described above.

Primer extension assays were performed as follows: radiolabeled oligonucleotide primers complementary to 27S pre-rRNA were annealed to RNAs for 10 min at 46 °C. Reverse transcriptase, RNasin, and deoxynucleoside triphosphates (Promega) were added to the mixture and incubated for 40 min at 46 °C. RNA was hydrolyzed with NaOH and EDTA for 30 min to 1 h at 55 °C. The reaction was neutralized with HCl and DNA was precipitated with glycogen, NH_4_OAc, and 100% ethanol. DNA was suspended in DNA dye (95% formamide, 0.05% xylene cyanol, 0.05% bromophenol blue in 20 mM EDTA pH 8.0) and subjected to electrophoresis on a 6% polyacrylamide-urea sequencing gel. Gels were dried and directly exposed to film for autoradiography for one to several days.

Oligonucleotide probes or primers are listed in ref. ^52^ (Table 4 and Fig. 6).

### Fluorescence microscopy

A reporter system that expresses the nucleolar marker mRFP-Nop1 and the L25-enhanced green fluorescent protein (L25-eGFP) (a gift from J. Bassler) was used to visualize a block in nucleolar release and nuclear export of assembling 60S ribosomal subunits. Strains expressing truncated Rpf2 protein or lacking Sda1 were transformed with the pRS316-*RPL25eGFP*/mRFP-*NOP1* plasmid, grown in the appropriate media lacking uracil, and shifted to either 16 °C for 5 h (*rpf2Δ255-344*) or to glucose-containing media for 17 h (*GAL-SDA1*). Cells were anchored to MaTek plates using 20–30 µL of 1 mg/mL concanavalin A (ConA, Fisher Scientific). The ConA was dried on plates for 30–45 min before adding cells. Cell images were obtained by a Zeiss LSM 880 laser scanning confocal microscope at ×600 magnification. Images were acquired using ZEN software (blue edition, by Zeiss) and the images were processed using Fiji for Mac OSX (National Institutes of Health).

### Affinity purifications of assembling 60S subunits

Ribosome assembly intermediates were affinity-purified from whole-cell extracts with magnetic Dynabeads (Thermo Fisher Scientific), using TAP-tagged AFs Nop7 or Nog2. Cultures (250 mL) were grown either in galactose- or glucose-containing liquid media to an OD_600_ of 0.7–0.9. Cells were collected and resuspended in 3.5 mL of Lysis Buffer (50 mM Tris-HCl pH 7.5, 150 mM NaCl, 10 mM MgCl_2_, 0.075% detergent octylphenoxypolyethoxyethanol), and subjected to vortexing with glass beads (0.5 mm diameter, Biospec Products) eight times for 30 s, with incubation on ice in between vortexing. Extracts were clarified by centrifugation and bound to IgG-coated Dynabeads at 4 °C for 1 h. Beads were washed three times with the Lysis buffer and pre-ribosomes were eluted by cleaving the TEV protease site within the TAP-tag, using 1–2 µL of TEV Protease (Thermo Fisher Scientific). Proteins were precipitated with 10% trichloroacetic acid (TCA), resuspended in SDS sample buffer and separated by SDS-PAGE on 4–20% Tris-Glycine Novex gels (Thermo Fisher Scientific).

### Western hybridization analysis

Proteins from whole-cell extracts or from purified pre-ribosomes were separated on SDS-PAGE gels, electroblotted to the Amersham Protran supported 0.45 µm NC membrane (GE Healthcare Life Sciences), and assayed by western blot analysis. To conserve antiserum by using a lower volume of blotting buffer and to enable detection of multiple proteins on one blot, nitrocellulose membranes were cut into smaller sections based on the known mobility of the different proteins. As Nog2 co-migrates with IgG on 4–20% Tris-Glycine Novex gels, NuPage 4–12% Bis-Tris gels (Thermo Fisher Scientific) were used to assay Nog2 protein by western blotting. TAP-tagged proteins were detected using alkaline phosphatase conjugated to IgG (Pierce). HA-tagged proteins were identified with mouse monoclonal antibody 12CA5 (Thermo Fisher Scientific) and Myc-tagged proteins with 9e10 antibody (Sigma-Aldrich). Otherwise, antibodies specific for r-proteins or AFs were used. Alkaline-phosphatase-conjugated anti-mouse or anti-rabbit secondary antibodies (Promega) were used and colorimetric detection was performed using NBT and BCIP (Promega).

### Analysis of pre-60S subunits by iTRAQ

For semi-quantitative mass spectrometry (iTRAQ), pre-ribosomes were purified as described above, with the following modifications. Cell pellets were resuspended in TNM150 buffer (50 mM Tris-HCl pH 7.5, 150 mM NaCl, 1.5 mM MgCl_2_, 0.1% NP-40, and 5 mM 2-mercaptoethanol (Sigma-Aldrich)). After incubation of the lysates with IgG-coated Dynabeads for 1 h at 4 °C, NP-40 was omitted from the buffer for all later steps. Purified samples were sent to the Penn State Hershey Core Research Facilities for trypsin digestion and 8-plex labeling with iTRAQ reagents 113, 114, 115, 116, 117, 118, 119, and 121 (Applied Biosystems). Peptides were separated by two-dimensional (2D) liquid chromatography and parent ions were identified on a Sciex 5600 liquid chromatography mass spectrometer system. Protein Pilot 5.0 was used to obtain iTRAQ ratios as an average of all peptides for each protein. Proteins identified with >99.9% confidence were used for further data analysis. Data were normalized to the change in ratio of the Nog2 bait protein.

### Purifications of pre-60S subunits for cryo-EM

Pre-ribosomes were purified as described above, with the following modifications: the same protocol as above was used, but the eluates were not precipitated using TCA. Instead, they were concentrated using Amicon-Ultra 0.5 mL centrifugal filters, Ultracel-100K (Millipore).

### Cryo-EM data acquisition

Vitrified specimens were prepared by adding 4 μl samples of *rpf2Δ255-344* or *GAL-SDA1* particles at a concentration of ~150 nM to a glow-discharged holey carbon grid (Quantifoil R2/2) covered with a freshly made thin carbon film. Grids were blotted for 1 s and plunge-frozen into liquid ethane using an FEI Vitrobot Mark IV (4 °C and 100% humidity). The cryo-grids were initially screened at a nominal magnification of ×92,000 in an FEI Talos Arctica microscope, operated with an acceleration voltage of 200 kV. Good-quality grids were transferred to an FEI Titan Krios electron microscope that was operating at 300 kV and images were recorded using a K2 Summit direct electron detector (Gatan) in counting mode at a nominal magnification of ×130,000, corresponding to a pixel size of 1.057 Å at the object scale and with the defocus varying from −1.0 to −2.0 μm. Coma-free alignment was manually optimized and parallel illumination was verified before data collection. All micrographs obtained with the K2 camera were collected semi-automatically by SerialEM^53^, under low-dose conditions. Each micrograph was dose fractionated to 32 frames with a dose rate of ~8.0 counts per physical pixel per second for a total exposure time of 8 s.

### Cryo-EM data processing

Original image stacks were summed and corrected for drift and beam-induced motion at the micrograph level using the MotionCor2 program^54^. The SPIDER^55^ and EMAN2 programs^56^ were used for micrograph screening and automatic particle picking, respectively. The contrast transfer function parameters of each micrograph were estimated by Gctf^57^. All 2D and 3D classification and refinement were performed with RELION2.1^58^. For the *rpf2Δ255-355* mutant sample, a total of 3189 micrographs were collected and 272,879 particles were picked for cascade 2D and 3D classification with a binning factor of 4. About 50% of particles were removed during 2 rounds of 2D classification and 143,868 particles were subjected to two rounds of 3D classification (Supplementary Fig. 5). After the final round of 3D classification, a total of 12,800 particles were subjected to high-resolution refinement (without binning), generating a density map at an overall resolution of 6.0 Å (C1 class) (gold-standard FSC 0.143 criteria). The B2 and B6 classes of the final round of 3D classification were combined for further high-resolution structural refinement, resulting in a 5.9 Å map (C2). A total of 33,582 particles from B3, B4, and B5 classes were applied for high-resolution refinement (without binning), resulting in a 3.9 Å map (C3). A total of 53,177 particles from C2 and C3 classes were applied for high-resolution refinement with a soft mask (without binning), resulting in a 3.2 Å map (C4).

For pre-ribosomes from the *GAL-SDA1* strain, a total of 1224 micrographs were collected and 169,407 particles were picked for cascade 2D and 3D classification with a binning factor of two. About 50% of particles were removed during two rounds of 2D classification and 73,883 particles were subjected to one round of 3D classification (Supplementary Fig. 7). After the final round of 3D classification, a total of 50,552 particles from the D1 and D3 classes were applied for high-resolution refinement (without binning), resulting in a 5.9 Å map (E1). A total of 14,186 particles from D2 and D4 classes were applied for high-resolution refinement (without binning), resulting in a 4.8 Å map (E2).

### C4 model building and refinement

An atomic model of the wild-type Nog2-TAP State 1 (PDB accession number 3JCT)^27^ was used as the initial template for modeling. The models of the rRNAs (25S, 5.8S, 5S, ITS2 RNA) were docked into the density map manually using UCSF Chimera^59^. For r-protein and AF modeling, structures of individual proteins were separately fitted into its density by rigid-body docking. After the initial fitting, the entire chains of rRNAs and proteins were manually checked and adjusted with COOT^60^. The atomic model of C4 of the *rpf2Δ255-344* particles was further refined against the density map first by real-space refinement (phenix.real_space_refine)^61^ in PHENIX^62^, with secondary structure restraints, geometry restraints, and RNA-specific restraints applied. After refinement, alternating rounds of manual model adjustment using COOT and model refinement using PHENIX were applied.

### High-copy suppression screen

High-copy plasmids containing the *RPF2*, *RRS1*, *NOG1*, *SDA1*, *RSA4*, or *CGR1* genes (Yeast genomic tiling collection, Open Biosystems) were transformed into the *rpf2Δ255-344* mutant strain. Serial dilutions (1 : 10 to 1 : 10,000) were spotted onto YEPGlu solid medium and incubated at 30 and 13 °C.

### Statistics and reproducibility

Experiments shown in Fig. 1a, b were performed twice. The experiment shown in Fig. 2a was performed three times with yeast expressing the genomic *rpf2Δ255-344* mutant strain, and twice with yeast expressing the plasmid-borne mutant allele. The experiment shown in Fig. 2b was performed twice with yeast expressing the genomic *rpf2Δ255-344* mutant strain, and once with yeast expressing the plasmid-borne mutant allele. The experiment shown in Fig. 2c was performed twice with yeast expressing the genomic *rpf2Δ255-344* mutant strain, and twice with yeast expressing the plasmid-borne mutant allele. The experiment shown in Supplementary Fig. 4a was performed one time, but those results were backed up by a growth defect observed in the *RPF2-TAP* strain. The experiment shown in Supplementary Fig. 4e was performed three times. Experiments shown in Supplementary Fig. 4f, g were performed twice. The experiment shown in Supplementary Fig. 5a was performed twice and an average of 150 cells were visualized to confirm the defect. Areas on the micrograph showing the highest numbers of cells were chosen for the publication. Experiments shown in Supplementary Fig. 5b, c were performed twice. Experiments shown in Supplementary Fig. 7a–c, e–g were performed twice. The experiment shown in Supplementary Fig. 7d was performed twice, and an average of 150 cells were visualized in order to confirm the defect. Areas on the micrograph showing the highest numbers of cells were chosen for the publication. The experiment shown in Supplementary Fig. 7h was performed twice.

### PyMOL images

PyMOL images of structures of 60 S subunit assembly intermediates were generated using PDB 6ELZ [10.2210/pdb6ELZ/pdb], 5T62 [10.2210/pdb5T62/pdb], 3JCT [https://www.wwpdb.org/pdb?id=pdb_00003jct], 5APN [10.2210/pdb5APN/pdb], and 5H4P [10.2210/pdb5H4P/pdb]^9,19,27,44,46^. Pymol images of the structure of yeast mature 60S ribosomal subunits were generated using PDB file 4V88 [10.2210/pdb4v88/pdb]^7^.

### Reporting summary

Further information on research design is available in the Nature Research Reporting Summary linked to this article.

## Supplementary information

Supplementary Information

Peer Review File

Reporting Summary

## Data Availability

The data that support this work are available from the corresponding authors upon reasonable request. The cryo-EM density maps of C1, C2, C3, C4 classes of *rpf2△255-344* mutant particles and E1, E2 classes of Sda1-depleted mutant particles have been deposited in the Electron Microscopy Data Bank under the following accession numbers: EMD-30110 [https://www.ebi.ac.uk/pdbe/entry/emdb/EMD-30110], EMD-30111 [https://www.ebi.ac.uk/pdbe/entry/emdb/EMD-30111], EMD-30112 [https://www.ebi.ac.uk/pdbe/entry/emdb/EMD-30112], EMD-30108 [https://www.ebi.ac.uk/pdbe/entry/emdb/EMD-30108], EMD-30113 [https://www.ebi.ac.uk/pdbe/entry/emdb/EMD-30113], and EMD-30109 [https://www.ebi.ac.uk/pdbe/entry/emdb/EMD-30109], respectively. The atomic model of the C4 class of the *rpf2△255-344* has been deposited in the Protein Data Bank (PDB) under accession number 6M62 [10.2210/pdb6M62/pdb]. Source data are provided with this paper.

## Source data

Source Data
