# Supplementary material for: Coupling of 5S RNP rotation with maturation of functional centers during large ribosomal subunit assembly
Source: Nat Commun. 2020 Jul 27;11:3751. doi: 10.1038/s41467-020-17534-5 (PMC7385084; doi:10.1038/s41467-020-17534-5)
Supplement: Supplementary file 1 — Supplementary Information [file 41467_2020_17534_MOESM1_ESM.pdf]

SUPPLEMENTARY INFORMATION FOR

Coupling of 5S RNP rotation with maturation of functional centers during large ribosomal subunit assembly

Micic et al.

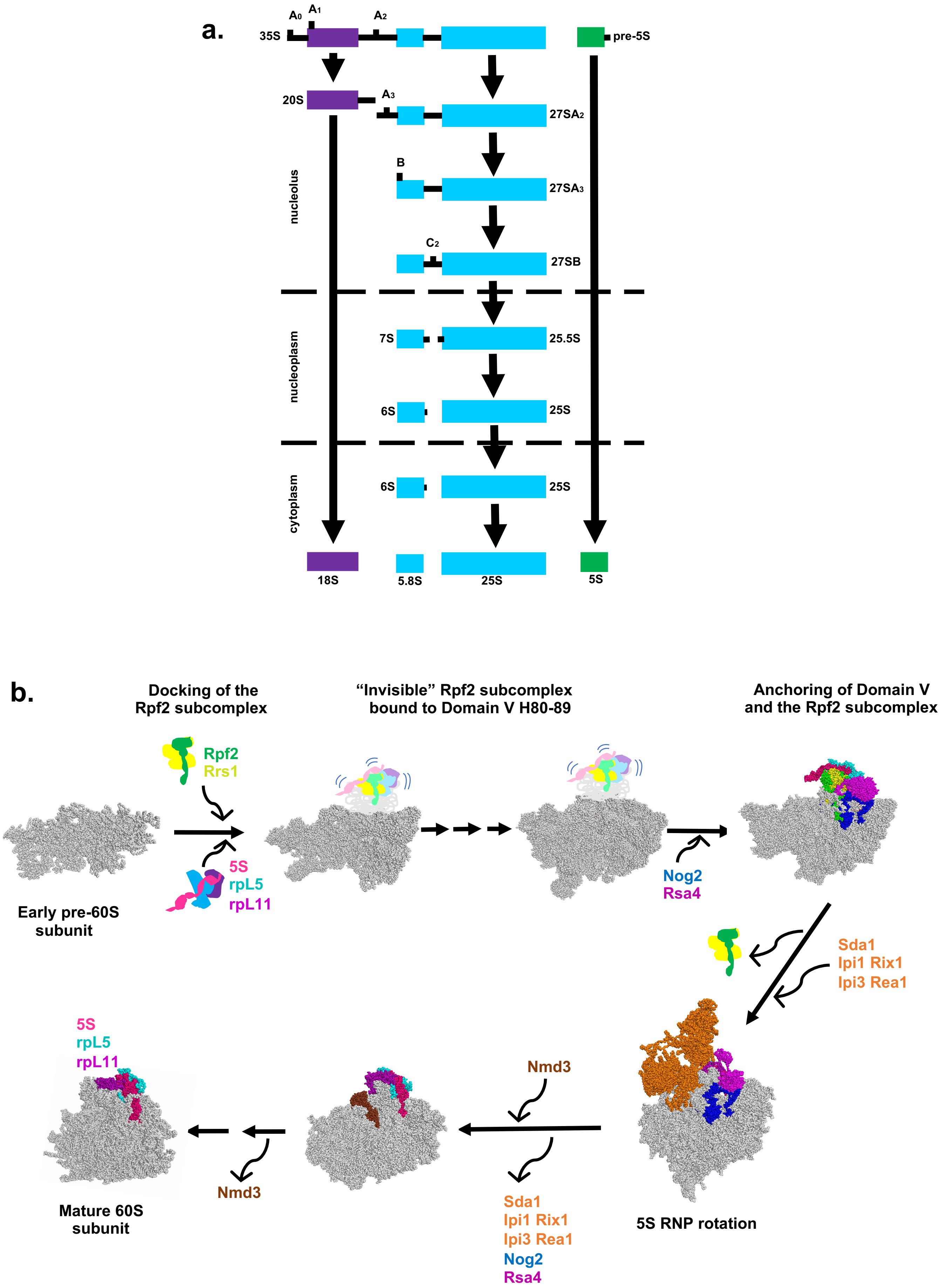
a.
A0
A1
A2
35S
pre-5S
20S
A3
27SA2
B
27SA3
C2
27SB
nucleolus
nucleoplasm
7S
25.5S
6S
25S
cytoplasm
6S
25S
18S
5.8S
25S
5S
b.
Docking of the Rpf2 subcomplex
“Invisible” Rpf2 subcomplex bound to Domain V H80-89
Anchoring of Domain V and the Rpf2 subcomplex
Rpf2
Rrs1
5S
rpL5
rpL11
Early pre-60S subunit
Nog2
Rsa4
Sda1
Ipi1 Rix1
Ipi3 Rea1
5S RNP rotation
Nmd3
Sda1
Ipi1 Rix1
Ipi3 Rea1
Nog2
Rsa4
Nmd3
5S
rpL5
rpL11
Mature 60S subunit

Supplementary Figure 1. **Middle stages in yeast 60S ribosomal subunit assembly. (a)** The simplified pre-rRNA processing pathway in *Saccharomyces cerevisiae.* 5S rRNA is transcribed and processed separately from the 35S pre-rRNA, the precursor for 25S, 18S and 5.8S rRNAs. **(b)** Docking of the 5S RNP and the Rpf2-Rrs1 heterodimer onto pre-ribosomes occurs early in biogenesis of 60S ribosomal subunits. Because these components, when they dock, are initially bound in a flexible state, they are not visible by cryo-EM until entry of AF Nog2, which associates with preribosomal particles during middle stages of subunit assembly. Upon entry of Nog2 and Rsa4, rRNA domain V and the Rpf2-subcomplex anchor stably onto preribosomes. Next, the Rpf2/Rrs1 dimer exits from assembling subunits, in order for Sda1, Ipi1, Rix1, Ipi3, and Rea1 to stably bind, and for rotation of 5S RNP to occur. Then, Rsa4 is released by Rea1, along with Ipi1, Rix1, Ipi3 and Rea1 itself. Subsequently, Nog2 exits the particles, to be replaced by the export factor Nmd3. Structures depicted here are from refs. 9 (State E), 27 (Nog2 particle), 44 (Arx1 particle), 49 (Nmd3-particle), and 7 (mature 60S subunit).

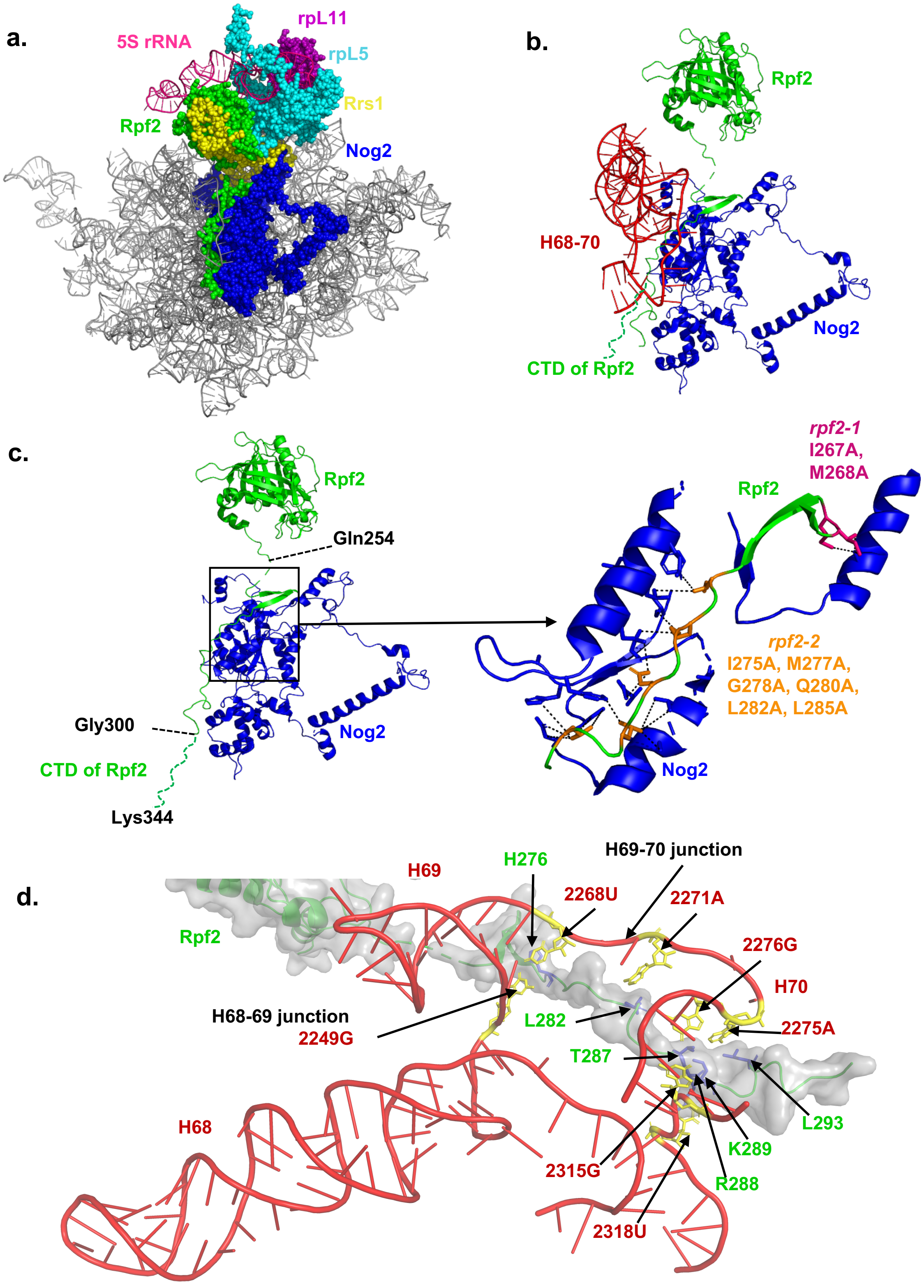

Supplementary Figure 2. **The CTD of Rpf2 is in proximity to Nog2 and rRNA helices H68-70. (a)** The Rpf2 subcomplex (Rpf2, Rrs1, rpL5, rpL11, and 5S rRNA) and Nog2 are shown (PDB ID 3JCT [https://www.wwpdb.org/pdb?id=pdb_00003jct]). **(b)** The CTD of Rpf2 is positioned between the GTPase domain of Nog2 and rRNA helices H68-H70. **(c)** Interaction

between the CTD of Rpf2 and the GTPase domain of Nog2. Amino acids 301-344 are not visualized by cryo-EM, presumably due to their mobility, and are represented by a dashed line. Clusters of amino acids that were mutated (*rpf2-1,* shown in magenta, and *rpf2-2,* orange) are located near the GTPase domain of Nog2. For clarity, only relevant side chains are shown. The last amino acid before the truncation, Gln 254, is labeled. **(d)** The CTD of Rpf2 is in close proximity to junctions between H68 and H69, and H69 and H70, respectively. Atomic models of the Rpf2-CTD and H68-70 (PDB ID 3JCT [https://www.wwpdb.org/pdb?id=pdb_00003jct]) are shown with interacting residues colored and marked. For clarity, only relevant side chains are shown.

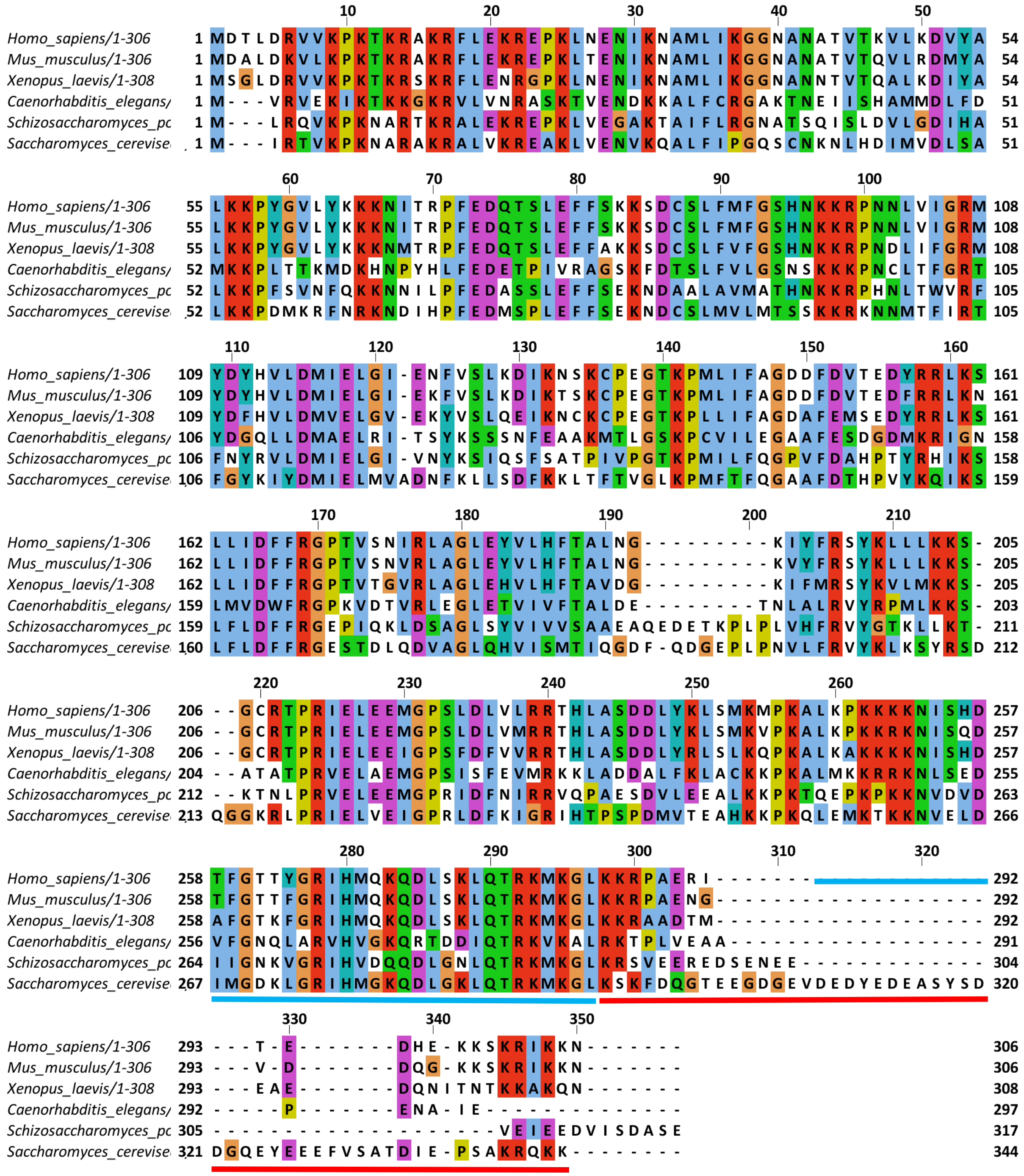

Supplementary Figure 3. **The CTD of Rpf2 includes highly conserved amino acids and a disordered domain.** The multiple sequence alignment of the Rpf2 protein from six different organisms (labeled on the left side of the figure) was obtained using Jalview 2.11.0. Portions of the C-terminal extension of Rpf2 are highly conserved (underlined in blue), while the disordered part of the extension is not conserved (underlined in red).

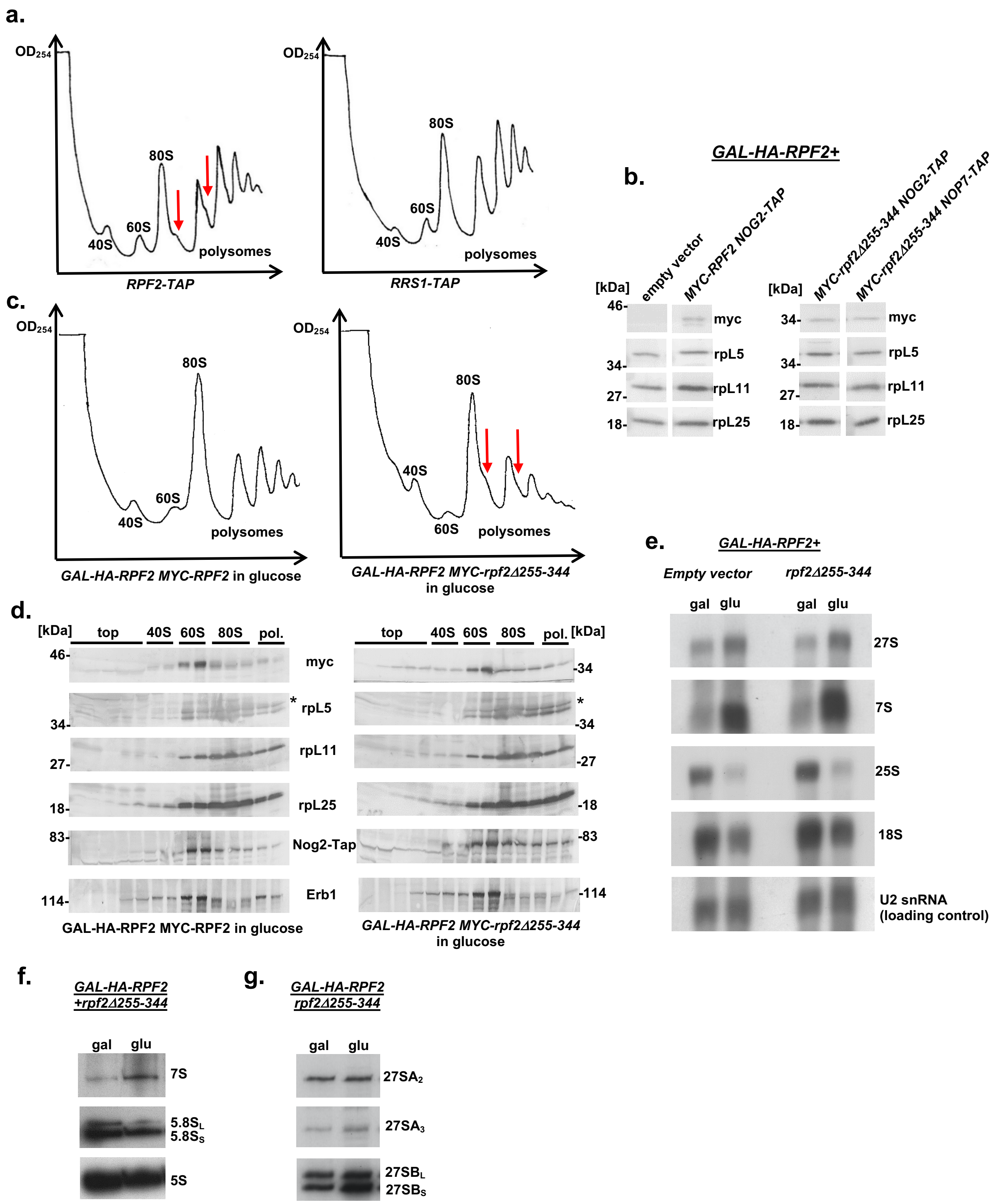

Supplementary Figure 4. **Rpf2 lacking the CTD is stably expressed, and causes a block in middle stages of 60S ribosomal subunit assembly. (a)** Epitope-tagging of Rpf2 at its C-terminus affects the levels of 60S ribosomal subunits. The Rrs1-Tap strain was used as a control. Arrows represent halfmer polysomes (polysomes containing one 40S subunit bound to the AUG start site in mRNA without a partner 60S subunit). **(b)** Western blots of whole-cell lysates from strains expressing either wild-type or truncated Myc-tagged Rpf2 proteins. RpL25 was used as a

control. All samples were derived from the same experiment, and western blots were processed in parallel. Molecular weight standards are labeled**. (c)** Levels of free 60S subunits in the Myc-*rpf2Δ255-344* strain are significantly lower relative to 40S subunits, compared to the wild-type Myc-Rpf2 strain. Arrows represent halfmer polysomes. A slight decrease in levels of 60S subunits in the Myc-Rpf2 control strain is probably due to a slight deficiency in levels of Myc-Rpf2 expressed from a plasmid. **(d)** The sedimentation pattern on sucrose gradients for Myc-Rpf2, Myc-rpf2Δ255-344, rpL5, rpL11, rpL25, Nog2-Tap and Erb1 in the strains expressing wild-type Rpf2 or truncated rpf2 protein. A band that cross-reacts with the anti-rpL5 antibody is indicated by an asterisk. All samples were derived from the same experiment, and western blots were processed in parallel. Molecular weight standards are labeled. **(e)** Northern hybridization in cells depleted of Rpf2 or expressing the truncated rpf2 protein shows that the pre-rRNA processing defects are identical. U2 snRNA was used as a loading control. **(f)** The same as **(e),** except that an acrylamide gel was used to separate smaller RNA species. Levels of 7S pre-rRNA increase, consistent with the results in **(e)**, and levels of mature 5.8S rRNA decrease. As expected, amounts of 5S rRNA are not significantly affected. 7S pre-RNA species are much less abundant than mature rRNAs, explaining the observed difference in intensity of signals. **(g)** Primer extension assays indicate that processing of 27SB pre-rRNA is affected upon depletion of Rpf2, as well as when the CTD of Rpf2 is truncated.

Source data are provided as a Source Data file.

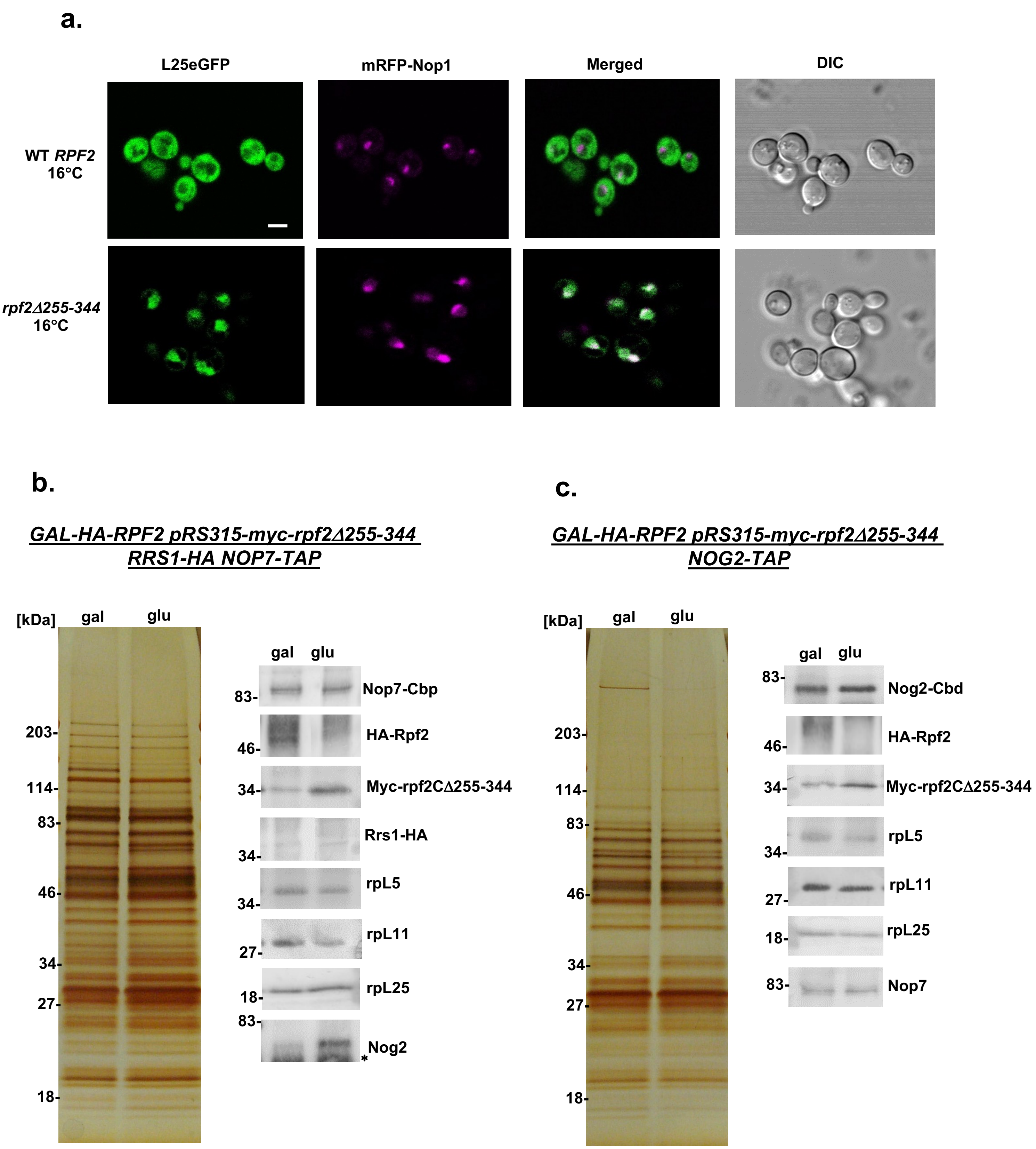
a.
L25eGFP
mRFP-Nop1
Merged
DIC
WT RPF2
16°C
rpf2Δ255-344
16°C
b.
GAL-HA-RPF2 pRS315-myc-rpf2Δ255-344
RRS1-HA NOP7-TAP
[kDa]
gal
glu
203-
114-
83-
46-
34-
27-
18-
Nop7-Cbp
HA-Rpf2
Myc-rpf2CΔ255-344
Rrs1-HA
rpL5
rpL11
rpL25
Nog2
*
c.
GAL-HA-RPF2 pRS315-myc-rpf2Δ255-344
NOG2-TAP
Nog2-Cbd
Nop7

Supplementary Figure 5. **Truncated Rpf2 protein expressed from a plasmid is stable, and can enter assembling 60S ribosomal subunits.** **(a)** Pre-60S ribosomes accumulate mostly in the nucleoplasm in cells expressing the truncated Rpf2 protein. The L25-eGFP reporter for preribosomes and ribosomes (green), the nucleolar marker Nop1-mRFP (magenta), the merged signal, and the phase contrast images (DIC) of cells are labeled. Scale bars (marked in white) are 5 µm. **(b)** SDS-PAGE gels and western blots of proteins in pre-ribosomes affinity-purified (using Nop7-TAP) from the *GAL-HA-RPF2 RRS1-HA NOP7-TAP pRS315-2MYC-rpf2Δ255-344* mutant strain. These results confirm that the truncated rpf2 protein, as well as the other constituents of the Rpf2 subcomplex, can enter pre-ribosomal 60S particles. In addition, levels of Nog2 increase, presumably due to the block in assembly during the lifetime of Nog2 particles. The asterisk below Nog2 represents IgG. Nop7-Cbp (calmodulin binding peptide left behind after TEV cleavage during elution removes the Protein A moiety of the TAP tag) was used as loading control. All samples were derived from the same experiment, and western blots were processed in parallel. Molecular weight standards are labeled. **(c)** SDS-PAGE gels and western blots of pre-ribosomal proteins from Nog2-TAP affinity purifications, using the *GAL-HA-RPF2 NOG2-TAP pRS315-2MYC-rpf2Δ255-344* mutant strain. Truncated rpf2 protein, as well as the other constituents of the Rpf2 subcomplex, can enter preribosomal 60S particles. Nog2-Cbp (calmodulin binding peptide left behind after TEV cleavage during elution removes the Protein A moiety of the TAP tag) was used as a loading control. All samples were derived from the same experiment, and western blots were processed in parallel. Molecular weight standards are labeled.

Source data are provided as a Source Data file.

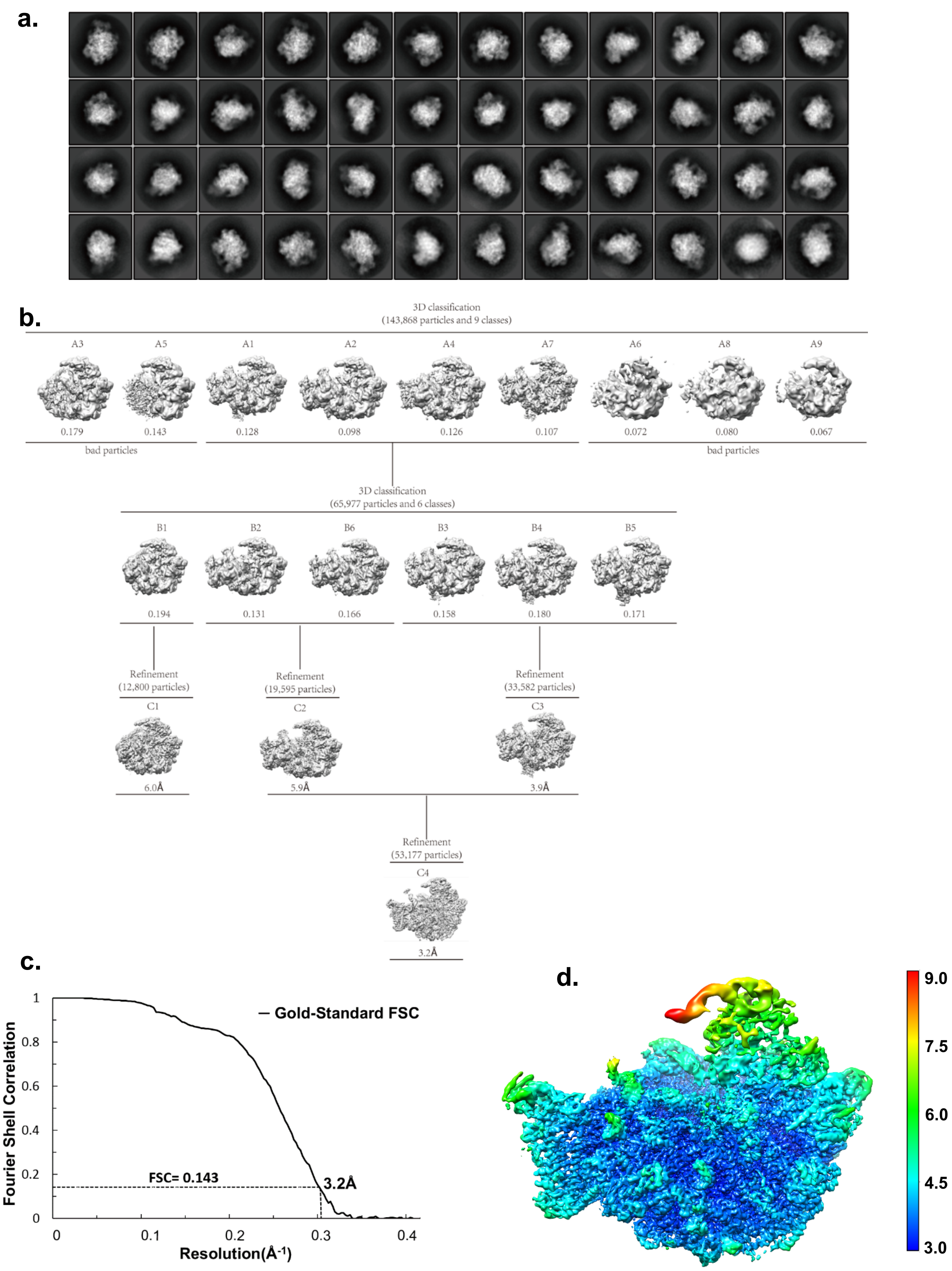
a.
b.
3D classification
(143,868 particles and 9 classes)
A3
A5
A1
A2
A4
A7
A6
A8
A9
0.179
0.143
0.128
0.098
0.126
0.107
0.072
0.080
0.067
bad particles
bad particles
3D classification
(65,977 particles and 6 classes)
B1
B2
B6
B3
B4
B5
0.194
0.131
0.166
0.158
0.180
0.171
Refinement
(12,800 particles)
Refinement
(19,595 particles)
Refinement
(33,582 particles)
C1
C2
C3
6.0Å
5.9Å
3.9Å
Refinement
(53,177 particles)
C4
3.2Å
c.
— Gold-Standard FSC
Fourier Shell Correlation
1
0.8
0.6
0.4
0.2
0
FSC= 0.143
3.2Å
0
0.1
0.2
0.3
0.4
Resolution(Å-1)
d.
9.0
7.5
6.0
4.5
3.0

Supplementary Figure 6. **Summary of cryo-EM data processing of Nog2 particles from the C4 class the *rpf2Δ255-344* mutant strain.** **(a)** Representative 2D class averages of cryo-EM particles. **(b)** A flow-chart for 3D classification of cryo-EM particles. **(c)** FSC (Fourier shell correlation) curve for the C4 class. **(d)** Local resolution map for the C4 class.

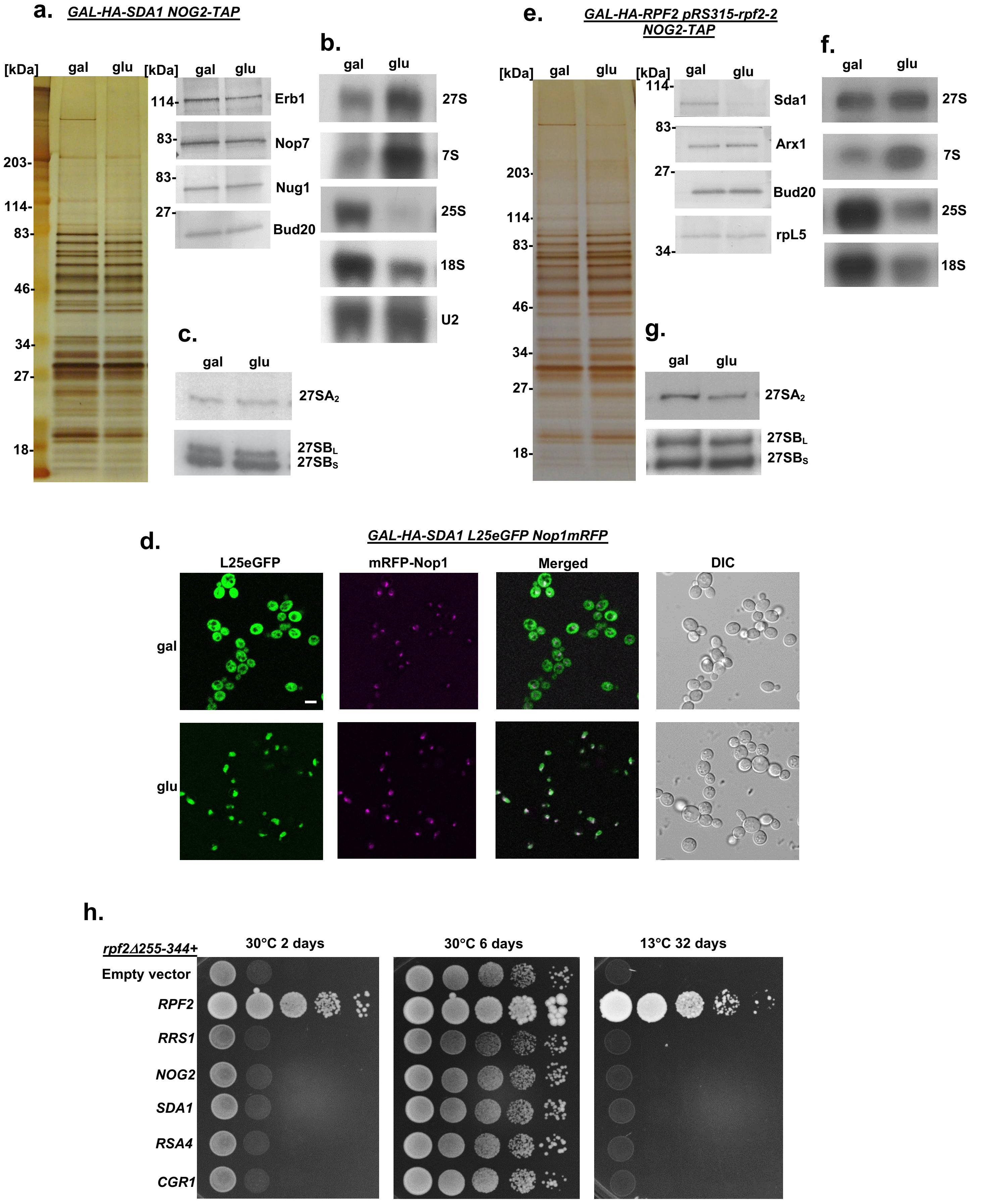

a.
GAL-HA-SDA1 NOG2-TAP
[kDa]
gal
glu
203-
114-
83-
46-
34-
27-
18-
[kDa]
114-
83-
83-
27-
Erb1
Nop7
Nug1
Bud20
b.
gal
glu
27S
7S
25S
18S
U2
c.
gal
glu
27SA2
27SBL
27SBS
e.
GAL-HA-RPF2 pRS315-rpf2-2
NOG2-TAP
[kDa]
gal
glu
203-
114-
83-
46-
34-
27-
18-
[kDa]
114-
83-
27-
34-
Sda1
Arx1
Bud20
rpL5
f.
gal
glu
27S
7S
25S
18S
g.
gal
glu
27SA2
27SBL
27SBS
d.
GAL-HA-SDA1 L25eGFP Nop1mRFP
L25eGFP
mRFP-Nop1
Merged
DIC
gal
glu
h.
rpf2Δ255-344+
30°C 2 days
30°C 6 days
13°C 32 days
Empty vector
RPF2
RRS1
NOG2
SDA1
RSA4
CGR1

Supplementary Figure 7. **Defects in ribosome biogenesis upon depletion of Sda1 or mutation of conserved amino acids in the CTD of Rpf2**. **(a)** SDS-PAGE and western blots of proteins in assembling 60S subunits from cells depleted of Sda1. Preribosomal particles were purified using the Nog2 as a bait, and protein constituents were separated by SDS-PAGE and stained with silver. Levels of Bud20 do not change based on western blotting, but portions of Bud20 in pre-ribosomes are invisible by cryo-EM, presumably because they are flexible (Figure 9, bottom panel). All samples were derived from the same experiment, and western blots were processed in parallel. Molecular weight standards are labeled. **(b)** Northern hybridization from cells depleted of Sda1 or expressing truncated rpf2 protein. U2 snRNA was used as a loading control. **(c)** Primer extension assays of pre-rRNA processing upon depletion of Sda1. A block in 27SB pre-rRNA processing is not as strong as upon truncation of the CTD of Rpf2 (Supplementary Fig. 4g). **(d)** Cells depleted of Sda1 protein accumulate pre-60S subunits mostly in the nucleoplasm. The L25-eGFP pre-ribosome/ribosome reporter (green), the nucleolar marker Nop1-mRFP (magenta), the merged signal, and phase contrast images (DIC) of the cells are labeled. Scale bars (marked in white) are 5 µm. **(e)** SDS-PAGE and western blotting of proteins in assembling 60S subunits from cells expressing rpf2-2 mutant protein. Pre-ribosomal particles were purified using the Nog2 as a bait, and protein constituents were separated by SDS-PAGE and stained with silver. All samples were derived from the same experiment, and western blots were processed in parallel. Molecular weight standards are labeled. **(f)** Northern hybridization indicates that only 7S pre-rRNA accumulates upon expression of the mutant rpf2-2 protein. **(g)** Primer extension assays demonstrate that the early pre-rRNA processing intermediates $27SA_2$, $27SA_3$, and 27SB do not accumulate in the rpf2-2 mutant. **(h)** Overexpression of Sda1 fails to suppress the cold-sensitive phenotype of the *rpf2Δ255-344* mutant. High copy plasmids expressing Rpf2, Rrs1, Nog2, Sda1, Rsa4, or Cgr1, were expressed the *rpf2Δ255-344* mutant strain and incubated at 30 or 13°C.

Source data are provided as a Source Data file.

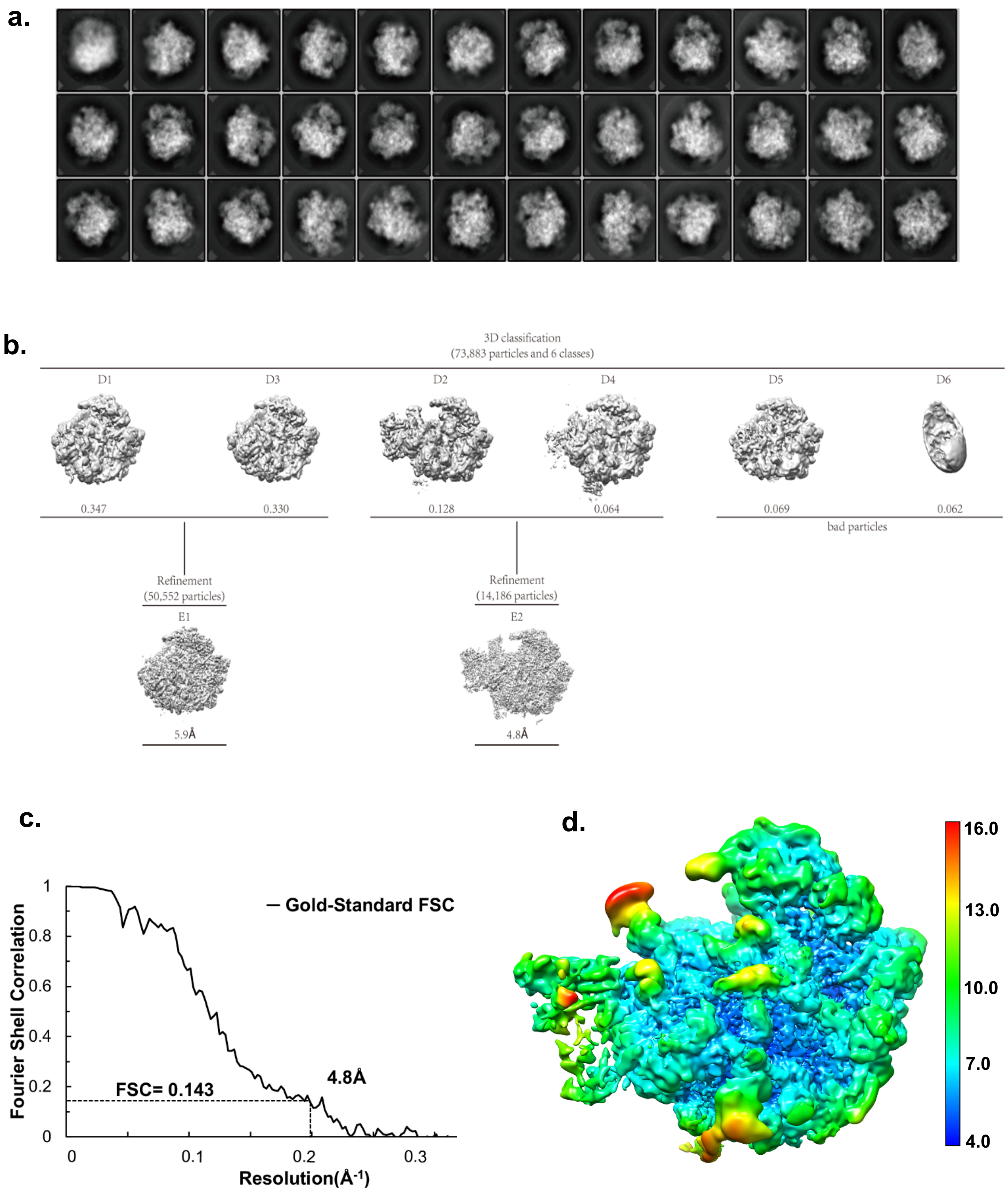

Supplementary Figure 8. **Summary of cryo-EM data processing of Nog2 particles from the E2 class of the Sda1-depleted strain. (a)** Representative 2D class averages of cryo-EM particles. **(b)** A flow-chart for 3D classification of cryo-EM particles. **(c)** FSC (Fourier shell correlation) curve for the E2 class. **(b)** Local resolution map for the E2 class.

Strain	Genotype	Source
JWY8178	*Matα trp1 lys2-801 ura3-52 his3Δ200 leu2-1 rpf2::GAL-HA3-RPF2 TRP1 nop7::NOP7-TAP URA3 rrs1::RRS1-HA3 HIS3*	Zhang et al, 2007
JWY11279	*Matα trp1 lys2-801 ura3-52 his3Δ200 leu2-1 rpf2::GAL-HA3-RPF2 TRP1 nop7::NOP7-TAP URA3 rrs1::RRS1-HA3 HIS3 + pRS315-rpf2Δ255-344 CEN Amp^r LEU2*	This study
JWY11282	*Matα trp1 lys2-801 ura3-52 his3Δ200 leu2-1 rpf2::GAL-HA3-RPF2 TRP1 nop7::NOP7-TAP URA3 rrs1::RRS1-HA3 HIS3 + pRS315 CEN Amp^r LEU2*	This study
JWY11283	*Matα trp1 lys2-801 ura3-52 his3Δ200 leu2-1 rpf2::GAL-HA3-RPF2 TRP1 nop7::NOP7-TAP URA3 rrs1::RRS1-HA3 HIS3 + pRS315-RPF2 CEN Amp^r LEU2*	This study
JWY 8680	*Matα trp1 lys2-801 ura3-52 his3Δ200 leu2-1 rpf2::GAL-HA3-RPF2 TRP1 nog2::NOG2-TAP URA3*	This study
JWY11295	*Matα trp1 lys2-801 ura3-52 his3Δ200 leu2-1 rpf2::GAL-HA3-RPF2 TRP1 nog2::NOG2-TAP URA3 + pRS315 CEN Amp^r LEU2*	This study
JWY11296	*Matα trp1 lys2-801 ura3-52 his3Δ200 leu2-1 rpf2::GAL-HA3-RPF2 TRP1 nog2::NOG2-TAP URA3 + pRS315-RPF2 CEN Amp^r LEU2*	This study
JWY11297	*Matα trp1 lys2-801 ura3-52 his3Δ200 leu2-1 rpf2::GAL-HA3-RPF2 TRP1 nog2::NOG2-TAP URA3 + pRS315-rpf2Δ255-344 CEN Amp^r LEU2*	This study
JWY11509	*Mat a/α ura3-1/ura3-1 trp1-1/trp1-1 leu2-3,112/leu2-3,112 his3-11,15/his3-11,15 ade2-1/ade2-1 can1-100/can1-100 RPF2/rpf2Δ255-344 G418^r*	This study
JWY11525	Spore #1 from dissection of JWY11509 *ura3-1 trp1-1 leu2-3,112 his3-11,15 ade2-1 can1-100 rpf2Δ255-344 G418^r*	This study
JWY11528	Spore #2 from dissection of JWY11509 *ura3-1 trp1-1 leu2-3,112 his3-11,15 ade2-1 can1-100 RPF2*	This study
JWY11535	*ura3-1 trp1-1 leu2-3,112 his3-11,15 ade2-1 can1-100 rpf2Δ255-344 G418^r + p3316 (pURA3 mRFP-NOP1 RPL25-eGFP)*	This study
JWY11539	*ura3-1 trp1-1 leu2-3,112 his3-11,15 ade2-1 can1-100 rpf2Δ255-344 G418^r nog2::NOG2-TAP URA3*	This study
JWY11542	*Matα trp1 lys2-801 ura3-52 his3Δ200 leu2-1 rpf2::GAL-HA3-RPF2 TRP1 nog2::NOG2-TAP URA3 + pRS315-rpf2Δ310-344 CEN Amp^r LEU2*	This study

JWY11544	*Matα trp1 lys2-801 ura3-52 his3Δ200 leu2-1 rpf2::GAL-HA3-RPF2 TRP1 nog2::NOG2-TAP URA3 + pRS315-rpf2-1 CEN Amp^r LEU2*	This study
JWY11546	*Matα trp1 lys2-801 ura3-52 his3Δ200 leu2-1 rpf2::GAL-HA3-RPF2 TRP1 nog2::NOG2-TAP URA3 + pRS315-rpf2-2 CEN Amp^r LEU2*	This study
JWY11635	*ura3-1 trp1-1 leu2-3,112 his3-11,15 ade2-1 can1-100 rpf2Δ255-344 G418^r + pRS315 CEN Amp^r LEU2*	This study
JWY11636	*ura3-1 trp1-1 leu2-3,112 his3-11,15 ade2-1 can1-100 rpf2Δ255-344 G418^r + pGP564-RPF2*	This study
JWY11638	*ura3-1 trp1-1 leu2-3,112 his3-11,15 ade2-1 can1-100 rpf2Δ255-344 G418^r + pGP564-RRS1*	This study
JWY11640	*ura3-1 trp1-1 leu2-3,112 his3-11,15 ade2-1 can1-100 rpf2Δ255-344 G418^r + pGP564-NOG2*	This study
JWY11642	*ura3-1 trp1-1 leu2-3,112 his3-11,15 ade2-1 can1-100 rpf2Δ255-344 G418^r + pGP564-SDA1*	This study
JWY11644	*ura3-1 trp1-1 leu2-3,112 his3-11,15 ade2-1 can1-100 rpf2Δ255-344 G418^r + pGP564-RSA4*	This study
JWY11646	*ura3-1 trp1-1 leu2-3,112 his3-11, ade2-1 can100 rpf2Δ255-344 G418^r + pGP564-CGR1*	This study
JWY11647	*Matα trp1 lys2-801 ura3-52 his3Δ200 leu2-1 rpf2::GAL-HA3-RPF2 TRP1 nop7::NOP7-TAP URA3 rrs1::RRS1-HA3 HIS3 + pRS315-2Myc-rpf2Δ255-344 CEN Amp^r LEU2*	This study
JWY11648	*Matα trp1 lys2-801 ura3-52 his3Δ200 leu2-1 rpf2::GAL-HA3-RPF2 TRP1 nop7::NOP7-TAP URA3 rrs1::RRS1-HA3 HIS3 + pRS315-2Myc-RPF2 CEN Amp^r LEU2*	This study
JWY11649	*Matα trp1 lys2-801 ura3-52 his3Δ200 leu2-1 rpf2::GAL-HA3-RPF2 TRP1 nog2::NOG2-TAP URA3 + pRS315-2Myc-rpf2Δ255-344 CEN Amp^r LEU2*	This study
JWY11650	*Matα trp1 lys2-801 ura3-52 his3Δ200 leu2-1 rpf2::GAL-HA3-RPF2 TRP1 nog2::NOG2-TAP URA3 + pRS315-2Myc-RPF2 CEN Amp^r LEU2*	This study
JWY10904	*ura3-1 trp1-1 leu2-3,112 his3-11,15 ade2-1 can1-100 sda1::GAL-SDA1*	Gift from Dr. Doug Kellogg (ZZ86)
JWY10917	*ura3-1 trp1-1 leu2-3,112 his3-11,15 ade2-1 can1-100 sda1::GAL-SDA1 nog2::NOG2-TAP URA3*	This study

Supplementary Table 1. **Yeast strains used in this study.**

Plasmid name	Description	Source
pRS315	Cloning vector with multiple cloning sites (MCS), *CEN Amp^r LEU2*	Sikorski, R.S. and Hieter, P. Genetics (1988)
pRS315-*RPF2*	*RFP2* ORF and approximately 700 nucleotides upstream from the start codon were cloned into MCS of pRS315 using restriction enzymes BamHI and SacII	This study
pRS315-*rpf2Δ255-344*	pRS315-*RPF2* was mutagenized using the Agilent QuikChange mutagenesis kit. Amino acids 255-344 are deleted from the C-terminal part of the Rpf2	This study
p3316	*pURA3 mRFP-NOP1 RPL25-eGFP CEN Amp^r LEU2*	Dr. Jochem Baßler
pRS315-*rpf2Δ310-344*	pRS315-*RPF2* was mutagenized using the Agilent QuikChange mutagenesis kit. Amino acids 310-344 are deleted from the C-terminal part of the Rpf2	This study
pRS315-*rpf2-1*	pRS315-*RPF2* was mutagenized using the Agilent QuikChange mutagenesis kit. The following substitutions were introduced: I267A and M268A	This study
pRS315-*rpf2-2*	pRS315-*RPF2* was mutagenized using the Agilent QuikChange mutagenesis kit. The following substitutions were introduced: I275A, M277A, G278A, Q280A, L282A, L285A	This study
pGP564-*RPF2*	Plasmid from tiling library YGPM29n02 containing *[TRZ1], MTD1,* ***RPF2****, NUP133, DAD2, HBS1, MRPC20, [PRP16]*	Open Biosystems (Horizon Discovery)
pGP564-*RRS1*	Plasmid from tiling library YGPM17d04 containing *[RPS10A], YOR293-A,* ***RRS1****, UAF30, YOR296W, TIM18, YOR298W, MCF1*	Open Biosystems (Horizon Discovery)
pGP564-*NOG2*	Plasmid from tiling library YGPM18f18 containing *[MSO1], LYS9, snR49, BRE5, POP2,* ***NOG2****, snR191, [ESF2]*	Open Biosystems (Horizon Discovery)
pGP564-*SDA1*	Plasmid from tiling library YGPM2k06 containing *[PFK1], YGR2406-A, YAP1802, YGR242W, FMP43, LSG2,* ***SDA1****, BRF1, CPO1*	Open Biosystems (Horizon Discovery)
pGP564-*RSA4*	Plasmid from tiling library YGPM23o21 containing *[CPR4], IMG2,* ***RSA4****, SSK22, SOL2, ERS1, YCR075W-A, [YCR076C]*	Open Biosystems (Horizon Discovery)
pGP564-*CGR1*	Plasmid from tiling library YGPM25d16 containing *HOP2, AGA2, RPC24A, RPL30,* ***CGR1****, tf(GAA)G, SCW11, CWH41, TRP5, [PGO1], [SST3]*	Open Biosystems (Horizon Discovery)
pRS315-*2MYC-RPF2*	Sequences for 2 Myc epitope tag were introduced at the N-terminus of *RPF2* in the pRS315-*RPF2*	Epoch Life Science Inc.
pRS315-*2MYC-rpf2Δ255-344*	Sequences for 2 Myc epitope tag were introduced at the N-terminus of *rpf2* in the pRS315-*rpf2Δ255-344*	Epoch Life Science Inc.

Supplementary Table 2. **Plasmids used in this study.**

	#1 C4 (EMDB-30108) (PDB 6M62)	#2 C1 (EMDB-30110)	#3 C2 (EMDB-30111)	#4 C3 (EMDB-30112)	#5 E1 (EMDB-30113)	#6 E2 (EMDB-30109)
**Data collection and processing**						
Magnification	130,000x	130,000x	130,000x	130,000x	130,000x	130,000x
Voltage (kV)	300	300	300	300	300	300
Electron exposure (e–/Å^2)	7.2	7.2	7.2	7.2	7.2	7.2
Defocus range (μm)	-1.0 ~ -2.0	-1.0 ~ -2.0	-1.0 ~ -2.0	-1.0 ~ -2.0	-1.0 ~ -2.0	-1.0 ~ -2.0
Pixel size (Å)						
Symmetry imposed	C1	C1	C1	C1	C1	C1
Initial particle images (no.)	143,868	143,868	143,868	143,868	73,883	73,883
Final particle images (no.)	53,177	12,800	19,595	33,582	50,552	14,186
Map resolution (Å)	3.2	6.0	5.9	3.9	5.9	4.8
FSC threshold	0.143	0.143	0.143	0.143	0.143	0.143
Map resolution range (Å)	3.0-9.0	5.0-20.0	5.0-20.0	3.3-10.0	5.0-20.0	4.0-16.0
**Refinement**						
Initial model used (PDB code)	3jct					
Model resolution (Å)	3.2					
FSC threshold	0.143					
Model resolution range (Å)	3.0-9.0					
Map sharpening *B* factor (Å^2)	-91.9					
Model composition						

Non-hydrogen atoms	154,259
Protein residues	10,445
Ligands	8
B factors (Å^2)	
Protein	94.52
Ligand	98.99
R.m.s. deviations	
Bond lengths (Å)	0.010 (25)
Bond angles (°)	1.126 (176)
Validation	
MolProbity score	2.02
Clashscore	7.15
Poor rotamers (%)	1.10
Ramachandran plot	
Favored (%)	88.44
Allowed (%)	11.22
Disallowed (%)	0.34

Supplementary Table 3. **Cryo-EM data collection, refinement and validation statistics**
