# Supplementary material for: Coupling of 5S RNP rotation with maturation of functional centers during large ribosomal subunit assembly
Source: Nat Commun. 2020 Jul 27;11:3751. doi: 10.1038/s41467-020-17534-5 (PMC7385084; doi:10.1038/s41467-020-17534-5)
Supplement: Supplementary file 3 — Reporting Summary [file 41467_2020_17534_MOESM3_ESM.pdf]

nature research

Corresponding author(s): Ning Gao, John L Woolford, Jr.

Last updated by author(s): 06/25/2020

# Reporting Summary

Nature Research wishes to improve the reproducibility of the work that we publish. This form provides structure for consistency and transparency in reporting. For further information on Nature Research policies, see Authors & Referees and the Editorial Policy Checklist .

## Statistics

For all statistical analyses, confirm that the following items are present in the figure legend, table legend, main text, or Methods section.

| n/a | Confirmed | |
|---|---|---|
| ☐ | ☒ | The exact sample size (*n*) for each experimental group/condition, given as a discrete number and unit of measurement |
| ☐ | ☒ | A statement on whether measurements were taken from distinct samples or whether the same sample was measured repeatedly |
| ☒ | ☐ | The statistical test(s) used AND whether they are one- or two-sided<br>*Only common tests should be described solely by name; describe more complex techniques in the Methods section.* |
| ☒ | ☐ | A description of all covariates tested |
| ☒ | ☐ | A description of any assumptions or corrections, such as tests of normality and adjustment for multiple comparisons |
| ☐ | ☒ | A full description of the statistical parameters including central tendency (e.g. means) or other basic estimates (e.g. regression coefficient) AND variation (e.g. standard deviation) or associated estimates of uncertainty (e.g. confidence intervals) |
| ☒ | ☐ | For null hypothesis testing, the test statistic (e.g. *F*, *t*, *r*) with confidence intervals, effect sizes, degrees of freedom and *P* value noted<br>*Give P values as exact values whenever suitable.* |
| ☒ | ☐ | For Bayesian analysis, information on the choice of priors and Markov chain Monte Carlo settings |
| ☒ | ☐ | For hierarchical and complex designs, identification of the appropriate level for tests and full reporting of outcomes |
| ☒ | ☐ | Estimates of effect sizes (e.g. Cohen's *d*, Pearson's *r*), indicating how they were calculated |

*Our web collection on statistics for biologists contains articles on many of the points above.*

## Software and code

Policy information about availability of computer code

| | |
|---|---|
| Data collection | Cryo-EM data were recorded using the software Serial EM v3.6, and raw particles were randomly selected using the computer program RELION 2.1; Images for fluorescence microscopy were acquired using ZEN 2 software, blue edition (Zeiss) |
| Data analysis | Cryo-EM data were processed using MotionCor2 v1.1.0, Gctf v1.06, Chimera v1.11.2, Pymol v2.x, Coot v0.8.7 and Phenix v1.18.2; Protein Pilot 5.0 was used to obtain iTRAQ ratios as an average of all peptides for each protein; Images for fluorescence microscopy were processed using Fiji for Mac OSX (National Institutes of Health); Jalview 2.11.1.0. was used for multiple sequence alignment visualization and analysis |

For manuscripts utilizing custom algorithms or software that are central to the research but not yet described in published literature, software must be made available to editors/reviewers. We strongly encourage code deposition in a community repository (e.g. GitHub). See the Nature Research guidelines for submitting code & software for further information.

## Data

Policy information about availability of data

All manuscripts must include a data availability statement. This statement should provide the following information, where applicable:

- Accession codes, unique identifiers, or web links for publicly available datasets
- A list of figures that have associated raw data
- A description of any restrictions on data availability

The cryo-EM density maps of C1, C2, C3, C4 classes of rpf2△255-344 mutant particles, and E1, E2 classes of Sda1-depleted mutant particles have been deposited in the Electron Microscopy Data Bank [http://www.emdataresource.org/] under accession numbers: EMD-30110 (C1) [https://www.ebi.ac.uk/pdbe/entry/emdb/EMD-30110], EMD-30111 (C2) [https://www.ebi.ac.uk/pdbe/entry/emdb/EMD-30111], EMD-30112 (C3) [https://www.ebi.ac.uk/pdbe/entry/emdb/EMD-30112], EMD-30108 (C4) [https://www.ebi.ac.uk/pdbe/entry/emdb/EMD-30108], EMD-30113 (E1) [https://www.ebi.ac.uk/pdbe/entry/emdb/EMD-30113], and EMD-30109 (E2) [https://www.ebi.ac.uk/pdbe/entry/emdb/EMD-30109], respectively. The atomic model of the C4 class of the rpf2△255-344 has been deposited in the Protein Data Bank (PDB) [https://www.rcsb.org/] under accession number 6M62 [http://dx.doi.org/10.2210/pdb6M62/pdb].

Yeast strains and plasmids used in this study are available upon request. Sequences of oligonucleotides used for strain constructions and mutagenesis are available upon request. Source data are provided with this paper.

# Field-specific reporting

Please select the one below that is the best fit for your research. If you are not sure, read the appropriate sections before making your selection.

☒ Life sciences ☐ Behavioural & social sciences ☐ Ecological, evolutionary & environmental sciences

For a reference copy of the document with all sections, see nature.com/documents/nr-reporting-summary-flat.pdf

# Life sciences study design

All studies must disclose on these points even when the disclosure is negative.

| | |
|---|---|
| Sample size | For the cryo-EM data, we collected >3,000 raw movie micrographs using Titan Krios electron microscope. We chose 272,879 raw particles. After 2D classification, a total of 143,868 "good" particles that produced clear 2D averages were retained in the dataset. After 3D classification, 53,177 raw particles were applied for high-resolution refinement resulted in the final 3D map at 3.2 Å resolution. The other sample reached 4.8 Å resolution. The sample size was deemed sufficient because the data yielded our targeted resolution. |
| Data exclusions | Regarding the cryo-EM raw micrograph screening, exclusions were done based on the quality of the images and the presence of ice contamination. Regarding the particle selection, 2D and 3D classification were used, and criterion was based on the quality of resulting 2D class average and 3D maps. This criteria is empirical, but is a standard image processing practice in the cryo-EM community. |
| Replication | For the cryo-EM data, the reproducibility lies in a large number of particles used to derive final 3D maps. Reliability and resolution are measured by gold-standard Fourier shell correlation (Supplementary Figures 6 and 8). The replication efforts through multiple refinement runs were successful and yielded similar 3D maps.<br>For all other experiments, there is a section in the Methods, titled “Statistics and Reproducibility”, stating the number of times each experiment was performed for relevant figures. All our duplicated experiments are biological replicates, not technical replicates. |
| Randomization | The raw particles were randomly selected by a computer program (RELION 2.1). Cry-EM reconstructions use two randomized half-sets to prevent over-refinement of the model, and to assess the resolution of the final model. |
| Blinding | Investigators were not blinded during grouping, since it is performed computationally. |

# Reporting for specific materials, systems and methods

We require information from authors about some types of materials, experimental systems and methods used in many studies. Here, indicate whether each material, system or method listed is relevant to your study. If you are not sure if a list item applies to your research, read the appropriate section before selecting a response.

## Materials & experimental systems

| n/a | Involved in the study | |
|---|---|---|
| ☐ | ☒ | Antibodies |
| ☒ | ☐ | Eukaryotic cell lines |
| ☒ | ☐ | Palaeontology |
| ☒ | ☐ | Animals and other organisms |
| ☒ | ☐ | Human research participants |
| ☒ | ☐ | Clinical data |

## Methods

| n/a | Involved in the study | |
|---|---|---|
| ☒ | ☐ | ChIP-seq |
| ☒ | ☐ | Flow cytometry |
| ☒ | ☐ | MRI-based neuroimaging |

## Antibodies

| | |
|---|---|
| Antibodies used | Antibodies used:<br><br>Anti-HA (Thermo Fisher Scientific, Product #MA1-12429, Lot # MC1404501), anti-TAP (Invitrogen, Catalog # CAB1001, Lot # TA261224A); anti-myc (Sigma-Aldrich, Catalog # 9e10); IgG (Sigma-Aldrich, Catalog # I5006-100MG, several lot numbers used for the experiments); anti-Rlp24, anti-Nog2, Anti-Nsa2, and anti-Arx1 are gifts from Cosmin Saveanu and Micheline Fromont-Racine (Institut Pasteur, France); anti-Sda1 is a gift from Doug Kellogg (University of California Santa Cruz); anti-Erb1 is a gift from Jesús de la Cruz (Universidad de Sevilla, Seville, Spain); anti-Bud20 and anti-Nug1 are gifts from Vikram Panse (ETH Zürich, Germany); anti-Nog1 is a gift from Janine Maddock (University of Michigan); anti-rpL8 is a gift from Arlen Johnson (University of Texas at Austin), anti-rpL1 is a gift from Francois Lacroute (C.N.R.S. Gif-sur-Yvette, France); anti-rpL17 and anti-rpL26 are gifts from Sabine Rospert (Univeristy of Freiburg, Germany); ; anti-L25 is a gift from Katja Siegers (Technical University of Munich, Germany); Anti-Nop7, anti-rpL5 and anti-rpL11 were generated by the Woolford lab. |

Validation

Anti-HA antibody:

HA Tag (hemagglutinin tag) is an epitope tag that provides a method to localize gene products in a variety of cell types, study the topology of proteins and protein complexes, identify associated proteins, and characterize newly identified, low abundance or poorly immunogenic proteins when protein specific antibodies are not available. HA (hemagglutinin) is a surface glycoprotein derived from influenza hemagglutinin, and is commonly expressed as an epitope tag in conjunction with proteins to aid in identification and purification of the tagged protein for functional analysis. HA tag antibodies can be used to facilitate protein detection or co-immunopreciptation of tagged proteins.

https://www.thermofisher.com/order/genome-database/dataSheetPdf?producttype=antibody&productsubtype=antibody_primary&productId=MA1-12429&version=75

Anti-TAP antibody:

Epitope tags provide a method to localize gene products in a variety of cell types, study the topology of proteins and protein complexes, identify associated proteins, and characterize newly identified, low abundance or poorly immunogenic proteins when protein specific antibodies are not available. Tandem affinity purification (TAP) is an affinity purification method for isolation of TAP-tagged proteins together with associated proteins. The protocol involves the fusion of the "TAP tag" (typically a calmodulin binding peptide (CBP), a tobacco etch virus protease (TEV protease) cleavage site and Protein A) to the protein of interest. The TAP technique is useful in analyzing in vivo interactions.

https://www.thermofisher.com/order/genome-database/dataSheetPdf?producttype=antibody&productsubtype=antibody_primary&productId=CAB1001&version=75

Anti-myc antibody:

MYC (MYC proto-oncogene) is a transcriptional factor and oncoprotein. c-myc is a member of MYC gene family.[8] It is located on human chromosome 8q24. c-Myc gene codes for basic helix-loop-helix/leucine zipper (bHLH/LZ) transcription factor.

Monoclonal Anti-c-myc (mouse IgG1 isotype) is derived from the 9E10 hybridoma produced by the fusion of mouse myeloma cells and splenocytes from immunized BALB/c mice. It recognizes both N- terminal and C-terminal c-Myc tagged proteins. Monoclonal anti-c-Myc antibody produced in mouse has been used for Western blotting and immunohistochemistry.

https://www.sigmaaldrich.com/content/dam/sigma-aldrich/docs/Sigma/Datasheet/4/m5546dat.pdf

IgG antibody product information:

Purified rabbit IgG is isolated from pooled normal rabbit serum by fractionation and ion-exchange chromatography. Purified rabbit IgG may be used as a reference antigen, standard, blocking agent, or coating protein in a variety of immunoassays including ELISA, dot immunobinding, western immunoblotting, immunodiffusion, and immunoelectrophoresis. Other applications include starting materials for the preparation of immunogens and solid phase immunoadsorbents.

In this manuscript, IgG was used for affinity purifications of preribosomes. Since the TAP tag contains protein A, any IgG can be used to affinity purify or detect TAP-tagged proteins..

https://www.sigmaaldrich.com/content/dam/sigma-aldrich/docs/Sigma/Product_Information_Sheet/2/i5006pis.pdf

Anti-Rlp24 antibody:

Rabbit polyclonal Rlp24-specific antibody was generated in Micheline Fromont-Racine's lab (Institut Pasteur, France) by using recombinant glutathione S-transferase (GST)-Rlp24 purified from Escherichia coli.

Reference: http://doi.org/10.1128/MCB.23.13.4449-4460.2003

Anti-Nog2 antibody:

Rabbit polyclonal Nog2-specific antibody was generated in Micheline Fromont-Racine's lab (Institut Pasteur, France) by using recombinant glutathione S-transferase (GST)-Nog2 purified from Escherichia coli.

Reference: http://doi.org/10.1128/MCB.23.13.4449-4460.2003

Anti-Nsa2 antibody:

Rabbit polyclonal anti-Nsa2 antibody was produced by immunization of rabbits with recombinant GST-Nsa2 fusion protein produced in Escherichia coli BL21.

Reference: http://doi.org/10.1074/jbc.M602199200

Anti-Arx1 antibody:

Rabbit polyclonal anti-Arx1 antibody was produced by immunization of rabbits with specific immunogenic peptides and were affinity-purified (Covalab).

Reference: http://doi.org/10.1074/jbc.M602199200

Anti-Sda1 antibody:

Antibodies that recognize Sda1 were raised by immunizing rabbits with a COOH-terminal fragment of Sda1, purified from bacteria as a glutathione S-transferase (GST) fusion protein. The COOH-terminal fragment was amplified by PCR and cloned into the BamHI and EcoRI sites of pGEX-1 to create pZZ8, which expresses the COOHterminal fragment as a GST fusion. An identical fragment of Sda1 was cloned into the BamHI and EcoRI sites of pMAL-c2 to create pZZ6, which expresses the COOH-terminal fragment as an MBP fusion (New England Biolabs, Beverly, MA). Sda1 antibodies were affinity purified from serum by using the purifiedSda1-MBP fusion protein coupled to Affi-gel 10 (Bio-Rad Laboratories, Hercules, CA) as previously described (Kellogg and Alberts, 1992).

Reference: https://www.ncbi.nlm.nih.gov/pmc/articles/PMC30578/pdf/mk000201.pdf

Anti-Erb1 antibody:

Rabbit polyclonal anti-Erb1 antibody was raised by immunizing rabbits with purified Erb1 protien at the facilities of the Service for Animal Production (University of Seville, Spain).
Reference: https://www.ncbi.nlm.nih.gov/pmc/articles/PMC4678814/pdf/gkv1043.pdf

Anti-Bud20 antibody:
Recombinant anti-Bud20 was produced in BL21 E. coli strain by IPTG induction and affinity purified using Ni sepharose (GE Healthcare, Uppsala, Sweden).
Reference: https://www.ncbi.nlm.nih.gov/pmc/articles/PMC3542530/pdf/msb201263.pdf

Anti-Nug1 antibody:
Rabbit Polyclonal anti-Nug1 antibody was generated in Vikram G Panse's laboratory (Institute of Medical Microbiology, University of Zurich, Zurich, Switzerland).
Reference: file: https://doi.org/10.7554/eLife.52474

Anti-Nog1 antibody:
Antibodies were raised against HIS6Nog1 expressed from pJF14 in E. coli BL21 as previously described for anti-Mtg2 antibodies (Datta et al. 2005). The antibodies were affinity purified using HIS6Nog1 transferred to nitrocellulose, as previously described (Salamitou et al. 1994) before use.
Reference: https://doi.org/10.1007/s00438-007-0233-1

Anti-rpL8 antibody:
Rabbit polyclonal Rpl8p was raised and affinity purified against HIS6-tagged Rpl8p expressed in E. coli.
References:
https://doi.org/10.1038/sj.emboj.7600547
https://www.embopress.org/action/downloadSupplement?doi=10.1038%2Fsj.emboj.7600547&file=embj7600547-sup-0001.pdf

Anti-rpL1 antibody:
To generate anti-rpL1 antibody, SSM1a (rpL11) was overexpressed in E. coli by being cloned into the plasmid pUHE21-2. Only the truncated Ssm1p protein (amino acids 2 to 182) was expressed at a level sufficient to allow its gel purification. Gel slices that contained the Ssm1p polypeptide were used to immunize New Zealand rabbits. The specificity and titer of rabbit antisera were tested by Western blot (immunoblot) analysis with extracts prepared fromBMH71-18 transformants overexpressing the truncated or the complete Ssm1p protein as well as total yeast protein extracts (53) from the wild-type strain.
Reference: https://doi.org/10.1128/mcb.15.9.5071

Anti-rpL17 antibody:
Polyclonal antibody against the antigen rpL17A was raised in rabbits (EUROGENTEC, Bel S. A.).
Reference: https://www.jbc.org/content/282/11/7809.full.pdf

Anti-L26 antibody:
Rabbit polyclonal antibodies were raised against peptides or purified proteins in rabbit: aRpl35 (dilution 1:20,000), aRpl19 (dilution 1:1,000), and aRpl26 (dilution 1:4,000) from EUROGENTEC (Bel S.A.).
Reference: https://doi.org/10.1038/ncomms13563

Anti-rpL25 antibody:
This antibody was received from Katja Siegers, Technology Manager at Technical University of Munich.
Reference: https://www.embopress.org/doi/epdf/10.1038/sj.embor.7400551 (Note: This manuscript has since been retracted. However, that is where we got the antibody from, and that is the only refeence about rpL25 antibody).

Anti-Nop7 antibody:
Rabbit polyclonal antibody against Nop7 was generated using a short peptide sequence GIKYSETSEADKDVNKSK, corresponding to amino acids 522 to 539 of Nop7, which was synthesized by Alpha Diagnostic International Inc. This peptide sequence was selected based on its hydrophilicity, antigenicity, and accessibility, as well as the sequence being highly specific to Nop7. The peptide was linked to KL11 carrier protein and was injected into rabbits by Alpha Diagnostic International Inc. Bleeds (5 mL) from the rabbits were collected every 8-11 weeks. This antibody was generated in the Woolford laboratory by a former graduate student, Piyanun Harnpicharnchai, and was published in her thesis: "Dynamics of Ribosome Assembly in Saccharomyces cerevisiae" (2004).
The reference below shows western blots using this antibody.
Reference: https://doi.org/10.1016/S1097-2765(01)00344-6

Anti-rpL5 antibody (Note: rpL1 is an old name for rpL5). :
Rabbit polyclonal antibody against rpL5 was generated by immunizing the rabbit with yeast 5S-rpL5 complex at the Berkeley Antibody Company (Richmond, CA). This was done by a former graduate student in the Woolford lab, Mohanish Deshmukh, and was published in his thesis: "A study of the assembly and interactions of yeast ribosomal protein L1 and 5S RNA" (1994).
The reference below shows western blots using this antibody.
Reference: http://genesdev.cshlp.org/content/21/20/2580

Anti-rpL11 antibody (Note: rpL16 is an old name for rpL11):

Construction of a trpE-RPL16 Gene Fusion and Isolation of Antisera against Ribosomal Protein L16: A 1.0 fragment extending from the fourth nucleotide of the RPLIGA coding region to 475 nucleotides 3' of the TAA stop codon was constructed as described by Tsay et al. (1988). This fragment was cloned into the BamHl (Spindler et al., 1984) to create pATH1-21 containing an inframe fusion between the E. coli trpE gene and the s. cerevisiae RPLl6A gene. E. coli RRI transformed with this plasmid produced a trpE-L16 fusion protein of the expected size, 57 kDa, after depletion of tryptophan and induction by P-indole-acrylic acid. The fusion protein was purified from the insoluble fraction of extracts prepared from the transformants by preparative SDS-polyacrylamide gel electrophoresis, electroelution, and dialysis into 0.02% SDS 0.01 M NH,HCO, (Spindler et al., 1984).

Rabbits were immunized by subcutaneous injection of 200 pg of purified trpE-L16 protein in 0.75 ml H,O emulsified with an equal volume of complete Freund's adjuvant. A total of 150 pg of immunogen in incomplete Freund's adjuvant was used for eight subsequent intramuscular injections. High titer antisera, assayed by Western immunoblotanalysis, were obtained subsequent to the third boost, and were stored at -20 "C.

Reference: https://www.jbc.org/content/269/10/7579.long
