# Supplementary material for: Coupling of 5S RNP rotation with maturation of functional centers during large ribosomal subunit assembly
Source: Nat Commun. 2020 Jul 27;11:3751. doi: 10.1038/s41467-020-17534-5 (PMC7385084; doi:10.1038/s41467-020-17534-5)
Supplement: Supplementary file 2 — Peer Review File [file 41467_2020_17534_MOESM2_ESM.pdf]

Reviewers' comments:

Reviewer #1 (Remarks to the Author):

This is an interesting study employing a combination of modern approaches to study the function of the C-terminal extension of ribosome assembly factor Rpf2. The authors provide convincing evidence that the Rpf2 CTD is required for correct folding of rRNA helices 68-70, allowing for efficient recruitment of Sda1 and consequent 5S RNP rotation. This is a high-quality study deserving publication in Nature Communications. There are however some aspects that should be improved, in particular:

- The northern blot analyses in this paper are not very informative. Only two precursors, 27S and 7S pre-rRNA are analyzed, and sometimes even the loading control is missing (Supplementary Figure 9). Moreover, a direct comparison between depletion of Rpf2 in the rpf2-delta-255-344 strain compared to the total depletion of Rpf2 would be interesting. The detection of more precursors would be helpful for the interpretation of the actual defect.

- The authors claim that they tested for stability of the truncated Rpf2 protein indirectly by testing the levels of Rrs1, rpL5 and rpL11, which are known to be dependent on stable Rpf2. It is good that they can show that these proteins are unaffected, but it would be nice to also have Rpf2 in the analysis. The authors argue that there is no antibody available and C-terminal tagging causes growth defects. But why don't they use an N-terminal tag, as they did for the variant under the control of the Gal-Promoter? This should be easy for the strain in which the truncated variant is provided from a plasmid. To be able to distinguish between the protein expressed from the Gal-Promoter and from the pRS315 plasmid, they could use some epitope tag other than HA, like Flag or Myc. If there is a reason why this is not possible, I would suggest to at least re-write the text a bit and start out with saying something like "We tested whether the truncated Rpf2 complex affects the stability of Rrs1, Rpl5 or Rpl11." As a final conclusion of the paragraph, they could then argue that considering that these proteins are mutually interdependent for their stability, it is likely that also Rpf2 is stable (rather than starting already out with Rpf2 stability).

Minor points
- The authors refer on multiple occassions to Nog2-TAP particle states 1-3 that they introduced in a previous paper but also to pre-60S particle states described by the Beckmann and Klinge groups. The many different states discussed in the paper are sometimes confusing. Supplementary Figure 1b is helpful for looking up the discussed states, but for example Nog2-TAP particle states 1-3 (which are most important for this paper) are not clearly labeled here (there are two Nog2 particles indicated but without naming the state).
To improve the clarity, the authors should include all discussed states in Supplementary Figure 1B (and mention all names for particles which have been described by more than one group). For example, the text mentions that Arx1/Nog2 State 1/and State F particles are similar – then the Arx1-particles in the scheme should be labeled Arx1/Nog2 State 1/and State F.

- The resolution of the images is bad and sometimes the relevant features in the structures are difficult to discern and should be better labeled; an example is Figure 4; only the missing densities are labeled, and only in State 3. It would be useful to have also labels for the existing densities. Moreover, the missing densities of H68-70 should also be labeled in Figure 4b (not only in 4c). Otherwise readers might confuse the missing density of H68-70 in 4b (left) with the 5S RNA, which is also in red an labeled in Figure 4a.
I also wonder why the 5S RNP part is not colored in Figure 4b. In Figure 4a (left), representing the "perfect fit", the colors are visible, so shouldn't they also be visible in the states in 4b where the 5S RNP is in the same position?

- The picture in Figure 8a, indicating conformational changes of H68-71 is in principal nice. However,

only structures from previous studies are shown. It would be much more interesting to the reader, if these were compared with the conformations found in the current study.

- From the point in the paper where the missing densities of H68-70 are mentioned, the reader wonders what is already known about maturation of these helices and if there are known structures in which these helices are not yet in their mature conformation. This is nicely summarized in the discussion section, but that is too late in my opinion. Also the suggested comparison of the H68-71 conformations (Figure 8a, see above) should be shown earlier in the manuscript.

- Figure 3C is a bit difficult to read – it would be helpful to have a color legend directly in the Figure so that one does not need to look up which color is what in the Figure legend.

- Page 12, line 246: Levels of Nop53 were also decreased….indicating that the ITS2…processing may be affected.
The basis for this interpretation should be briefly explained in the text (e.g. reduced exosome recruitment…).

- There are some small spelling mistakes: page 6, line 126: rearagnements; page 23, line 435: though (through?); page 26, line 493: Rfp2 instead of Rpf2

- Methods section: this section is not very detailed. It would be extremely useful to have a list of strains and plasmids in the paper, and not just upon request as stated. Also, the methods descriptions could be more detailed. Large parts of the methods only refer to previous studies.

Reviewer #2 (Remarks to the Author):

In the manuscript "Coupling of 5S RNP rotation with maturation of functional centers during middle stages of large ribosomal subunit assembly", Woolford and coworkers performed a combined genetic and structural analysis in the yeast Saccharomyces cerevisiae to investigate how the 5S RNP and nearby regions on the pre-60S particle mature depending on the C-terminal tail of the Rpf2 assembly factor.

While this study contributes to a better understanding of ribosome biogenesis by revealing some specific aspects regarding the role of the C-terminal extension of Rfp2 for 5S RNP rotation within the pre-60S subunit, it remains a bit short on what this really means in terms of the exact role of the C-terminus of Rpf2 for these middle stage 60S assembly steps. In particular, 'the checkpoint theory in subunit maturation' as written in the abstract appears to be very overstated.

The main focus was a structural comparison between wild-type Nog2 particles and particles isolated from the rpf2 mutant lacking the C-extension under growth-retarded conditions. It was observed that some distinct 25S rRNA helices (H68-70) and a few assembly factors were affected in this mutant, which prompted the authors to speculate on the mechanistic implications.
It was surprising that the deletion of the Rpf2 C-terminus did not affect the overall association of Rpf2 and the 5S RNP with the pre-60S particle. However, the way how this was analysed cannot fully distinguish between different scenarios. Therefore, it is important to rigorously show that the deletion of the Rpf2 C-terminus only affects the middle stage biogenesis steps (which is a major conclusion of this study), but not the first steps of 5S RNP assembly into the early pre-60S particles.

Specific points
It is crucial to analyze the middle stage pre-60S particle, e.g. purified by Nop7, suggested to contain Rpf2∆C, Rrs1, Rpl5, Rpl11 and the other biogenesis factors in stoichiometric amounts, by SDS-PAGE and Coomassie staining. Rpf2∆C could be easily HA-tagged like it was done for wild-type Rpf2 and

hence should be clearly visible later on gels.

Supplementary Figure 4a and 4c: These experiments lack both an important control to demonstrate that depletion of Rpf2-HA per se has no effect on the composition of Nop7-TAP and Nog2-TAP particles.

Supplementary Figure 4a: the authors refer to a control already published in 2007 ("Assembly factors Rpf2 and Rrs1 recruit 5S rRNA and ribosomal proteins rpL5 and rpL11 into nascent ribosomes"), but re-depicted in Figure 3. Comparing this new Nop7-TAP purification under GAL condition with the old but same purification, there is a significant difference in the pattern of co-enriched bands. It would be important to perform side-by-side a new control under the same conditions, which only would allow to directly compare these results.
Supplementary Figure 4c: please include a SDS-PAGE reference for the western from the Nog2-TAP purification, similar to Figure 4a.

Page 6, lanes 135-137: Include references

Page 7, lane 156: Properly "folded" seems not the best term for an unstructured, flexible protein extension.

Page 9, lane 189-196: Despite it was previously shown that protein levels of the analysed proteins are interdependent, it would be necessary to show the stability of the Rpf2 truncated protein by a western blot analysis of cell lysates (apparently Supplementary Fig. 4a does not include whole cell lysates but only eluates). Moreover, L5 and L11 westerns (Supplementary Fig. 4a) should be improved.

Page 12, lanes 253-255: It is mentioned that classes C2 and C3 were combined since the difference was "only" in presence/absence of a few assembly factors. Still, it would be important to mention, which assembly factors in fact were present/absent, compared to wild-type particles.

Page 16, lanes 321-322: It is not evident that the Rpf2 CTD is directly involved in H68-70 maturation as suggested in the text. It could be also indirect via affecting the positioning of other factors necessary for this maturation (e.g. the first two alpha-helices of Nog2 that interact with this rRNA region).

Supplementary Fig. 2a: It is rather surprising that Rpf2 co-purifies the 90S factor Mpp10. This remains to be further addressed in this study. Therefore, the authors should not only analyse Tsr1-TAP and late cytoplasmic Lsg1-TAP, but also perform affinity-purification of "middle stage" (e.g. Nog2, Nsa2) and nucleolar factors (e.g. Nsa1) that are not present on 90S particles. Also loading controls (bait protein and r-protein westerns) and/or Coomassie staining would be important to validate the results.

The title of Supplementary Fig. 2 should be revised, as this figure does not give information at which stage pre-60S maturation is affected ("yet truncation of its CTD affects subunit assembly at later steps"). Since there is also a nucleolar L25-GFP signal (which normally is not the case in mutants with a later nucleoplasmic defect), it is not obvious (at least from this figure) whether there is also an early pre-60S biogenesis defect.

Supplementary Fig. 2c: it would be necessary to show the polysome profiles with (N-terminally) epitope tagged Rpf2 and Rpf2Δ255-344 and assess them by western blotting, in order to reveal how well the mutant is binding to the particles and to compare the free/pre-60S-bound levels of wild-type vs mutant protein. In addition, a western blot against Nog2 could clarify whether Nog2 pre-60S association in the rpf2 mutant is indeed completely unaffected as mentioned in the manuscript (e.g. on page 12, lanes 238-240).

Figure 3: On the silver-stained gel only the bands corresponding to Nog2-TAP, Arx1, Sda1, Rea1 are

indicated. Can also other major bands be indicated for a better understanding on the protein pattern? On page 12, lanes 246-247, it is mentioned that Nop53 is decreased and ITS2 processing is therefore affected. This is not apparent from the stained gel, where Nop53 does not significantly change, in contrast to the iTRAQ data suggested. What is the explanation for this discrepancy?

Reviewer #3 (Remarks to the Author):

This manuscript explores the effect of removing the C-terminal end of assembly factor Rpf2 in the maturation of the functional centers of assembling 60S subunits in yeast. To this end, the authors follow mainly a cryo-EM structural approach that leads to the obtention of a structure of an immature 60S subunit assembled in a strain expressing a variant of the assembly factor Rpf2. A comparison of the obtained structure with previously reported structures of other assembly intermediates allows the authors to infer on the roles of the C-terminal end of Rpf2 in ribosomal assembly.

The quality of the structural work is excellent and certainly at the level expected from readers in Nature Communications. The text is well written, and descriptions of the structures are clear. However, the main issue I see in the present form of the manuscript is that the design of the figures makes very difficult to visualize the highlighted structural differences and convey a clear message. It will be important that the design of the structural figures is improved to make a convincing case and solidify the conclusions derived from the structures. Without access to the cryo-EM maps and molecular models derived from these maps is not possible to validate some of the conclusions and statements made by the authors only based on the provided figures.

I will provide now some specific feedback in the figures to illustrate this point and hopefully help the authors in creating a more efficient set of figures:

1. I think it is a great idea to add a supplementary figure to assist the comprehension of the process in the introduction describing the assembly of the 5S RNP and stable association with the ribosomal particle. However, I found very difficult to correlate the text on page 4, which refers to Suppl Fig. 1b to what is shown in this panel. Unless the reader is familiar with the maturation process of the 60S subunit, this figure is not very helpful.

2. Figure 4 illustrates the docking of the molecular model of State 1 Nog2 particle into the cryo-EM map obtained for the pre-ribosomal 60S particle from the C-terminal deletion mutant of rpf2 and on maps for State 1 and 3 of Nog2 particles. The difference between the 5S RNP not rotated in Stage 1 and rotated in stage 3 is not visible in the provided views in panel A. Panel C, left is also confusing. I can see there is no density representing the C-terminal end of Rpf2, but the image is very crowded. Overall this figure would benefit and become clearer if the authors were to make use of a higher level of transparencies for the cryo-EM map and also an efficient use of segmentation tools for example in the Chimera program. This would contribute to declutter the images and allow to easily see what the text is describing.

3. Similarly, I don't find Fig 5A particularly effective in showing the altered configuration of H75 in the three different structures. Panel C could also be improved by zooming into the relevant regions showing differences (GTPase domain and the C-terminal domain).

4. Fig 7 is quite clear. However, I think using a light grey colour for the non-coloured regions of the maps will make a clearer figure. A good way to also show the differences between Class c2 and C3 would have been difference map analysis. The authors should consider this approach to highlight the differences between both maps.
5. Suppl. Fig 8 would also benefit from using a light grey colour for the map and density segmentation

tools could be used to easily visualize the different positions adopted by L1.

A few additional comments non-related to the figure design follows:

1. Supplementary figures should include the FSC plots and local resolution analysis of the presented structures.

2. It is not mentioned anywhere in the manuscript that the obtained cryo-EM maps and derived molecular models have been properly deposited in the EMDB and PDB. EMDB and PDB IDs of these depositions are not provided. This is a requirement for publication in this and any other reputable journal.

3. In the 3D classification of the cryo-EM maps shown in Supple Fig 5b, the authors do not comment in what could be the origin of the 'bad particles'. What makes them bad and what do they represent? They represent about 31-32% of the data. The data set classified in Suppl. Fig 7b only shows a ~2% of bad particles. Could this difference be explained somehow? If these are particles damaged by the water-air interface, why the percentage of damaged particles is much smaller in the dataset in Suppl. Fig. 7.

In response to reviewer #1 (reviewer's comments are in *italics*):

1. The northern blot analyses in this paper are not very informative. Only two precursors, 27S and 7S pre-rRNA are analyzed, and sometimes even the loading control is missing (Supplementary Figure 9). Moreover, a direct comparison between depletion of Rpf2 in the rpf2-delta-255-344 strain compared to the total depletion of Rpf2 would be interesting. The detection of more precursors would be helpful for the interpretation of the actual defect.

We have repeated the analysis of pre-rRNA intermediates for most of the relevant strains, using northern hybridization and primer extension assays. New data include a loading control for northern hybridizations (Supplementary Figure 4e). These results were used to adjust for loading prior to performing primer extension assays (there is no appropriate loading control for that assay). Direct comparison between the total depletion of Rpf2 (labeled *GAL-HA-RPF2 + empty vector*) and depletion of Rpf2 in the *rpf2Δ255-344* strain (labeled *GAL-HA-RPF2 + rpf2Δ255-344*) is shown in new Supplementary Figures 4e, f, and g. We added data for levels of mature 25S, 18S, 5.8S and 5S rRNAs, as well as data for the earlier intermediates, namely $27SA_2$, $27SA_3$, and $27SB_S$ and B_L pre-rRNAs (analyzed by primer extension). Together, these data confirm that the block in pre-rRNA processing upon either depleting Rpf2, or truncating the C-terminus of Rpf2 is at the level of 27SB and 7S pre-rRNA precursors, corresponding to middle steps of 60S ribosomal subunit assembly. However, the truncated Rpf2 protein is present in pre-ribosomes (see below), and there are important differences in effects on pre-ribosomes between depletion of Rpf2 and truncation of the C-terminal domain of Rpf2.

We also added pre-rRNA processing data for depletion of Sda1, not originally in the manuscript (Supplementary Figures 7b,c).

For the *rpf2-2* mutant, we added data for changes in levels of mature 25S and 18s rRNAs (Supplementary Figure 7f). We were unable to obtain a loading control for northern hybridization due to technical difficulties. Instead, we have added primer extension data for this mutant (Supplementary Figure 7g). Since the same loading was used to perform experiments depicted in Supplementary Figures 7f and 7g, and the only significant change caused by expression of the *rpf2-2* mutant protein is 7S pre-rRNA accumulation, we believe that adding primer extension data is sufficient to serve as "loading control" in this case. Alternatively, since these data are not crucial for understanding of the defect presented in the manuscript, RNA data for the *rpf2-2* mutant may be removed if needed.

2. *The authors claim that they tested for stability of the truncated Rpf2 protein indirectly by testing the levels of Rrs1, rpL5 and rpL11, which are known to be dependent on stable Rpf2. It is good that they can show that these proteins are unaffected, but it would be nice to also have Rpf2 in the analysis. The authors argue that there is no antibody available and C-terminal tagging causes growth defects. But why don't they use an N-terminal tag, as they did for the variant under the control of the Gal-Promoter? This should be easy for the strain in which the truncated variant is provided from a plasmid. To be able to distinguish between the protein expressed from the Gal-Promoter and from the pRS315 plasmid, they could use some epitope tag other than HA, like Flag or Myc. If there is a reason why this is not possible, I would suggest to at least re-write the text a bit and start out with saying something like "We tested whether the truncated Rpf2 complex affects the stability of Rrs1, Rpl5 or Rpl11." As a final conclusion of the paragraph,*

they could then argue that considering that these proteins are mutually interdependent for their stability, it is likely that also Rpf2 is stable (rather than starting already out with Rpf2 stability).

We thank the reviewer for the idea to epitope-tag our mutant protein at the N-terminus, expressed from a plasmid. We have worried that the lack of western blotting for the truncated Rpf2 is a weak point of the manuscript, but did not want to go into an elaborate N-terminal tagging in the genome, or express the truncation from the *GAL-HA* promoter. The idea to tag it in the plasmid (considering that there is no difference in phenotypes regardless of whether *rpf2Δ255-344* is expressed from a plasmid or the genome) was very helpful. Plasmids expressing N-terminally Myc-tagged wild-type and truncated *RPF2* were transformed into the appropriate yeast strains (*GAL-HA-RPF2 RRS1-HA NOP7-TAP and GAL-HA-RPF2 NOG2-TAP*).

First, we tested for complementation of the effect of Rpf2 depletion in glucose medium. The plasmid expressing wild-type 2Myc-Rpf2 was able to rescue the lethal phenotype caused by depletion at all temperatures, while the plasmid expressing 2Myc-rpf2Δ255-344 was only able to complement growth at 30°C, but not at 16°C, the same as the untagged rpf2Δ255-344 mutant protein.

Second, we confirmed that both wild-type and mutant Rpf2 proteins were epitope-tagged, and that both proteins as well other protein components of the 5S RNP complex were stably expressed, by western blotting using whole-cell extracts, as well as western blotting of fractions from sucrose gradient centrifugation (Supplementary Figures 4b,c,d, also see our response to comment #13 for reviewer #2).

Third, pre-60S assembly intermediates were isolated from strains expressing Myc-tagged truncated rpf2 protein using Nop7-Tap and Nog2-Tap, respectively (Supplementary Figure 5b,c). We were able to confirm that truncated rpf2Δ255-344 protein (and other protein constituents of the Rpf2 subcomplex) were stably expressed and able to enter maturing 60S subunits. The truncated rpf2 protein could join assembling subunits even in galactose, in the presence of wild type Rpf2. More of it enters pre-ribosomal subunits in glucose, when there is no competition between wild-type and mutant protein for entry into the particles. Although the western blot signal for Rrs1-HA was quite weak, and the signal for rpL11 was a little "smudged", considering our previous data, we are confident that all proteins are able to stably enter pre-ribosomal particles.

Our new data showed that Nog2 actually accumulates in Nop7-Tap purifications from the *rpf2Δ255-344* mutant strain, which makes even more sense, since truncation of the CTD of Rpf2 causes a block during the lifetime of Nog2, and, thus causes accumulation of Nog2-containing particles. However, regardless of whether there is more Nog2, or the same amount of Nog2 as in a control strain, for the purpose of this manuscript it is only important that levels of Nog2 did *not decrease* upon truncation of the CTD of Rpf2.

3. The authors refer on multiple occassions to Nog2-TAP particle states 1-3 that they introduced in a previous paper but also to pre-60S particle states described by the Beckmann and Klinge groups. The many different states discussed in the paper are sometimes confusing. Supplementary Figure 1b is helpful for looking up the discussed states, but for example Nog2-TAP particle states 1-3 (which are most important for this paper) are not clearly labeled here (there are two Nog2 particles indicated but without naming the state).
To improve the clarity, the authors should include all discussed states in Supplementary Figure 1B (and mention all names for particles which have been described by more than one group). For

example, the text mentions that Arx1/Nog2 State 1/and State F particles are similar – then the Arx1-particles in the scheme should be labeled Arx1/Nog2 State 1/and State F.

We have now significantly altered many of our figures, including Supplementary Figure 1b, to make them more understandable for all readers. We realized that Supplementary Figure 1b was too complicated and detailed for readers who are not familiar with assembly of 60S ribosomal subunits in yeast (also see comment #1 from reviewer #3 and our response). Thus, our revised figure now depicts only assembly intermediates from middle stages of assembly, in order to guide readers through the introductory part of the manuscript, which is written to help readers understand the part of the 60S assembly pathway investigated in this manuscript. We believe that this figure is now much more informative.

4. The resolution of the images is bad and sometimes the relevant features in the structures are difficult to discern and should be better labeled; an example is Figure 4; only the missing densities are labeled, and only in State 3. It would be useful to have also labels for the existing densities. Moreover, the missing densities of H68-70 should also be labeled in Figure 4b (not only in 4c). Otherwise readers might confuse the missing density of H68-70 in 4b (left) with the 5S RNA, which is also in red an labeled in Figure 4a.
I also wonder why the 5S RNP part is not colored in Figure 4b. In Figure 4a (left), representing the "perfect fit", the colors are visible, so shouldn't they also be visible in the states in 4b where the 5S RNP is in the same position?

We apologize to the reviewer that the images were not high enough resolution or clear enough. All of our original images were saved in resolution of 300 dpi, but during conversion to PDF files for submission, the resolution was decreased automatically.

To address comments from reviewers #1 and #3 about figures depicting our structural data, we have now completely changed the way that we present all of these data. We originally used the method of coloring relevant parts of the wild type Nog2 atomic model (i.e., parts affected in the *rpf2Δ255-344* mutant), and then fitting this colored atomic model into density maps of both wild type and mutant particles. Thus, proteins and rRNA helices that are absent or flexible in mutant particles (and consequently invisible by cryo-EM), were shown (since densities are missing and do not cover colored atomic models), and the ones that are present were not shown (densities are not missing). Even though this is a proper way to present structural data, our concern was that this way of displaying them is counterintuitive and may confuse a broad readership. Thus, in the revised version of the manuscript, we now color proteins and rRNAs that are present, and if they are missing or flexible, they are not colored. To do that, we used the following strategies:

To show defects in the C4 class of *rpf2Δ255-344* mutant particles:

We (1) used the Depiction Tool Color Zone in Chimera, (2) we compared wild-type and mutant atomic models directly, and (3) we fitted the wild-type atomic model to the wild-type density map, and the mutant atomic model to the mutant density map. Combining these three approaches allowed us to show our data so that what is not shown on structures is invisible in the mutant density maps.

To show defects in particles depleted of Sda1:

Since we do not have an atomic model for either class of *GAL-SDA1* particles due to the medium resolution of the map, we fitted the atomic model of wild-type particles into density maps

of mutant particles, and subsequently used Depiction Tool Color Zone to color densities of mutant particles. Alternatively, the transparency of the mutant maps was used to show whether proteins or rRNA helices of interest are affected upon depletion of Sda1 (Figure 4c only). If applicable (in cases where densities for proteins or rRNA helices were missing), parts of the wild type atomic model corresponding to missing densities were deleted, and the Color zone Tool was then used.

To show defects in the C1, C2, and C3 classes of *rpf2Δ255-344* particles:

We fitted the atomic model of the C4 class into density maps of the C1, C2 and C3 particles, and subsequently used Depiction Tool Color Zone to color densities of relevant components of mutant maps. If applicable (in cases where densities for proteins or rRNA helices were missing), parts of the C4 class atomic model corresponding to missing densities were deleted, and the Color zone Tool was then used.

We also used a density segmentation tool to present differences in L1 stalk orientations between wild-type and mutant density maps (Figure 9c).

In addition, to prevent overcrowding of images, we have changed the transparency, depth of field, silhouettes, and other depiction tools. We have also limited the number of proteins and rRNA helices we show per figure. For example, in Figure 4c (new Figure 3b), instead of showing changes in H68-70, the CTD of Rpf2, Rsa4 and Nog2, we only show H68-70 and the CTD of Rpf2. Then, we show changes in Rsa4 and Nog2 in subsequent figures. That way, no information is lost, and a figure for each molecule corresponds to the timeline when they are being mentioned in the Results section. We believe that this change makes the flow of the Results section much better.

We have also changed the color of 5S rRNA to pink, so that it does not get confused with H68-71.

We ended up using the original method of presenting differences in density maps between wild-type and mutant strains in one figure (previous Supplementary Fig. 8, new Figure 9a,b). Please see our response to comment #5 of reviewer #3 for the reason behind this decision.

We believe that the above changes significantly improved representation of the defects caused by truncation of the CTD of Rpf2 protein and depletion of Sda1.

5. The picture in Figure 8a, indicating conformational changes of H68-71 is in principal nice. However, only structures from previous studies are shown. It would be much more interesting to the reader, if these were compared with the conformations found in the current study.

In work presented in this manuscript, we were not able to visualize H68-71, since they are flexible and thus invisible by cryo-EM. Therefore, we have no additional conformations to add to Figure 8a (new Figure 4a). This figure was meant to be used as a way to discuss the importance of these helices.

6. From the point in the paper where the missing densities of H68-70 are mentioned, the reader wonders what is already known about maturation of these helices and if there are known structures in which these helices are not yet in their mature conformation. This is nicely summarized in the discussion section, but that is too late in my opinion. Also the suggested comparison of the H68-71 conformations (Figure 8a, see above) should be shown earlier in the manuscript.

We have now moved Figure 8a earlier in the manuscript, right after we first mention that those helices are flexible and thus “invisible” by cryo-EM, and before we talk about the fact that Sda1 binds to them (new Figure 4a). We believe that this change improves the flow of the Results section, and we thank the reviewer for this suggestion.

7. Figure 3C is a bit difficult to read – it would be helpful to have a color legend directly in the Figure so that one does not need to look up which color is what in the Figure legend.

Thank you. We have now added a color legend to the bottom of Figure 3c (new Figure 2c).

8. Page 12, line 246: Levels of Nop53 were also decreased….indicating that the ITS2…processing may be affected. The basis for this interpretation should be briefly explained in the text (e.g. reduced exosome recruitment…).

We have now added a sentence elaborating on the meaning of the decrease in levels of Nop53, stating “Since Nop53 recruits the exosome machinery to ITS2, processing of this spacer RNA may be affected in some assembling 60S particles lacking Nop53”. We have also added a new, recently published reference (ref. 38, Cepeda et al, “The ribosome assembly factor Nop53 controls association of the RNA exosome with pre-60S particles in yeast”, 2019).

9. There are some small spelling mistakes: page 6, line 126: rearagnements; page 23, line 435: though (through?); page 26, line 493: Rfp2 instead of Rpf2

Thank you. We have now corrected all of the spelling mistakes.

10. Methods section: this section is not very detailed. It would be extremely useful to have a list of strains and plasmids in the paper, and not just upon request as stated. Also, the methods descriptions could be more detailed. Large parts of the methods only refer to previous studies.

We have now significantly improved the Methods section, by adding more detailed descriptions of our methods (buffers used, incubation times and similar details). The only instances where we did not do that was in sections where we refer to published Methods papers (ref. 51, 52), since they contain detailed information about how to perform gene tagging and other genetic manipulations. Also, we have now added Supplementary Tables 1 and 2, with our list of strains and plasmids, respectively.

In response to reviewer #2 (reviewer’s comments are in *italics*):

1.While this study contributes to a better understanding of ribosome biogenesis by revealing some specific aspects regarding the role of the C-terminal extension of Rfp2 for 5S RNP rotation within the pre-60S subunit, it remains a bit short on what this really means in terms of the exact role of the C-terminus of Rpf2 for these middle stage 60S assembly steps. In particular, ‘the checkpoint theory in subunit maturation’ as written in the abstract appears to be very overstated.

Thank you for your concern that our explanation of the role of the CTD of Rpf2 in 5S RNP rotation is not as clear is it should be. To address this comment, we have made multiple changes to more clearly explain the *rpf2Δ255-344* phenotype. We have now re-written parts of the Abstract,

Results and Discussion. In particular, we have now emphasized the potential importance of the GTPase Nog2 in transmitting a signal from the CTD of Rpf2 to helices H68-70. Thus, the absence of the extension of Rpf2 may affect H68-70 directly, or it may affect it by affecting Nog2, which in turn affects the conformations of H68-70. At this time, we are unable to perform any other experiments that would help distinguish between these two models.

We have also better explained our "checkpoint" theory by emphasizing the importance of the proper conformation of H68-70, which may be a signal for correctly undergoing middle steps of assembly. Thus, failure to properly mature these helices may be a signal to turn over misassembled particles. Both truncation of Rpf2 and depletion of Sda1 cause a block in 5S RNP rotation. However, the *important and major* difference between these two mutant strains is that in the absence of the CTD of Rpf2, helices H68-70 are misfolded, which is *not the case* upon depletion of Sda1. Thus, the improper conformation of these helices in mutant particles delays further assembly events. In other words, conformational maturation of H68-70 (as seen in the Nog2 State 1) is a prerequisite for entering the next major stages of 5S RNP rotation and nuclear export. Therefore, in this context we speculate that the conformation maturation of H69-70 is a checkpoint in the middle steps of assembly. We have now improved this statement in the Discussion part of the manuscript (page 11, lanes 441-442, and 444-446).

2. It was surprising that the deletion of the Rpf2 C-terminus did not affect the overall association of Rpf2 and the 5S RNP with the pre-60S particle. However, the way how this was analysed cannot fully distinguish between different scenarios. Therefore, it is important to rigorously show that the deletion of the Rpf2 C-terminus only affects the middle stage biogenesis steps (which is a major conclusion of this study), but not the first steps of 5S RNP assembly into the early pre-60S particles.

The main critique described by the reviewer here is that we needed to rigorously show that the deletion of the Rpf2 C-terminus only affects the middle stages in 60S ribosomal subunit biogenesis, and not the entry of the Rpf2 subcomplex into pre-ribosomes during early stages of subunit assembly. As explained in the text (and in our previous publication Zhang et al, 2007), the constituents of the Rpf2 subcomplex are interdependent both for their entry into pre-ribosomes and for their stability. Our previously published RNA data (Zhang et al, 2007), and the experiments now added to this manuscript, show that depletion of the Rpf2 protein does not affect pre-rRNA processing until the middle stages of subunit assembly, processing of 27S and 7S pre-rRNAs. Thus, it is not surprising that the C-terminal truncation of Rpf2 would have the same defect in maturation of pre-ribosomal particles as depletion of the entire protein. It would be rather surprising to see that the truncation of the CTD has an *earlier* defect than the depletion of the whole protein. We had shown that in the absence of the CTD of Rpf2, other members of the 5S RNP can enter pre-ribosomes. That is why in the Introduction we specifically point out that "Depletion of each of the proteins in the Rpf2-subcomplex does not affect biogenesis of the 60S subunit until middle stages of assembly, after the 5S RNP/Rpf2/Rrs1 bound to rRNA domain V anchors to the assembling particles (refs. 20, 28). Taken together, these observations suggest that the Rpf2-subcomplex, and rRNA domain V (helices H80, H82-88), with which it first associates, are initially docked in a flexible state and do not influence assembly of the remaining portions of nascent subunits *until they become stably anchored within Nog2 particles*".

However, we understand the importance of this critique, particularly because we did not show whether our truncated protein was stable or not, but indirectly deduced that it was stable based on stability of other members of the Rpf2 subcomplex. To resolve this issue, we have now

Myc-tagged both the wild type and mutant Rpf2 proteins, and performed a series of experiments to address all of the reviewer's comments (see below).

3. It is crucial to analyze the middle stage pre-60S particle, e.g. purified by Nop7, suggested to contain Rpf2ΔC, Rrs1, Rpl5, Rpl11 and the other biogenesis factors in stoichiometric amounts, by SDS-PAGE and Coomassie staining. Rpf2ΔC could be easily HA-tagged like it was done for wild-type Rpf2 and hence should be clearly visible later on gels.

As stated above in response to comment #2 of Reviewer#1, we have now tagged both wild-type and truncated Rpf2 proteins with a double Myc tag at their N-termini and confirmed that they are expressed and stable. We repeated affinity-purifications using TAP-tagged Nop7 as a bait and were able to confirm that Myc-tagged truncated Rpf2 protein, as well as the other protein constituents of the Rpf2 subcomplex, are entering assembling 60S subunits (Supplementary Figure 5b). Truncated rpf2 protein can join assembling subunits even in the presence of wild-type Rpf2 (galactose media), but more of it enters pre-ribosomal subunits in glucose, when there is no competition between wild type and mutant protein for entry into pre-ribosomes. Levels of Nog2 in pre-ribosomal subunits increase in the presence of the truncated version of the Rpf2 protein. More Nog2-containing particles are accumulating due to a block in assembly and are being co-purified by Nop7 bait (please also see our detailed response to Comment #2 for reviewer #1). We also added another independent loading control, rpL25.

4. Supplementary Figure 4a and 4c: These experiments lack both an important control to demonstrate that depletion of Rpf2-HA per se has no effect on the composition of Nop7-TAP and Nog2-TAP particles.

The control experiment that the reviewer is asking for Supplementary Figure 4a (new Supplementary Figure 5b) was previously published (Zhang et al, 2007), and shows that depletion of Rpf2 has an effect on the composition of Nop7-Tap particles. Specifically, in the absence of Rpf2, other protein constituents, as well as 5S rRNA and Nog2, cannot enter assembling 60S subunits and are unstable. In contrast, as shown in this manuscript, when the truncated rpf2 protein is present, those molecules can enter pre-60S subunits.

The same control experiment cannot be performed for Supplementary Figure 4c (new Supplementary Figure 5c) with Nog2-Tap, since, as mentioned above, in the absence of Rpf2, Nog2 does not enter pre-60S subunits. Thus, Nog2 cannot be used as bait for these purifications (Zhang et al, 2007). In contrast to depletion of Rpf2, when truncated rpf2 protein is expressed (but wild-type Rpf2 is absent), Nog2 can enter assembling pre-ribosomal subunits and was used as bait for affinity-purifications (new Supplementary Figure 5c) and cryo-EM in this manuscript.

5. Supplementary Figure 4a: the authors refer to a control already published in 2007 ("Assembly factors Rpf2 and Rrs1 recruit 5S rRNA and ribosomal proteins rpL5 and rpL11 into nascent ribosomes"), but re-depicted in Figure 3. Comparing this new Nop7-TAP purification under GAL condition with the old but same purification, there is a significant difference in the pattern of co-enriched bands. It would be important to perform side-by-side a new control under the same conditions, which only would allow to directly compare these results. Supplementary Figure 4c: please include a SDS-PAGE reference for the western from the Nog2-TAP purification, similar to Figure 4a.

The new purifications depicted in Supplementary Figure 4a (new Supplementary Figure 5b) and the old purifications from Zhang *et al*., 2007, are not the same purifications. The control experiment from this publication was from cells where Rpf2 was depleted, and the new experiment represents purifications from cells in which truncated Rpf2 is expressed when endogenous Rpf2 is depleted. In addition, the pattern of bands is not as different as it first seems. We compared them side by side and all of the major bands are present in both. What makes them look different is the way the figure depicting the silver-stained gel for the control purification was edited, since the top part of the gel, containing the Rea1 band, was cropped from the picture in Zhang *et al*. At the time of publishing this paper (Zhang et al.), we were not aware of the existence of the Rea1 protein, and thus we were cropping our gels at the top, unaware that the band at the top was relevant. What also makes it look more different, is the lower yield from purifications in the original Supplementary Figure 4a.

We have now repeated purifications using the Myc-tagged truncated Rpf2 in the *rpf2Δ255-344* strain. The pattern of bands is much more comparable to the published control. Due to time constraints, we focused on Myc-tagging and experiments with newly generated strains, and this control experiment (depleting Rpf2) was not repeated. We are confident that the patterns of bands on silver stained gels are very similar and representative of Nop7-Tap purifications.

We have now added a picture of a silver-stained gel for Nog2-Tap purifications, in Supplementary Figure 4c (now Supplementary Figure 5c) since these purifications were repeated with Myc-tagged *rpf2Δ355-344* mutant strain.

6. Page 6, lanes 135-137: Include references

We apologize for this omission. Missing references are now added to the Introduction.

7. Page 7, lane 156: Properly "folded" seems not the best term for an unstructured, flexible protein extension.

We have now deleted "properly folded". The sentence now reads "…suggesting the importance of the CTD of Rpf2 in maturation of pre-60S ribosomes".

8. Page 9, lane 189-196: Despite it was previously shown that protein levels of the analysed proteins are interdependent, it would be necessary to show the stability of the Rpf2 truncated protein by a western blot analysis of cell lysates (apparently Supplementary Fig. 4a does not include whole cell lysates but only eluates). Moreover, L5 and L11 westerns (Supplementary Fig. 4a) should be improved.

As explained above (see responses to comment #2 reviewer #1 and comment #3 reviewer #2), we have addressed these comments by Myc-tagging both wild type and truncated Rpf2 and testing their stability by western blotting of whole-cell lysates (Supplementary Figure 4b). rpL5 and rpL11 western blots were also repeated and improved to the best of our ability in the time-frame allotted for the revisions of this manuscript (Supplementary Figure 5b).

9. Page 12, lanes 253-255: It is mentioned that classes C2 and C3 were combined since the difference was "only" in presence/absence of a few assembly factors. Still, it would be important to mention, which assembly factors in fact were present/absent, compared to wild-type particles.

For clarity, we have deleted the second part of that sentence. The sentence used to read: "Classes C2 and C3 were combined for the further refinement to provide an improved map (C4,

3.2 Å), as the major difference between them was only the absence or presence of a few AFs”. The sentence now reads: “Highly similar classes, C2 and C3, were combined for further refinement, providing an improved map (C4, 3.2 Å)”. Differences in classes C2 and C3 are addressed in detail later in the manuscript, in Figure 8 and in the text. That avoids distraction early in the manuscript, and the information is provided in the more logical part of the manuscript. Thank you.

10. Page 16, lanes 321-322: It is not evident that the Rpf2 CTD is directly involved in H68-70 maturation as suggested in the text. It could be also indirect via affecting the positioning of other factors necessary for this maturation (e.g. the first two alpha-helices of Nog2 that interact with this rRNA region).

We originally addressed this issue in the Discussion, but now we realize that this should be explained earlier in the manuscript. Please see a detailed response to comment #1 for the reviewer #2. We now discuss this point in the Introduction, Results and Discussion sections (page 4, lanes 131-133; page 8, lanes 293-294; page 9, lanes 360-362; page 11, 418-419).

11. Supplementary Fig. 2a: It is rather surprising that Rpf2 co-purifies the 90S factor Mpp10. This remains to be further addressed in this study. Therefore, the authors should not only analyse Tsr1-TAP and late cytoplasmic Lsg1-TAP, but also perform affinity-purification of “middle stage” (e.g. Nog2, Nsa2) and nucleolar factors (e.g. Nsa1) that are not present on 90S particles. Also loading controls (bait protein and r-protein westerns) and/or Coomassie staining would be important to validate the results

The reviewer is making a valid point about better controls, such as Nog2 and Nsa2 as representatives of middle stages of assembly, and Nsa1 as a representative of nucleolar factors. However, these data are meant to simply be used as supporting evidence for data that had been already published. Purifications with different bait proteins are notoriously time-consuming, since each bait protein yields a different number of pre-ribosomal particles. In order to obtain perfect loading for western blotting with anti-Mpp10, we would have to go through more than one set of purifications with several different strains. Thus, considering the low level of importance of these data, and abundant information about entry of Rpf2 and Rrs1 into early pre-60S subunits, we choose to delete these data. Instead, we refer to Figure 6 from Zhang *et al.,* 2007, which shows that early pre-rRNA intermediates co-purify with Rpf2-Tap and Rrs1-Tap. Supplementary Figure 3 from the same manuscript shows that members of the Rpf2 subcomplex are present in Ssf1-Tap (early) particles. In addition, more recent publications (24, 25) address this issue. Although there are slight differences in interpretations of entry points for Rpf2 and Rrs1 (either with rRNA Domain IV or IV plus V), both agree that their entry occurs during early stages of assembly, long before these proteins are visualized on assembling subunits by cryo-EM.

Based on these data, we feel confident that all members of the Rpf2 subcomplex/5S RNP enter pre-ribosomal particles very early in assembly, and that Rpf2 and Rrs1 leave particles during the lifetime of Nog2 in 60S pre-ribosomal subunits.

12. The title of Supplementary Fig. 2 should be revised, as this figure does not give information at which stage pre-60S maturation is affected (“yet truncation of its CTD affects subunit assembly at later steps”). Since there is also a nucleolar L25-GFP signal (which normally is not the case in mutants with a later nucleoplasmic defect), it is not obvious (at least from this figure) whether there is also an early pre-60S biogenesis defect.

We agree with the reviewer that the term "later" in the Figure title was very misleading, since that implies that truncation of the CTD of Rpf2 blocks late steps of assembly of the 60S subunit. It would have been more appropriate to use the term "middle" instead. However, our original Supplementary Figure 2 (now Supplementary Figure 4) has been completely rearranged and portions have been deleted and improved. The title of this figure has now changed completely, to read: "Rpf2 lacking C-terminal residues 255-344 is stably expressed, co-sediments with pre-60S subunits, and causes a block in middle stages of 60S ribosomal subunit assembly".

13. Supplementary Fig. 2c: it would be necessary to show the polysome profiles with (N-terminally) epitope tagged Rpf2 and Rpf2Δ255-344 and assess them by western blotting, in order to reveal how well the mutant is binding to the particles and to compare the free/pre-60S-bound levels of wild-type vs mutant protein. In addition, a western blot against Nog2 could clarify whether Nog2 pre-60S association in the rpf2 mutant is indeed completely unaffected as mentioned in the manuscript (e.g. on page 12, lanes 238-240).

As stated above, we have Myc-tagged both wild-type and truncated Rpf2 proteins expressed from plasmids. We grew *GAL-HA-RPF2* strains containing either plasmid in glucose (to deplete Rpf2 expressed from the genome under control of the galactose promoter), and used whole-cell extracts for sucrose gradient centrifugations. Next, we performed western blotting with fractions from these gradients using anti-myc, anti-Nog2, and several other antibodies, including antibodies against two other protein constituents of the Rpf2-subcomplex, rpL5 and rpL11. Polysome profiles are shown in new Supplementary Figure 4c, and western blots across the gradient are presented in Supplementary Figure 4d. We do not see significant changes in sedimentation of any of the tested proteins when we compare our control strain (wild type Myc-Rpf2 protein expressed, left panels) to our mutant strain (Myc-rpf2Δ255-344 truncated protein expressed, right panels). Thus, we conclude that truncated Rpf2 protein can enter pre-ribosomes at levels comparable to the wild-type Rpf2 protein, and that other tested assembly factors and ribosomal proteins are also unaffected by truncation of the CTD of Rpf2.

14. Figure 3: On the silver-stained gel only the bands corresponding to Nog2-TAP, Arx1, Sda1, Rea1 are indicated. Can also other major bands be indicated for a better understanding on the protein pattern? On page 12, lanes 246-247, it is mentioned that Nop53 is decreased and ITS2 processing is therefore affected. This is not apparent from the stained gel, where Nop53 does not significantly change, in contrast to the iTRAQ data suggested. What is the explanation for this discrepancy?

We have now labeled 20 bands on the silver stained gel in Figure 3a (new Figure 2a). As depicted in that figure, in our hands (mass-spec from bands cut from gels), Nop53 and Rsa4 migrate at the same position on 4-20% Tris-Glycine gels, and form one band just above IgG. Levels of Rsa4 increase slightly, as shown in Figure 3c (due to the absence of Rea1 that is required for removal of Rsa4), which may explain why the decrease in levels of Nop53 is not obvious. Since different proteins stain differently with silver, it may be that Rsa4 stains better than Nop53, and thus also hinders our ability to detect the decrease in Nop53 by silver staining.

In response to reviewer #3 (reviewer's comments are in *italics*):

The quality of the structural work is excellent and certainly at the level expected from readers in Nature Communications. The text is well written, and descriptions of the structures are clear. However, the main issue I see in the present form of the manuscript is that the design of the figures makes very difficult to visualize the highlighted structural differences and convey a clear message. It will be important that the design of the structural figures is improved to make a convincing case and solidify the conclusions derived from the structures. Without access to the cryo-EM maps and molecular models derived from these maps is not possible to validate some of the conclusions and statements made by the authors only based on the provided figures.

We would like to thank the reviewer for the positive feedback about the quality of our data and writing of our manuscript, and all of the constructive criticism and helpful suggestions for how to improve the way that we present our structural data. We worked hard to redesign all of our figures so that structural differences are much more obvious to visualize and understand. In addition, cryo EM maps and molecular models will have been deposited by the time the revised manuscript is submitted. Please see the response to comment #4 reviewer #1, in which we give detailed explanations of how we improved our figures, and why we believe that the new way of presenting our structural data is superior compared to our original method.

1. I think it is a great idea to add a supplementary figure to assist the comprehension of the process in the introduction describing the assembly of the 5S RNP and stable association with the ribosomal particle. However, I found very difficult to correlate the text on page 4, which refers to Suppl Fig. 1b to what is shown in this panel. Unless the reader is familiar with the maturation process of the 60S subunit, this figure is not very helpful.

Thank you. We now realize that Supplementary Figure 1b was too detailed, did not follow the flow of the Introduction, and was not helpful to a broad readership. We have completely reorganized this figure, to focus on middle stages of 60S subunit assembly, including 5S RNP rotation. Thus, this figure now serves the sole purpose to guide readers through the introductory part of the manuscript and to help them understand the part of the 60S assembly pathway relevant for this manuscript, namely middle steps of 60S ribosome subunit assembly. We hope that this figure is now much more informative.

2. Figure 4 illustrates the docking of the molecular model of State 1 Nog2 particle into the cryo-EM map obtained for the pre-ribosomal 60S particle from the C-terminal deletion mutant of rpf2 and on maps for State 1 and 3 of Nog2 particles. The difference between the 5S RNP not rotated in Stage 1 and rotated in stage 3 is not visible in the provided views in panel A. Panel C, left is also confusing. I can see there is no density representing the C-terminal end of Rpf2, but the image is very crowded. Overall this figure would benefit and become clearer if the authors were to make use of a higher level of transparencies for the cryo-EM map and also an efficient use of segmentation tools for example in the Chimera program. This would contribute to declutter the images and allow to easily see what the text is describing.

We have now completely changed this figure. We have deleted State 3 from the figure, as all of our mutant particles contain 5S RNP in a non-rotated state, due to the block in 5S RNP rotation in the *rpf2Δ255-344* mutant. Therefore, presenting State 3 certainly may cause some confusion with some readers. Please see our response to comment #4 reviewer #1 for detailed explanations of how we improved our structural figures.

3. Similarly, I don't find Fig 5A particularly effective in showing the altered configuration of H75 in the three different structures. Panel C could also be improved by zooming into the relevant regions showing differences (GTPase domain and the C-terminal domain).

This figure has also been completely redesigned and improved (please see comment #4 reviewer #1), and some parts of the figure were deleted for clarity. This figure is now split into two figures (new Figures 5 and 6), to emphasize structural changes that occur in Nog2 due to the truncation of the CTD of Rpf2. Our new Figure 6 now shows enlarged images of structural differences in relevant regions of Nog2.

4. Fig 7 is quite clear. However, I think using a light grey colour for the non-coloured regions of the maps will make a clearer figure. A good way to also show the differences between Class c2 and C3 would have been difference map analysis. The authors should consider this approach to highlight the differences between both maps.

As explained for the figures above, this figure has also been improved significantly. Depiction tool Color Zone is now used to color present densities. Please see our response to comment #4 reviewer #1 for detailed explanations how we improved our structural figures.

5. Suppl. Fig 8 would also benefit from using a light grey colour for the map and density segmentation tools could be used to easily visualize the different positions adopted by L1.

Thank you for your suggestions. Supplementary Fig. 8 (new Fig. 9) is the only figure where we decided to keep the original method to present differences between wild-type and mutant density maps. The reason is that, in this particular case, we needed to show changes in RNA helices H75-79, and using Color Zone to color densities did not work well. We have adjusted colors of all density maps to lighter colors, and believe that this figure (new Fig. 9a,b) will now be clear to all readers.

In addition, as suggested by the reviewer, we used a density segmentation tool to present differences in L1 stalk orientations between wild-type and mutant density maps (Fig. 9c).

A few additional comments non-related to the figure design:

6. Supplementary figures should include the FSC plots and local resolution analysis of the presented structures.

We thank the reviewer for this suggestion. We have now added the FSC plots and local resolution maps of C4 and E2 to new Supplementary Figures 6 and 8. For C1 and E1, in this manuscript we only focus on changes in the L1 stalk, and the resolution of those two classes is not as high as for C4 and E2 (6 Å for C1 and E1 vs 3.2 Å for C4 and 4.8 Å for E2). Therefore, due to space constraints, we omitted the FSC plots and local resolution maps for the C1 and E1 classes, since we consider them less important for the overall manuscript.

7. It is not mentioned anywhere in the manuscript that the obtained cryo-EM maps and derived molecular models have been properly deposited in the EMDB and PDB. EMDB and PDB IDs of these depositions are not provided. This is a requirement for publication in this and any other reputable journal.

The cryo-EM maps and molecular models will be deposited at the time of submission of the revised manuscript, and IDs will be updated.

8. In the 3D classification of the cryo-EM maps shown in Supple Fig 5b, the authors do not comment in what could be the origin of the 'bad particles'. What makes them bad and what do they represent? They represent about 31-32% of the data. The data set classified in Suppl. Fig 7b only shows a ~2% of bad particles. Could this difference be explained somehow? If these are particles damaged by the water-air interface, why the percentage of damaged particles is much smaller in the dataset in Suppl. Fig. 7.

We thank the reviewer for pointing this out. We originally had a section in the manuscript explaining this issue, but we deleted it due to space constraints. We now realize that this is an important point to be mentioned, and we have added it to the Discussion (page 11, lanes 428-437). Briefly, the 3D classification for the particles isolated from the *rpf2Δ255-344* mutant strain shows that several classes (A3, A5, A6, A8 and A9) are in low-resolution, and some of them (A3 and A5) are associated with missing densities for large portions of rRNA. Therefore, we referred to these particles with incomplete structures as 'bad particles'. One explanation for the "bad particles" is, as the reviewer pointed out, that the particles may have been damaged by the water-air interface. However, we believe that in the absence of the CTD of Rpf2, there is a significant turnover of particles that cannot continue down the assembly pathway. We have noticed that yields after purifications from the mutant strain are consistently lower than yields from the wild type strain. Thus, this suggests that there is significant turnover in the *rpf2Δ255-344* mutant strain, and that a majority of these "bad particles" in Rpf2-mutant strain likely represent turnover intermediates. We believe that the reason for this turnover is the unfolding of helices H68-71 in the absence of the CTD of Rpf2, *and that this specific rRNA unfolding may trigger the turnover machinery*. This also explains why these "bad particles" are not as prevalent in the strain in which Sda1 is depleted, since helices 68-71 are not misfolded in that strain. If bad particles were the result of the damage at the water-air interface, we would indeed expect to see the same percentage of "bad particles" in the *rpf2Δ255-344* truncation mutant and Sda1-depletion mutant.

This significant turnover rate was actually one of the first hints for us that this step in the assembly may be a true "checkpoint" during middle stages in 60S subunit assembly.

Reviewer #1 (Remarks to the Author):

All my concerns have been addressed and the quality of the manuscript has greatly improved. Due to the much clearer Figures, the story is much easier to read and follow now. Therefore, the manuscript is now ready to be published in Nature Communications. I just noticed one little thing: in Supplementary Figure 4d, it is not clear what is shown in the left and what in the right part. I assume it is the same arrangement as in Supplementary Figure 4c, but the strains should definitely also be indicated in S4d.

Reviewer #2 (Remarks to the Author):

The authors have made a serious effort to improve the m/s according to the previous points raised by all three reviewers. In our case, however, two substantial points remain to be answered.

In the revised manuscript, the authors still assign the primary role of the Rpf2 CTD to be directly involved in the structural H68-70 maturation and "checkpoint" control of the pre-60S biogenesis pathway. However, this appears to be overstated, as there is still a lack of convincing support for this scenario, as already outlined in our previous review.

1. The authors should seriously revise their abstract with the focus on the main and clear-cut results leaving out the speculative aspects.

2. The main structural differences of H68-70 are observed just in sub-classes of Nog2-associated particles, obtained by using the Rpf2-CTD mutant, Rpf2Δ255-344; the yields of pre-ribosomal particles purified from the rpf2Δ255-344 mutant were significantly lower than from the wild-type cells, as authors themselves state. Thus, it is possible that only a small population of pre-ribosomes from these mutant cells are in this particular state while another, much larger pool, could be stuck in (an)other state(s) and degraded. If true, the Rpf2 C-terminus would have a functional role in other steps of 60S assembly as well. It is therefore important to analyze earlier pre-60S particles in this Rpf2Δ255-344 mutant (isolated by appropriate baits), as already suggested during the first round of review.

3. Along these lines, our previous suggestion to check the stability of the 5S RNP members in their Nog2 purifications addressed via Western blot for Myc-Rpf2Δ255-344, Rrs1-HA, Rpl5, Rpl11 (shown in Supplementary Figures 5b, c), are not convincing, mainly due to the poor quality of these Westerns. There are also significant differences between Fig. 4a, Rrs1-HA Western band, and the revised figure (now Fig. 5b) with the same Rrs1-HA band. In one case there is a strong signal, in the other case there is basically no signal. It further appears that the L5 signal is decreased in the case of the rpf2 mutant. The authors could perform Northern analysis for the 5S rRNA to address the question of whether the 5S RNP is decreased in mutant particles (e.g. for Supplementary figures 7b, f).

Reviewer #3 (Remarks to the Author):

After reviewing this new version of the manuscript and read the letter in response to reviewer comments I must say that the authors put a significant amount of thought and effort into addressing not only my comments but also those from reviewer 1 and 2.

I think this new version of the manuscript is significantly improved. The data and structures are much more convincing in the new way the figures have been designed.

I would support the publication of this new version of the manuscript as it is without further changes.

In response to Reviewer #1:

All my concerns have been addressed and the quality of the manuscript has greatly improved. Due to the much clearer Figures, the story is much easier to read and follow now. Therefore, the manuscript is now ready to be published in Nature Communications. I just noticed one little thing: in Supplementary Figure 4d, it is not clear what is shown in the left and what in the right part. I assume it is the same arrangement as in Supplementary Figure 4c, but the strains should definitely also be indicated in S4d.

We thank the reviewer for helping us significantly improve the manuscript by providing very useful comments and suggestions. We are glad that we were able to address all of the reviewer's concerns.

Yes, thank you for noticing these missing labels. We have now added them to Supplementary Figure 4.

In response to Reviewer #2:

The authors have made a serious effort to improve the m/s according to the previous points raised by all three reviewers. In our case, however, two substantial points remain to be answered.

In the revised manuscript, the authors still assign the primary role of the Rpf2 CTD to be directly involved in the structural H68-70 maturation and "checkpoint" control of the pre-60S biogenesis pathway. However, this appears to be overstated, as there is still a lack of convincing support for this scenario, as already outlined in our previous review.

Regarding the reviewer's point about direct involvement of the CTD of Rpf2 in maturation of helices H68-H70, we must respectfully disagree. This extension lies *immediately adjacent to H68-H70*, as shown in our Supplementary Fig. 2b, 2d, and in Wu et al, 2016. Thus, *it is very likely, and certainly not surprising*, that deleting the CTD of Rpf2 would prevent conformational maturation of these helices. However, to address this major concern, we explained in the first revised version of the manuscript the possibility that the effect on H68-70 could instead occur through changes in Nog2 conformation, and might not be *directly caused by the absence of the CTD of Rpf2*. In this second revised version of the manuscript, we also elaborate even further, (pages 9-10, lanes 369-372) that the H68-H70 can also be affected "… *by some other, still undiscovered path. Regardless, any perturbance in the neighborhood of helices H68-70 may cause lack of conformational maturation of these helices, and most likely causes a block in 60S subunit assembly*."

Additionally, we have made several changes throughout the manuscript to further address these concerns.

(1) We have re-worded sections in the Introduction and Results, so that they more accurately describe what we have observed in the *rpf2Δ255-344* mutant strain.

(3) We stayed away from speculations and hypotheses in the Introduction and Results section of the manuscript. We, however, believe that the Discussion section is an appropriate place for some level of speculation, as long as it is clear to readers that we are stating an opinion, not a proven fact. We adjusted the Discussion section to make certain that it is clear what is speculative and what is factual.

(4) We have deleted the word "checkpoint" throughout the manuscript.

1. The authors should seriously revise their abstract with the focus on the main and clear-cut results leaving out the speculative aspects.

We have completely rewritten the Abstract section of the manuscript. We deleted the word "checkpoint", and we stated the data without any speculation. When we state that the truncation of the CTD of Rpf2 affects proper folding of helices H68-H70, as well as binding or conformation of some assembly factors, we do not consider that "speculations", since that is what our data show. Based on known functions of those affected proteins in 60S subunit assembly, we conclude which steps may be blocked. We do not include speculations about the interconnectedness of any assembly steps in the Abstract.

We hope that the Abstract now addresses the reviewer's concerns.

2. The main structural differences of H68-70 are observed just in sub-classes of Nog2-associated particles, obtained by using the Rpf2-CTD mutant, Rpf2Δ255-344; the yields of pre-ribosomal particles purified from the rpf2Δ255-344 mutant were significantly lower than from the wild-type cells, as authors themselves state. Thus, it is possible that only a small population of pre-ribosomes from these mutant cells are in this particular state while another, much larger pool, could be stuck in (an)other state(s) and degraded. If true, the Rpf2 C-terminus would have a functional role in other steps of 60S assembly as well. It is therefore important to analyze earlier pre-60S particles in this Rpf2Δ255-344 mutant (isolated by appropriate baits), as already suggested during the first round of review.

To the reviewer's first point, about degradation: We now see that the way we explained turnover rate in the *rpf2Δ255-344* mutant strain (page 11, lanes 428-437 in the first revised version of the manuscript) was potentially misleading and left an impression that the majority of particles are degraded, as the reviewer states above *("Thus, it is possible that only a small population of*

pre-ribosomes from these mutant cells are in this particular state while another, much larger pool, could be stuck in (an)other state(s) and degraded").

However, we do not believe that is the case. In order to clearly explain our reasoning, we first need to point out that we failed to make an important distinction between two categories of Nog2 particles that are turned over in the *rpf2Δ255-244* strain. We hope that we have now clarified this issue, as described below.

Category 1: The first category of turned over particles represents those whose turnover affects (decreases) the yields of our purifications. Thus, we never detect them by silver staining of SDS-PAGE, western blots, mass-spec, or visualize them by cryo-EM. Therefore, the challenge in trying to study these particles in order to understand whether *rpf2Δ255-344* causes an early block is that they simply are not available for further study by biochemical methods or cryo-EM. Importantly, in the paragraph we are referring to, we state that "*The yields of pre-ribosomes purified from the rpf2Δ255-344 mutant strain were consistently lower than those from the wild-type strain. This suggests that there is significant turnover in the rpf2Δ255-344 mutant strain*". This leaves the impression that ***the majority*** of particles are turned over. In fact, the yield obtained from the *rpf2Δ255-344* mutant strain was very often ***only*** 20-30% lower than that obtained using the wild-type control strain, and in a few cases, comparable to wild-type levels (for example, the silver staining of the gels in Figure 2a was not adjusted for yield at all). Thus, using words "consistently" and "significant" was not the right choice. Therefore, we have rewritten these sentences to more accurately describe our observations. We now state (page 11, lines 440-443): "The yields of pre-ribosomes purified from the *rpf2Δ255-344* mutant strain were slightly lower than those from the wild-type strain. This suggests that a small percentage of particles are turned over in the *rpf2Δ255-344* mutant strain before they could be detected by SDS-PAGE or visualized by cryo-EM."

We apologize that we have unintentionally misled the reviewer. This was an oversight and it is now corrected.

Category 2: The second category of particles are "bad particles" visualized by cryo-EM. Clearly, these particles did not affect the yield of purified assembling 60S subunits, since they were mostly intact at the time when they were used for cryo-EM. Importantly, in all of these "bad particles" (A3, A5, A6, A8, and A9), it is clear that the Rpf2-subcomplex is already anchored to the remainder of the assembling subunit, together with rRNA domain V. ***Thus, 100% percent of particles visualized by cryo-EM are in the same state*** (Supplementary Fig. 6b), with the 5S RNP already anchored. We suspect that these particles are destined for turnover (at least A3 and A5 are missing densities for large portion of the rRNA, suggesting that the particles could be the turnover intermediates with some rRNA sequence already degraded). Importantly, the reason for this turnover is not lack of anchoring of the Rpf2 subcomplex***. Thus, the majority of particles that are purified using Nog2 as a bait are in the same state***, not only the small population, as the reviewer is suggesting.

To the reviewer's second point, about an earlier block in assembly: The reviewer is raising the important possibility that the absence of the CTD of Rpf2 is causing a defect in earlier stages of assembly (in addition to the later defects that we describe), resulting in the subsequent turnover of those particles (represented by the first category of particles explained above).

We believe that we have already performed an experiment to test this idea, using Nop7 as a bait to affinity purify preribosomes from the mutant. Nop7 has been used routinely by our lab both as a (1) tool to detect accumulation of early assembling intermediates, and (2) a marker for instability/turnover of assembling particles.

(1) Nop7 and Rpf2 enter preribosomes at a very early stage and exit from them just before nuclear export. We frequently begin our studies of mutants with affinity purifications of preribosomes using TAP-tagged Nop7 or Rpf2 as baits, because these purifications "report" effects of mutations on most nuclear stages of large subunit assembly. The most prominent "marker" for a block in earlier stages of 60S subunit assembly, visible by silver staining, is accumulation of early entering/early leaving assembly factors (for example Rrp5, Noc1 and Noc2) several of which (conveniently) migrate with the high molecular weight markers on SDS-PAGE gels. Please refer to the following figures as references: Figure 2A in Sahasranaman et al, 2011; Figure 2A (left two panels for GAL-L3 and GaL-L18) in Gamalinda et al, 2014; Figure 5A in Jakovljevic et al, 2012; Figures 2A, 3A in Biedka et al, 2018).

It is obvious that such a trend is not seen with the samples from the *rpf2Δ255-344* mutant strain where Nop7-TAP was used as a bait (Supplementary Figure 5b, left).

(2) As we have reported previously (Miles et al, 2005), Nop7 part of a subcomplex with Ytm1 and Erb1. This subcomplex has a high rate of disassociation from assembling 60S subunits in ***mutants that cause turnover***. Please refer to the following figures as references: Figure 9A (showing dissociation of the Nop7-subcomplex from the assembling subunits in the *rrp1-1* and *nop4-3* mutants) in Miles et al, 2005; Figure 6D (showing turnover of particles in *rrp1-1* by pulse-chase) in Horsey et al, 2005; Figure 2C (showing dissociation of the Nop7-subcomplex from assembling subunits when an early assembly factor (Rlp7) is depleted, the absence of which causes turnover) in Sahasranaman et al, 2011).

Our data in Supplementary Figure 5b of this manuscript ***do not*** show accumulation of the Nop7/Ytm1/Erb1 subcomplex.

Thus, we have not detected any evidence for instability of earlier particles, or of accumulation of earlier particles, in samples isolated from the *rpf2Δ255-344* mutant strain, using Nop7-TAP as a bait.

Together, based on all data presented, we strongly argue that, like the depletion of Rpf2, truncation of the CTD of Rpf2 does not cause a significant/measurable block in earlier stages of 60S subunit assembly, and that turnover occurs only once the Rpf2-subcomplex has stably anchored and Nog2 is bound to assembling particles, which occurs during middle stages of assembly.

However, to be completely transparent with readers, we realize that we must raise the point that the absence of the CTD of Rpf2 may affect earlier steps, even if the probability of that scenario

is low. Thus, we have now added this statement in the Introduction (page 4, lines 129-131), and in the Discussion sections (page 9, lanes 362-363).

Finally, to make it completely clear what the focus of the manuscript is, we have now made several adjustments to the manuscript about the goal we tried to achieve. We now state in the Abstract, Introduction, and Results that we specifically generated this truncation in order to test whether its absence prevents stable anchoring of the Rpf2 subcomplex and rRNA domain V, or whether it affects steps after the anchoring has occurred, namely 5S RNP rotation and export (page 2, lines 35-37; page 4, lanes 126-129; page 4, lane 148).

In summary, we strongly believe that the above statements address the concerns of the reviewer, and we do not find it necessary to perform any more purifications with early assembly factors as baits. In addition, in the very unlikely scenario that there may be an effect in the very early stages of assembly, seen only using very early entering/leaving assembly factors, there is a strong probability that those particles, as the reviewer states, would be degraded. Thus, that would prevent any further biochemical or structural investigation.

3. *Along these lines, our previous suggestion to check the stability of the 5S RNP members in their Nog2 purifications addressed via Western blot for Myc-Rpf2Δ255-344, Rrs1-HA, Rpl5, Rpl11 (shown in Supplementary Figures 5b, c), are not convincing, mainly due to the poor quality of these Westerns. There are also significant differences between Fig. 4a, Rrs1-HA Western band, and the revised figure (now Fig. 5b) with the same Rrs1-HA band. In one case there is a strong signal, in the other case there is basically no signal. It further appears that the L5 signal is decreased in the case of the rpf2 mutant. The authors could perform Northern analysis for the 5S rRNA to address the question of whether the 5S RNP is decreased in mutant particles (e.g. for Supplementary figures 7b, f).*

We are sorry that the reviewer is finding our new data of poor quality. We only had one chance to perform purifications and western blots shown in Supplementary Figures 5b,c before our lab was closed due to the COVID-19 outbreak, since we were busy performing other requested experiments. It is correct that the signal for Rrs1-HA is very weak compared to the previous western blotting data in the original manuscript. This is most likely due to the fact that we re-used anti-HA antibody, since we were doing several rounds of western blots in a period of a few days. Regardless, even though the signal is faint, it is still visible that there is no decrease in levels of Rrs1-HA in the absence of the CTD of Rpf2. The slight decrease in levels of rpL5, although probably due to technical issues, is not significant when compared to the striking decrease seen in the GAL-RPF2 strain where Rpf2 is depleted (Figure 3A in Zhang et al, 2007, see insert below).

Thus, the data are consistent with our observations that, contrary to what occurs upon depletion of Rpf2, all of the constituents of the 5S RNP complex are able to enter preribosomal particles in the *rpf2Δ255-344* mutant strain.

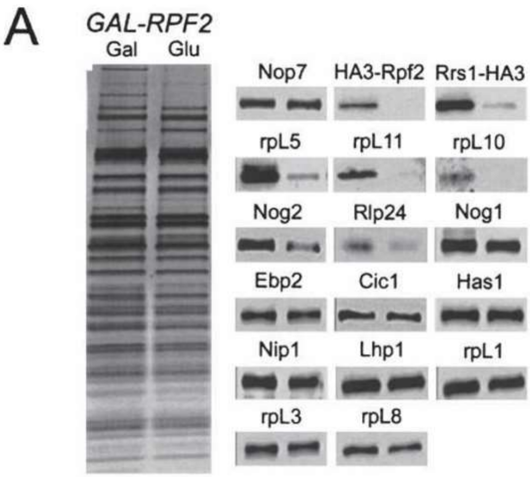

While Supplementary Figures 5b,c show the ability of these proteins to stably assemble into particles purified using either Nop7 or Nog2 as baits, these figures do not address the stability of these proteins in cells per se. The stability of myc-rpf2Δ255-344, rpL5 and rpl11, together with controls, was examined in Supplementary Figures 4b (western blots of whole cell lysates) and 4d (western blots using gradient fractions), as requested by two reviewers. We strongly believe that the data presented in those figures is extremely clear, and that there is no evidence for any instability and turnover of any of the proteins tested. Since all constituents of the Rpf2 subcomplex are interdependent for their stability (Figure 4 (showing westerns with whole-cell-lysates) in Zhang et al, 2007, see insert below), we conclude that Rrs1 is also stable in the *rpf2Δ255-355* mutant strain. Together, we think that the combination of western blots from whole cell extracts and purified particles together is very convincing.

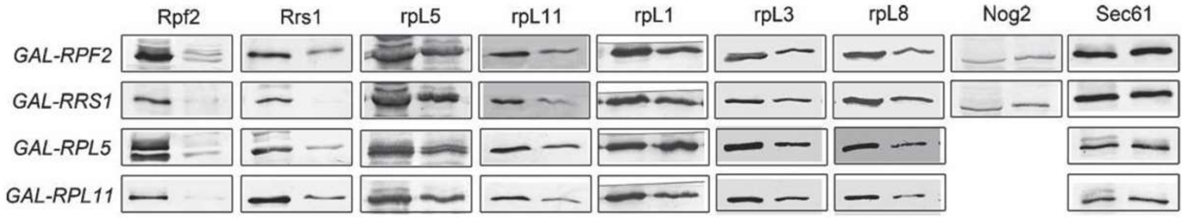

As for 5S rRNA levels, we are not certain why the reviewer mentioned Supplementary Figures 7b, f here. These figures contain data for *GAL-SDA1* and *rpf2-2* strains, not for the *rpf2Δ255-355* strain. Our data for 5S rRNA levels from whole cell lysates are already shown in Supplementary Figure 4f, and the levels of 5S rRNA did not change significantly.

Performing purifications to repeat the western blot for Rrs1-HA or to isolate 5S rRNA from purified particles are not options for us at this point. Considering that there are several phases for reopening our university due to COVID-19, in the best case scenario we will not be back to the laboratory until at least 5-6 weeks from now, but it may take 8-10 weeks or longer. Performing the

experiments would take another 3-4 weeks since all of our strains are frozen and we don't have any magnetic beads in the lab. That would result in a significant delay in publication of this manuscript. Since we have already addressed in the manuscript the possibility that there may be some earlier defect that we cannot see with the tools available, we hope that the reviewer will accept all of our explanations and edits to the manuscript, and that this will be sufficient for acceptance of the manuscript for publication.

In response to Reviewer #3:

After reviewing this new version of the manuscript and read the letter in response to reviewer comments I must say that the authors put a significant amount of thought and effort into addressing not only my comments but also those from reviewer 1 and 2.
I think this new version of the manuscript is significantly improved. The data and structures are much more convincing in the new way the figures have been designed.
I would support the publication of this new version of the manuscript as it is without further changes.

We thank the reviewer for helping us improve this manuscript significantly, with very useful comments and suggestions.
